# Group 10 Metal Allyl Amidinates: A Family of Readily Accessible and Stable Molecular Precursors to Generate Supported Nanoparticles

**DOI:** 10.1021/jacsau.3c00334

**Published:** 2023-08-01

**Authors:** Christian Ehinger, Xiaoyu Zhou, Max Candrian, Scott R. Docherty, Stephan Pollitt, Christophe Copéret

**Affiliations:** † D-CHAB, ETH Zürich, Vladimir−Prelog-Weg 1−5, 8093 Zürich, Switzerland; ‡ PSI, Forschungsstrasse 111, 5232 Villigen, Switzerland

**Keywords:** organometallic precursors, surface organometallic chemistry, atomic layer deposition, nanoparticles, heterogeneous catalysis

## Abstract

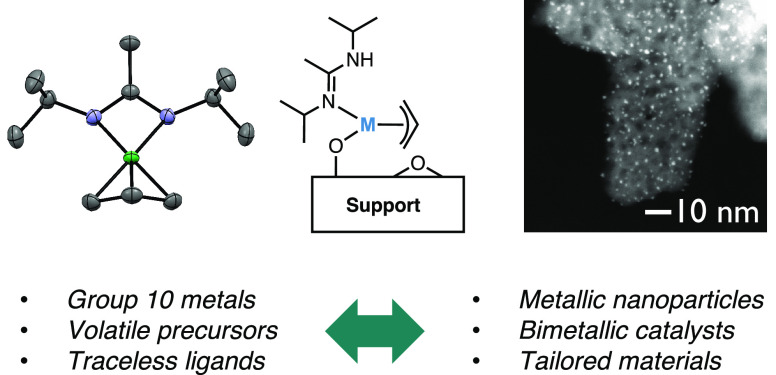

The synthesis of well-defined materials as model systems for catalysis and related fields is an important pillar in the understanding of catalytic processes at a molecular level. Various approaches employing organometallic precursors have been developed and established to make monodispersed supported nanoparticles, nanocrystals, and films. Using rational design principles, a new family of precursors based on group 10 metals suitable for the generation of small and monodispersed nanoparticles on metal oxides has been developed. Particle formation on SiO_2_ and Al_2_O_3_ supports is demonstrated, as well as the potential in the synthesis of bimetallic catalyst materials, exemplified by a PdGa/SiO_2_ system capable of hydrogenation of CO_2_ to methanol. In addition to surface organometallic chemistry (SOMC), it is envisioned that these precursors could also be employed in related applications, such as atomic layer deposition, due to their inherent volatility and relative thermal stability.

## Introduction

**Figure 1:**
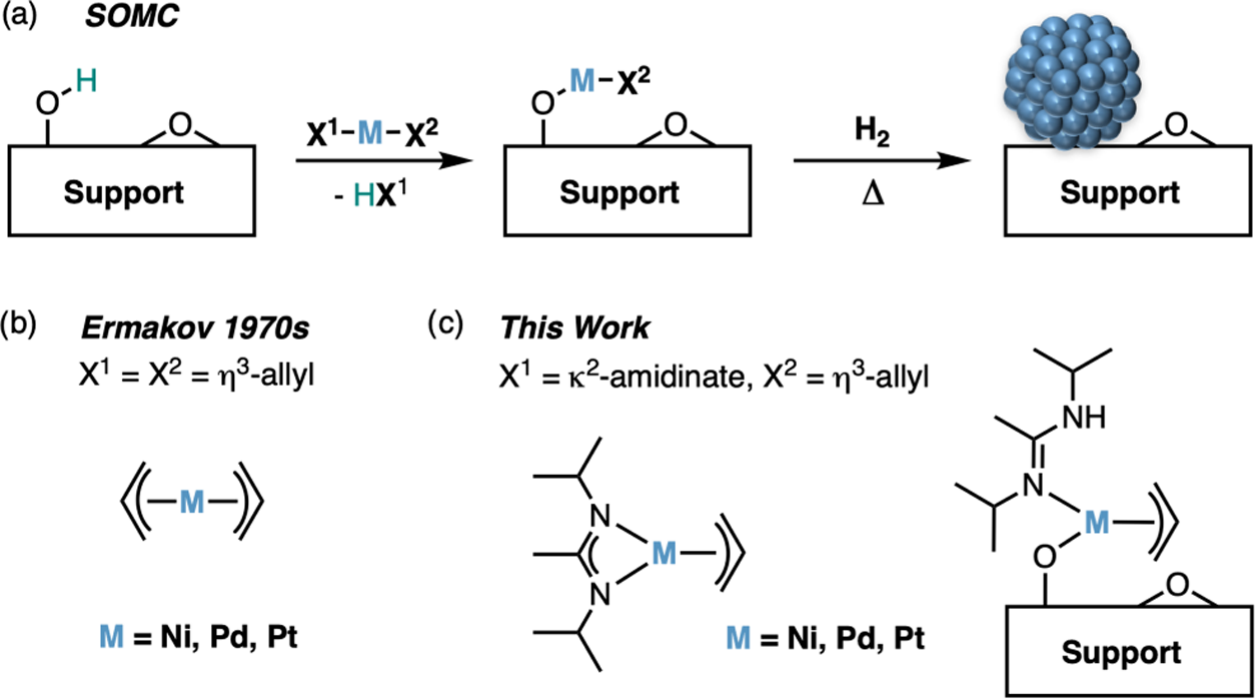
(a) General synthesis of supported metallic nanoparticles via SOMC. Ligand X^1^ is basic and deprotonates the surface OH groups, and ligand X^2^ needs to be readily removed under H_2_. (b) Bis-η^3^-allyl-M(II) has been used as a precursor in the 1970s. (c) In this work, allyl-amidinate complexes of group 10 metals are used as precursors to generate supported metal nanoparticles.

The generation of metallic nanoparticles, nanocrystals, and films has been a highly dynamic research area due to their uses and developments for various applications, including imaging, sensing to catalysis, etc.^1−3^ Obtaining the desired metallic materials, free of contaminants, is essential for many applications. In that context, the use of organometallic or metalorganic precursors has shown to be advantageous.^4,5^ Key criteria for choosing precursors include, in particular, specific reactivity (e.g., ease of reduction or thermolysis), relative stability, and preparation scalability, along with other physicochemical properties, such as volatility or solubility in specific solvents, to name but a few.^6,7^ Overall, this decision boils down to choosing a suitable set of ligands that, at the same time, endow their stability and ensure that they can be readily removed to generate the desired (nano-)material, free of organic or other contaminants (e.g., halides or residual carbonaceous species). Of various synthetic methodologies interested in generating clean materials, surface organometallic chemistry (SOMC) has emerged as a privileged approach to provide better-defined catalytic materials, such as supported nanoparticles with tailored interfaces and compositions (Figure 1a).^5,8^ SOMC enables the interrogation of promoter effects and the role of interfacial Lewis acidic sites and alloying in heterogeneous catalysis.^6,9−11^ For SOMC, similar to atomic layer deposition (ALD), one important criterion is to identify precursors that can react selectively with surface functionalities, in particular, surface OH groups. Furthermore, volatile precursors can also be of interest for gas-phase deposition. All in all, it is not surprising that organometallic compounds have been privileged molecular precursors due to their relatively high reactivity toward Brønsted acidic OH groups. The ligand residues in the obtained material are removed under mild treatment conditions (hydrolysis or hydrogenolysis), releasing only hydrocarbon byproducts that do not interact with surfaces; in addition, lighter homologues can often be sublimed at relatively low temperatures.

While readily available and reactive for early transition-metal compounds (group 4–6/7), metal precursors with all desired properties are far scarcer for later, and in particular, precious 4d and 5d transition metals that often contain more complex stabilizing ligands such as phosphines that are not as easily removed.^12^ Additionally, group 10 transition-metal alkyl precursors are typically less reactive toward OH groups, even leading to alternative grafting mechanisms in some cases (e.g., immobilization by involvement of siloxane bridges rather than OH groups).^13^ Overall, these precursors are either too inert or too reactive and unstable. The prominent examples are allyl derivatives; while convenient for early transition metals to generate well-defined surface species, the corresponding derivatives of group 10, the bis-η^3^-allyl-M(II) (M = Ni, Pd, Pt) compounds, are thermally unstable.^14−17^ They spontaneously decompose at ambient temperature, thus encumbering their general application to SOMC and related approaches, even if used in the pioneering work of Ermakov and Girolami et al. to generate supported nanoparticles and thin films, respectively (Figure 1b).^18−20^

In contrast, amidinate ligands are known to form stable complexes with most transition metals.^21^ They are a popular class of ligands for ALD applications thanks to their basicity, volatility, and ease of decomposition upon reactive gas treatment.^22^ Amidinate ligands adopt a bidentate κ^2^-mode in monomeric metal complexes, paralleling η^3^-allyl ligands. This prompted us to combine these two ligands and investigate heteroleptic allyl-amidinate complexes of group 10 as SOMC precursors and to explore their reactivity.

Here, we present the synthesis of group 10 allyl-(*N-N*’-diisopropyl)acetamidinates M(η^3^-allyl)(DIA) (M = Ni, Pd, Pt) as readily accessible molecular precursors for SOMC (Figure 1c). We demonstrate their reactivity toward prototypical oxide supports (SiO_2_ and Al_2_O_3_) and investigate the grafting mechanism of these heteroleptic compounds, showing that the amidinate is the only reactive moiety. We also demonstrate their proficiency to generate supported mono and bimetallic nanoparticles cleanly, as well as the synthesis of efficient hydrogenation catalysts based on PdGa/SiO_2_ for methanol synthesis from CO_2_.

## Results and Discussion

### Synthesis

**Scheme 1:**
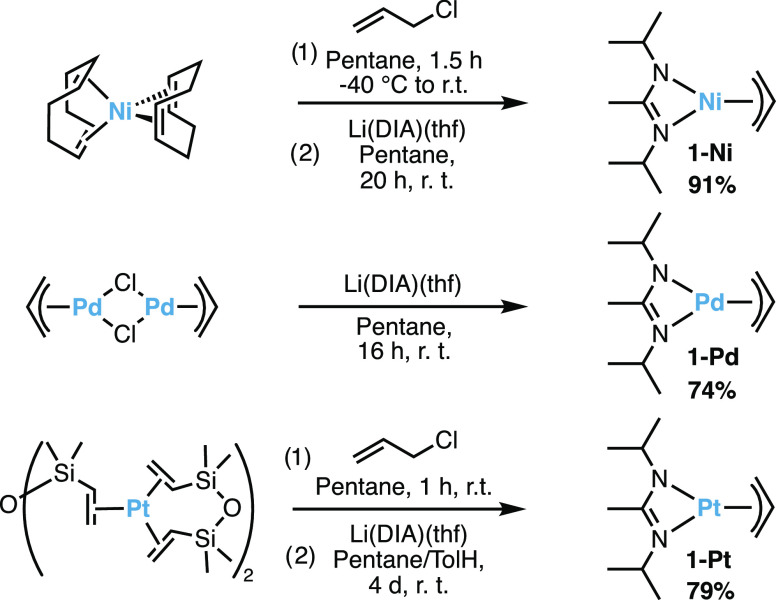
Synthesis of 1-Pd, 1-Ni, and 1-Pt

We started with the Ni derivative, 1-Ni, utilizing a two-step one-pot reaction (Scheme 1). The reaction of Ni(COD)_2_ (COD = 1,5-cyclooctadiene) in pentane with an excess allylchloride affords {Ni(η^3^-allyl)Cl}_2_ in situ.^23,24^ The further reaction with Li(DIA)(thf) yields the desired product in 91% yield as an orange–red microcrystalline solid after sublimation (10^–3^ mbar, 45 °C)—see Experimental Section for more details.

The ^1^H NMR spectrum of 1-Ni in C_6_D_6_ (Figure S1) shows a triplet of triplets at 4.84 ppm for the central proton of the allyl, a heptet at 3.12 ppm for the central proton on the amidinate *iso*-propyl group, doublets at 2.88 and 1.66 ppm for the terminal protons on the allyl ligand that are *syn* and *anti* to the central C–H bond, respectively, a singlet at 1.39 ppm for the CH_3_ group bound to the quaternary carbon, and two doublets at 1.04 and 0.84 ppm for the diastereotopic terminal protons on the *iso*-propyl group. The observed signals are consistent with 1-Ni being diamagnetic and adopting a pseudo-square-planar geometry.

**Figure 2:**
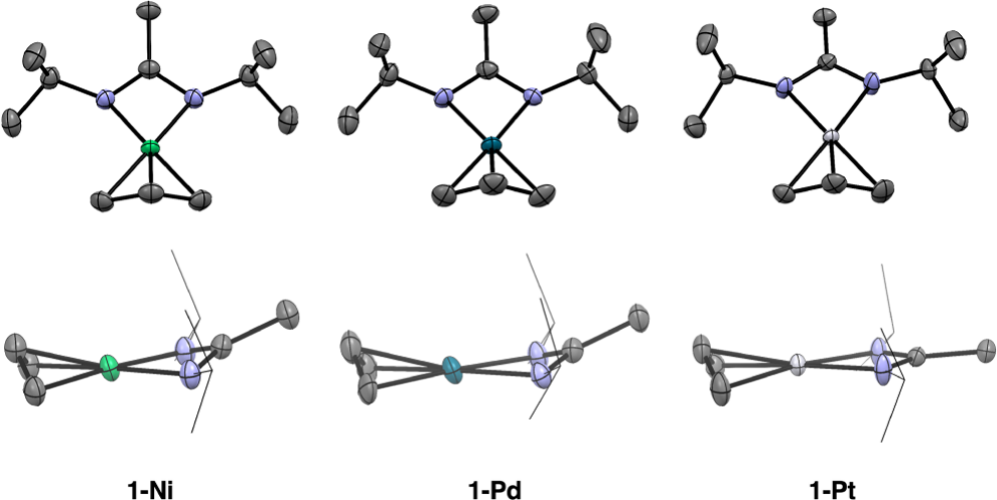
X-ray structures of 1-Ni, 1-Pd, and 1-Pt. Thermal ellipsoids are drawn at 50% probability level. H-atoms and the second molecule in the unit cell are omitted for clarity.

Single crystals of 1-Ni were obtained from cooling a concentrated pentane solution to −40 °C. Single-crystal X-ray diffraction confirmed the pseudo-square-planar coordination geometry of the complex, where the amidinate ligand is slightly puckered (Figure 2 and Table 1, entry 1). The bond lengths and torsion angle of DIA are comparable in magnitude with the respective homoleptic analogues (Ni(η^3^-allyl)_2_ and Ni(DIA)_2_, Table 1, entries 2 and 3).^25,26^ The central carbons of the two ligands are oriented *syn* to each other, whereas in the homoleptic complexes, the two ligands are *anti*. The *iso-*propyl groups of the amidinate ligand in 1-Ni are facing toward the metal center, whereas in Ni(DIA)_2_, they are alternatingly facing in and out to minimize steric interactions.

**Table 1:** Selected Bond Lengths and Torsion Angle^25,26^

complex	M–C^terminal^ (Å)^a^	M–C^central^ (Å)^a^	M–N (Å)^a^	N–N torsion (deg)^a^
1-Ni	2.00	1.97	1.93	155.8
Ni(η^3^-allyl)_2_^b^	2.03	1.98		
Ni(DIA)_2_			1.92	158.3
1-Pd	2.12	2.12	2.09	157.4
1-Pt	2.12	2.09	2.08	176.7

a For 1-Pd and 1-Pt, there was disorder in one of two molecules in the unit cell, and the values of the one without disorder are reported. For 1-Ni, the average of two is reported (no disorder).

b The structure of Ni((η^3^-allyl))_2_ was determined by neutron diffraction.

Next, we explored the preparation of the corresponding Pd and Pt complexes, 1-Pd and 1-Pt. Upon the reaction of commercially available {Pd(η^3^-allyl)Cl}_2_ with Li(DIA)(thf), 1-Pd was obtained in 74% yield as a bright yellow solid after sublimation (10^–3^ mbar, 55 °C). The ^1^H NMR spectrum of 1-Pd in C_6_D_6_ (Figure S4) shows analogous signals to 1-Ni consistent with an isostructural coordination geometry. For 1-Pt, we resorted to a two-step one-pot synthesis starting from platinum(0)-1,3-divinyl-1,1,3,3-tetramethyldisiloxane (Karstedt’s catalyst). The addition of an excess of allylchloride in pentane resulted in the precipitation of a yellow solid. Optical appearance and low solubility in common organic solvents are consistent with the intermediate formation of {Pt(η^3^-allyl)Cl}_4_.^27^ The further reaction with Li(DIA)(thf) and purification by sublimation (10^–3^ mbar, 60 °C) affords 1-Pt in 79% yield. The ^1^H NMR spectrum of 1-Pt in C_6_D_6_ (Figure S7) shows strong secondary *J*-coupling to ^195^Pt (*I* = 1/2, 33.8% abundance), complicating its interpretation. However, the ^13^C NMR spectrum (Figure S8) exhibits resolved couplings to ^195^Pt and is consistent with the ^13^C NMR spectra of 1-Ni and 1-Pd (Figures S2 and S5). Notably, the difference in color observed (orange–red, yellow, white, respectively) is in line with crystal field theory predicting an increased orbital splitting for higher main quantum number N of the transition metal while maintaining the same coordination environment. The thermal stability of these complexes was further assessed by TGA measurements, indicating that decomposition at appreciable rate starts at 111, 159, and 190 °C for 1-Ni, 1-Pd, and 1-Pt, respectively (Figures S45–S47). These temperatures lie well above their respective sublimation temperatures under reduced pressure (10^–3^ mbar). TGA also indicates a clean decomposition of the organic fragments, as the residual masses after completion of the temperature program lie very close to the expected metal content.

Single crystals of 1-Pd and 1-Pt were obtained in the same way as 1-Ni, and single-crystal X-ray diffraction confirmed their isostructural geometry (Figure 2).

The bond lengths of 1-Pd are almost the same as in 1-Pt and slightly longer than in 1-Ni, which is ascribed to differences in ionic radii. The torsion angle of the amidinate ligand of 1-Pd (dihedral angle around N–N axis) is slightly bent like in 1-Ni, whereas it is almost flat for 1-Pt (visible in side view in Figure 2). DFT geometry optimizations were performed to assess whether this effect arises (solely) from crystal packing. The experimentally observed bond lengths were reproduced within 0.01 Å, and the same trend regarding the N–N dihedral angle was observed, albeit less pronounced (1-Ni 164.2°, 1-Pd 165.4°, 1-Pt 173.2°). Thus, the torsion around the N–N axis likely arises from an electronic stabilization.

### Surface Chemistry

**Figure 3:**
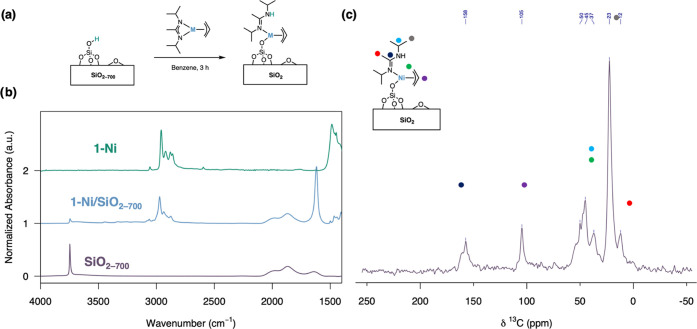
(a) Grafting mechanism of amidinate precursors on SiO_2–700_. (b) IR Spectra of SiO_2-700_, 1-Ni/SiO_2_, and 1-Ni. The IR spectra of SiO_2-700_ and 1-Ni/SiO_2_ were measured in transmission, and the absorbance is normalized with respect to the siloxane band at 1900 cm^–1^. The IR spectrum of 1-Ni was measured with ATR-FTIR. Transmission IR spectra of 1-M/SiO_2_ (M = Pd, Pt) are shown in the SI (Figures S18 and S19). (c) Solid-state MAS ^13^C NMR spectrum of 1-Ni/SiO_2–700_ with assignments based on the molecular precursor. Additional solid-state NMR spectra are shown in the SI (Figures S12–S15).

The reactivity toward oxide surfaces was investigated by contacting a benzene solution of complex 1-Ni with SiO_2–700_ (SiO_2_ partially dehydroxylated at 700 °C, OH density ca. 1 OH/nm^2^, 1-Ni/OH ca. 9:10). Within ca. 1 h, the solution was colorless, whereas the silica material turned orange. Analysis of the grafting solution by ^1^H NMR showed no residual complex remaining in solution and no (protonated) ligand was released in the solution phase upon immobilization. Monitoring the grafting process by IR spectroscopy (Figure 3b), it is evident that the OH groups are mostly consumed; the free OH groups present SiO_2–700_ (O–H stretching band at ca. 3750 cm^–1^) are strongly diminished in the grafted materials (1-M/SiO_2–700_), suggesting grafting via deprotonation, while organic fragments are observed from the emergence of C–H stretching bands (around 3000 cm^–1^). Several weak bands appear in the region of the N–H stretching frequency (3200–3400 cm^–1^), suggesting the protonation of the DIA ligand rather than allyl. Furthermore, a strong band emerges at ca. 1620 cm^–1^ upon grafting that is not present in the molecular compound, consistent with the C=N double bond forming upon protonation of the amidinate ligand. This is in line with the proposed grafting mechanism shown in Figure 3a.

In addition, thanks to the high volatility of these nickel precursors, gas-phase deposition was also evaluated. 1-Ni, 1-Pd, and 1-Pt were deposited on SiO_2–700_ under reduced pressure (10^–6^ mbar) via sublimation. The IR spectra of the resulting materials reveal that OH groups are consumed (Figures S24–S26), and the observation of the same characteristic bands after grafting in solution is consistent with an identical grafting mechanism in all cases. To further corroborate the structure of the surface site, we turned to magic-angle-spinning solid-state NMR spectroscopy. ^13^C-MAS SSNMR spectroscopy of 1-Ni/SiO_2–700_ (Figure 3c) confirmed that both the η^3^-allyl and the amidine ligand are present in the grafted materials. In the ^13^C-MAS SSNMR spectrum of 1-Ni/SiO_2–700_, the signal around 158 ppm corresponds to the quaternary carbon of the amidine, and the signal around 105 ppm is assigned to the central carbon of the allyl ligand. The terminal carbon atoms of the allyl and the central carbon of the *iso*-propyl group are overlapped (ca. 30–60 ppm)—several signals are observed in this region due to the desymmetrization upon grafting. CH_3_ groups of *iso*-propyl and CH_3_ bound to the quaternary carbon are observed at 23 and 12 ppm, respectively.

On Al_2_O_3–600_ (γ-Al_2_O_3_ partially dehydroxylated at 600 °C), 1-Ni also immobilized without loss of ligand, and the changes in the IR spectrum (Figure S21) are consistent with deprotonation of OH groups by the amidinate ligand, as evidenced by the diminished intensity of OH groups, in particular, bound to 5- and 6-fold coordinate Al sites on the surface (3776 and 3731 cm^–1^, respectively).^28−30^ In addition to C–H stretching bands around 3000 cm^–1^, a C=N stretching band (ca. 1600 cm^–1^) and N–H stretching bands (3400–3200 cm^–1^) emerged, indicating that grafting occurs via a similar pathway as discussed for SiO_2–700_. This is further supported by ^13^C-MAS SSNMR spectroscopy of 1-Ni/Al_2_O_3–600_ (Figure S14).

1-Pd and 1-Pt reacted analogously with OH groups on SiO_2–700_ and Al_2_O_3–600_ with the same characteristic bands observed in IR and NMR spectroscopies (Figures S22 and S23).

### Particle Formation

**Figure 4:**
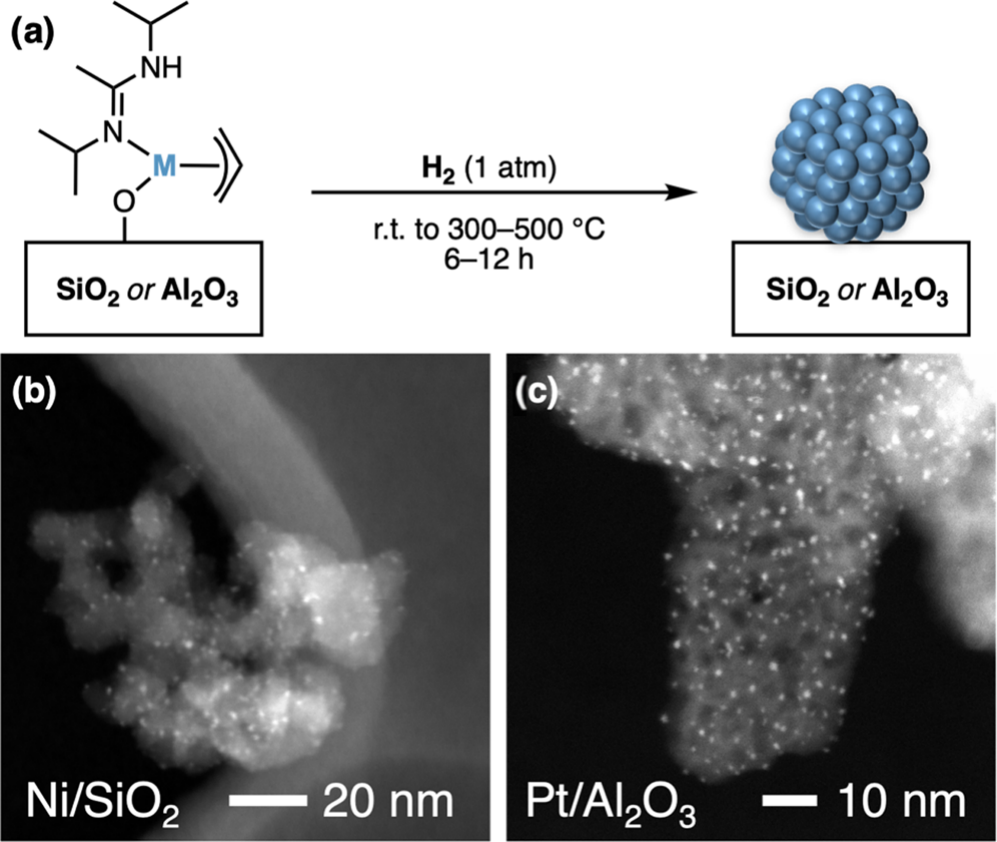
(a) Hydrogen treatment leads to the formation of supported nanoparticles. (b) HAADF-STEM image of Ni/SiO_2_ and (c) Pt/Al_2_O_3_. Other images of M/M’O*_x_* in the SI (Figures S32–S38).

The materials 1-M/SiO_2–700_ (M = Ni, Pd, Pt) were subjected to a temperature treatment under a flow of H_2_ (Figure 4a). From IR spectroscopy, the removal of organic fragments from the surface and the re-emergence of terminal OH groups were observed (Figures S17–S19). HAADF-STEM micrographs reveal that particles form for all materials (Figures 4b and S32–S34). The observed particle sizes are given in Table 2 (and Figure S39).

**Table 2:** Particle Sizes From STEM and H_2_ Chemisorption

material	STEM (nm)	H_2_ chemisorption (nm)
Ni/SiO_2_	1.9 (σ = 0.6)	
Pd/SiO_2_	3.1 (σ = 1.6)	2.1
Pt/SiO_2_	1.8 (σ = 0.5)	1.9
Pd/Al_2_O_3_	1.5 (σ = 0.3)	N.A.
Pt/Al_2_O_3_	1.3 (σ = 0.2)	0.9
PdGa/SiO_2_	1.9 (σ = 0.3)	

For Ni and Pt, the obtained particles were small and size homogeneous. In contrast, Pd/SiO_2_ shows a broader (and in some areas bimodal) particle size distribution (Figure S33). Analogously, the materials 1-M/Al_2_O_3–600_ (M = Ni, Pd, Pt) were subjected to a temperature/hydrogen treatment (Figure 4a). IR spectroscopy again confirms the removal of organic ligands, while the OH groups are partially regenerated (Figures S21–S23). The materials were analyzed using HAADF-STEM imaging. For Pd and Pt, small nanoparticles with a narrow size distribution were obtained (Figures S37 and S38, Table 2, entries 4 and 5). For Ni/Al_2_O_3_, particles could not be resolved well enough by STEM techniques (Figure S36) to allow for determination of size, likely due to low contrast in combination with small particles (≤1 nm). However, the formation of Ni(0) nanoparticles is supported by Ni K-edge X-ray absorption spectroscopy (XAS), which reveals the metallic state of Ni by the overlap of the rising absorption edges of Ni/SiO_2_ and Ni/Al_2_O_3_ with the Ni reference foil in the XANES spectra (Figure S52). This is corroborated by the absence of Ni–O paths and the exclusive presence of the Ni–Ni features in the Fourier transform of the EXAFS region (*R*-space) (Figure S53). EXAFS fitting of Ni/SiO_2_ and Ni/Al_2_O_3_ was performed, revealing the coordination numbers of the first and second shell, which both correspond to Ni–Ni distances. Based on the work of Calvin et al., the particle sizes were estimated from the coordination numbers (Figure S57).^31^ For the SiO_2_-based sample, a mean particle size of 1.9 nm was found, which is in agreement with STEM. For Ni/Al_2_O_3_, the observed coordination number is too low (5.4) to allow for accurate estimation of particle size but is consistent with an average particle size in the sub-nanometer range. This is also supported by the shortening of the Ni–Ni bond lengths (2.44 vs 2.47 Å in bulk Ni) and the low coordination number of the second coordination shell (Figure S53, Table S2).

To further corroborate the particle sizes observed by TEM, Pd and Pt materials were analyzed by H_2_ chemisorption. The estimated average particle sizes are given in Table 2, and the estimated metal dispersions are given in Table S1.^32,33^ For Pt/SiO_2_, the estimation from STEM is well in line with results obtained from H_2_ chemisorption, however, for Pt/Al_2_O_3_, analysis of STEM micrographs slightly overestimates the particle size, which can be rationalized by the poor contrast of small (sub-nanometer) nanoparticles. For the Pd/SiO_2_ material, the average particle sizes obtained from STEM and chemisorption differ by 1 nm, which is in line with a decreased size homogeneity observed by STEM. The Pd/Al_2_O_3_ material could not be analyzed via the same approach due to very high H_2_ adsorption (ca. 1.5 H/Pd), which is, however, qualitatively in line with a smaller average particle size.

CO adsorption IR spectroscopy revealed that the Pd and Pt materials are reduced after the hydrogen treatment, and no bands corresponding to CO bound to oxidized sites were observed.^34^ The reduction of 1-Pd/SiO_2–700_ was further investigated by in situ XAS-TPR, which revealed that a large proportion of Pd is readily reduced in H_2_ at room temperature (Figure S51). However, the final temperature is required to remove all ligand residues from the surface.

### Application in the Synthesis of Bimetallic CO_2_ Hydrogenation Catalysts

**Scheme 2:**
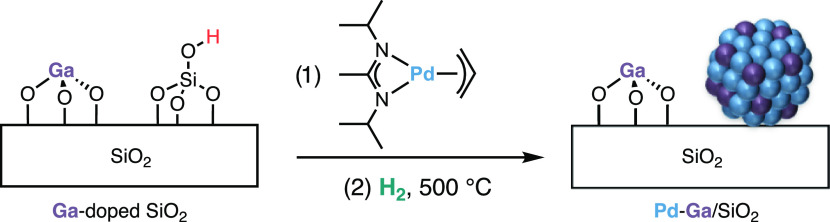
Synthesis of PdGa/SiO_2_ Using 1-Pd

To evaluate the broader applicability of these precursors, we also prepared one bimetallic material, PdGa/SiO_2_, by grafting 1-Pd on a Ga-doped silica support (Scheme 2) because it can yield efficient CO_2_ hydrogenation catalysts.^35,36^ Subsequent hydrogen treatment led to the formation of nanoparticles (1.9(4) nm), while organic fragments were removed from the surface (IR spectrum, Figure S20).

**Figure 5:**
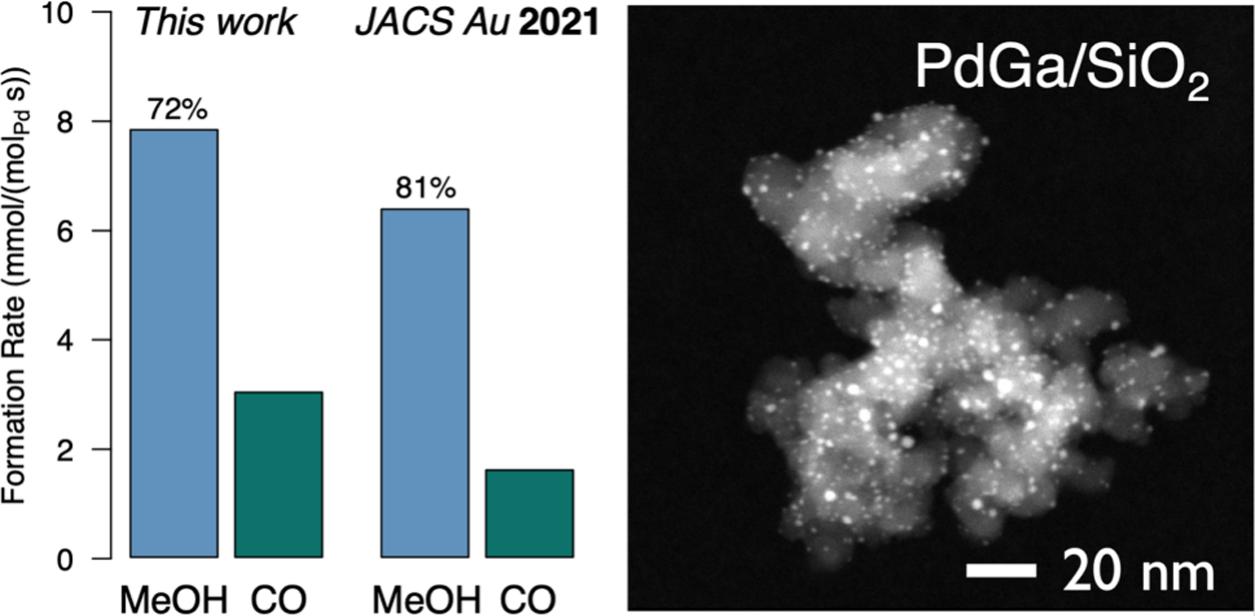
Catalytic performance in CO_2_ hydrogenation in comparison to previously reported SOMC-derived PdGa system and HAADF-STEM micrograph of PdGa/SiO_2_.

This material displays similar yet slightly higher initial formation rate of methanol under CO_2_ hydrogenation conditions (7.9 vs 6.4 mmol/(mol_Pd_ s), Figure 5) than previously reported SOMC-derived PdGa catalysts, albeit with slightly lower intrinsic selectivity toward methanol (72 vs 80%).^36^ Overall, the performance is comparable, and these small differences could arise from deviations in the Pd/Ga ratio and/or nanoparticle size.

In contrast to the monometallic Pd/SiO_2_ material, the CO-IR spectra of PdGa/SiO_2_ (Figure S28) do not show any μ_2_-bridged adsorption sites (observed at 1986 cm^–1^ for Pd/SiO_2_) but only slightly red-shifted terminal CO bands (2052 vs 2094 cm^–1^ for Pd/SiO_2_), indicating that Pd and Ga are alloyed, which is in line with previous reports.^36^

## Conclusions

In summary, herein, we describe the synthesis and application of three isostructural, heteroleptic group 10 complexes of η^3^-allyl and diisopropylamidinate (DIA). The reactivity toward oxide surfaces terminated by hydroxy groups (i.e., SiO_2_ and Al_2_O_3_) was investigated, and the obtained materials were characterized by IR- and ^13^C-MAS SSNMR spectroscopy, which revealed that the grafting occurs via protonation of the amidinate ligand in all cases. Treatment of these materials at elevated temperatures under a flow of H_2_ leads to reduction of the metals forming supported nanoparticles, while organic fragments are removed from the surface. Applying this methodology to a Ga-doped silica support, we demonstrate the synthesis of a bimetallic catalyst material proficient in the hydrogenation of CO_2_ to methanol, replicating the reactivity of an analogous SOMC-derived PdGa/SiO_2_ catalyst. Overall, the presented work introduces a new family of group 10 metal complexes with application in the synthesis of SOMC-derived model systems of complex catalytic materials and related techniques.

In view of the unique chemical and physical properties of these precursor molecules, we are currently exploring their use in other applications, such as colloidal synthesis, atomic layer deposition, and related methodologies.

## Experimental Section

### Synthesis of 1-Ni

Bis(cyclooctadiene)nickel(0) (940 mg, 3.42 mmol, 1.0 equiv) was suspended in pentane (80 mL) and cooled to −40 °C. While stirring, allylchloride (0.65 mL, 8.0 mmol, 2.3 equiv) was added dropwise via a syringe. The mixture was then allowed to warm to room temperature (r.t.) over the course of ca. 1.5 h. The volatiles were removed, which afforded a red solid. LiDIA(thf) (755 mg, 3.43 mmol, 1.0 equiv) was added, followed by pentane (60 mL). The reaction mixture was stirred at r.t. for 20 h. The volatiles were removed, and the residue was extracted with pentane (ca. 15 mL) and filtered over celite, and the filtrate was concentrated affording an orange–red solid. Further purification was achieved by sublimation (40 °C, 10^–3^ mbar, onto a cooling finger at 0 °C) to afford 752 mg of 1-Ni as an orange–red crystalline solid (91% yield). Single crystals suitable for X-ray diffraction were obtained by cooling a concentrated pentane solution to −40 °C for several hours (CCDC deposition number: 2241137).

### Synthesis of 1-Pd

Allylpalladium chloride dimer (706 mg, 1.93 mmol, 1.0 equiv) and LiDIA(thf) (850 mg, 3.86 mmol, 2.0 equiv) were added pentane (60 mL), and the reaction mixture was stirred for 16 h at room temperature. The precipitated LiCl and traces of metallic Pd were removed from the yellow solution by filtration over celite, and the filtrate was concentrated to dryness affording a yellow solid. Further purification was achieved by sublimation (45 °C, 10^–3^ mbar, onto a cooling finger at 0 °C) to give 825 mg of 1-Pd as a yellow crystalline solid (74% yield). Single crystals suitable for X-ray diffraction were obtained by cooling a concentrated pentane solution to −40 °C for several hours (CCDC deposition number: 2241138).

### Synthesis of 1-Pt

Karstedt’s catalyst in vinyl-terminated polysiloxane 3.25 wt % Pt (22.3 g, 3.7 mmol Pt, 1.0 equiv) was diluted with pentane (80 mL). While stirring at r.t., allylchloride (0.6 mL, 7.4 mmol, 2 equiv) was added dropwise via a syringe. Within ca. 1 min, a yellow precipitate formed from the colorless solution. The reaction mixture was stirred for an additional 45 min; the stirring was stopped and the colorless solution was decanted from the yellow precipitate using a cannula fitted with a filter. The residue was washed with pentane (35 mL) and dried under vacuum (10^–3^ mbar), affording a yellow paste (likely {Pt(η^3^-allyl)Cl}_4_ with some residual polysiloxane).^27^ To this reaction mixture was added LiDIA(thf) (819 mg, 3.72 mmol, 1.0 equiv) as a solid, followed by pentane (100 mL) and toluene (25 mL). The resulting suspension was stirred for 4 days, which was accompanied by a gradual change in color to orange–brown and formation of a greyish white precipitate. The mixture was filtered over a plug of celite, and the filtrate was concentrated to dryness, affording a caramel-colored slightly oily residue. The crude product was then purified by sublimation (60 °C, 10^–3^ mbar, onto a cooling finger at 0 °C), affording 1.11 g of 1-Pt as a snow-white crystalline solid (79%). Single crystals suitable for X-ray diffraction were grown by slow evaporation of a pentane solution at −40 °C over several days (CCDC deposition number: 2241139).

### Grafting

Graftings were performed in solution (C_6_H_6_) or via sublimation onto the support. The exact procedures are provided in the SI.

### Particle Formation

The grafted material was transferred to a glass flow reactor containing a medium porosity frit in a glovebox under Ar. The reactor was evacuated (10^–5^ mbar) and filled with H_2_. While maintaining a flow of ca. 10 mL min^–1^ of H_2_, a temperature treatment was applied. While still hot, the reactor was evacuated (10^–5^ mbar) and cooled down under vacuum for 1 h. The reactor was then transferred to a glovebox under Ar, and the material (black powder) was recovered. The exact procedures are provided in the SI.

### General Considerations

Unless noted otherwise, all manipulations were performed under protective Ar atmosphere. All solvents were purified by a solvent purification system (SPS) or by drying followed by distillation and stored over activated molecular sieves. LiDIA(thf), [Mg(CH_2_Ph)_2_(thf)_2_], SiO_2–700_ (Aerosil-200 Degussa/Evonik), and Ga@SiO_2_ were prepared according to published procedures.^37−40^ Al_2_O_3–600_ was prepared from compacted γ-Alumina (PURALOX SBa 200 from SASOL) by calcination in air at 500 °C, followed by vacuum treatment at 10^–5^ mbar and 600 °C for 24 h (heating ramp from 500 to 600 °C: 1 °C/min). IR spectra were recorded inside an Ar-filled glovebox on a Bruker FTIR Alpha spectrometer or on a Nicolet 6700 FTIR spectrophotometer. Solution NMR spectra were recorded on a 300 MHz Bruker DRX spectrometer or on a 500 MHz Bruker Avance II HD spectrometer at room temperature. Spectra were referenced to the residual signals of the deuterated solvent (C_6_D_6_: 7.16 ppm for ^1^H NMR spectra, 128.06 ppm for ^13^C NMR spectra).^41^ Single-crystal X-ray diffraction data for 1-Ni and 1-Pd was collected on a Rigaku XtaLAB Synergy-S diffractometer with a Dualflex HyPix-6000HE detector using Cu Kα radiation. Data for 1-Pt was collected on a Bruker Venture D8 diffractometer equipped with a Photon II detector using Mo Kα radiation. After data collection, structures were solved by intrinsic phasing (SHELXT) and refined by full-matrix least-squares procedures using SHELXL in the Olex2 program suite.^42−44^ X-ray data is available at the CCDC database (CCDC deposition numbers: 1-Ni: 2241137, 1-Pd: 2241138, 1-Pt: 2241139). TGA measurements were performed on an STA 449 F5 Jupiter from Netzsch using an Al_2_O_3_ crucible and lid. Solid-state NMR spectra were recorded on a Bruker 400 MHz spectrometer using a double resonance 3.2 mm CP-MAS probe. Temperature-programmed reduction (TPR) was performed on a BelCat-B catalyst analyzer from Bel Japan. H_2_ chemisorption experiments were carried out on a BELSORP-max apparatus from Bel Japan. Particle sizes were estimated from the obtained H/M ratio.^32,33^ UV–vis spectra were recorded on an Agilent Cary 4000 UV–vis spectrophotometer. DFT geometry optimizations were performed with the Gaussian09 package at the PBE0 level using the GD3 Grimme dispersion correction.^45−47^ Metal atoms were represented by the SDD basis sets with the corresponding effective core potential.^48−50^ Remaining atoms (C, H, N) were represented by the Def2SVP basis sets.^51,52^ Micrographs for particle size determination of the materials were acquired by high-angle annular dark-field scanning transmission electron microscopy (HAADF-STEM) using an FEI Talos F200X, which was operated at 200 kV. CO_2_ hydrogenation reactions were conducted in a fixed-bed tubular reactor with 9.1 mm inner diameter (PID Eng&Tech). For a catalytic test, 170 mg of powdered catalyst was mixed with 5 g of SiC and loaded into the reactor under ambient atmosphere. The loaded catalyst was pretreated under a flow of hydrogen (50 mL min^–1^, atmospheric pressure) at 300 °C for 2 h. Afterward, the reactor was cooled to 230 °C and pressurized to 25 bar with the reaction gas mixture H_2_/CO_2_/Ar (3:1:1, 50 mL min^–1^) for 30 min. The effluent gas phase was analyzed by GC-FID/TCD (Agilent 7890B), injecting every 30 min (1st injection after 30 min) using the FID for CH_3_OH and TCD for CO_2_, CO, CH_4_, and Ar. Flowrates were varied between 6 and 100 mL min^–1^ (STP). XAS measurements at the Ni K-edge and Pd K-edge were performed at the SuperXAS beamline (X10DA) at the Swiss Light Source (SLS, PSI, Villigen, Switzerland).

## Supporting Information Available

Experimental section, characterization data of molecules and materials, and CO_2_ hydrogenation data (PDF)
