# Supplementary material for: Group 10 Metal Allyl Amidinates: A Family of Readily Accessible and Stable Molecular Precursors to Generate Supported Nanoparticles
Source: JACS Au. 2023 Aug 1;3(8):2314–22. doi: 10.1021/jacsau.3c00334 (PMC10466329; doi:10.1021/jacsau.3c00334)
Supplement: Supplementary file 1 — au3c00334_si_001.pdf [file au3c00334_si_001.pdf]

Supporting Information for

Group 10 Metal Allyl Amidinates: A Family of Readily Accessible and Stable Molecular Precursors to Generate Supported Nanoparticles

Christian Ehinger[a], Xiaoyu Zhou[a], Max Candrian[a], Scott R. Docherty[a], Stephan Pollitt[a,b] and Christophe Copéret[a,*]

[a]D-CHAB, ETH Zürich, Vladimir–Prelog-Weg 1–5, 8093 Zürich, Switzerland

[b]PSI, Forschungsstrasse 111, 5232 Villigen, Switzerland

Contents

1. General Considerations **3**

2. Experimental Section **6**

2.1 Synthesis of Molecular Precursors *6*

2.2 Grafting on SiO_{2-700} and Al_2O_{3-600} *8*

2.3 Formation of Nanoparticles *9*

3 NMR Spectra **11**

3.1 Solution NMR Spectra of Molecular Precursors *11*

3.2 MAS Solid State NMR Spectra *16*

1-Ni/SiO_2 16

1-Pd/SiO_2 17

1-Ni/Al_2O_3 17

1-Pd/Al_2O_3 18

4 IR Spectra **19**

4.1 IR Spectra of Molecular Precursors and Materials *19*

4.2 CO-Adsorption IR Spectra *24*

5 Electron Microscopy **27**

6 H_2 Chemisorption **30**

7 Thermogravimetric Analysis (TGA) **32**

8 UV-Vis Spectra **33**

9 Temperature-Programmed Reduction (TPR) **35**

10. X-Ray Absorption Spectroscopy **36**

10.1 In Situ XANES Pd K-Edge *36*

10.2 Ex Situ XAS Ni K-Edge *36*

10.3 Ni Particle Size Estimation from Coordination Number *39*

11 CO_2 Hydrogenation **40**

12 XRD Refinement and Crystal Summary **44**

13 References **46**

1. General Considerations

Unless noted otherwise, all syntheses were performed using standard Schlenk techniques under Ar atmosphere or inside an MBraun glovebox filled with Ar. Benzene and benzene-d_6 were dried over Na/benzophenone and distilled before use, and stored over molecular sieves (3 Å). Pentane, toluene and diethyl ether were dried using an MBraun solvent purification system (alumina columns), then subjected to three freeze-pump-thaw cycles and stored over molecular sieves (3 Å). Allyl chloride was subjected to three freeze-pump-thaw cycles and dried over molecular sieves (3 Å) prior to use. $Ni(COD)_2$ (Strem 98+%), $\{Pd(\eta^3\text{-allyl})Cl\}_2$ (Sigma-Aldrich 98%), and Karstedt's catalyst in vinyl-terminated polysiloxane (ABCR 3.25% Pt) were used as received. SiO_{2-700} (from compacted Degussa Aerosil-200 by Evonik, BET Surface area ca. 200 m^2/g) and Ga@SiO_2 were prepared according to published procedures.[1] Across several batches, OH densities on SiO_{2-700} ranged 0.28–0.30 mmol/g, ~1 OH/nm^2. The thf adduct of lithium diisopropylacetamidinate (LiDIA(thf)) was synthesized according to published procedures.[2,3] The surface OH density was determined by titration using $[Mg(CH_2Ph)_2(THF)_2]$ (prepared according to published procedures[4]) in C_6D_6 and quantification of toluene by ^{1}H-NMR spectroscopy, using ferrocene (sublimed from commercial sources) as internal standard. Celite® and molecular sieves (3 Å) were activated by evacuation at 10^{-5} mbar and 350 °C for ca. 12 h. Deionized water was collected from Merck Millipore Synergy® Water Purification System. Al_2O_{3-600} was prepared from compacted γ-Alumina (PURALOX SBa 200 from SASOL) by calcination in air at 500 °C, then vacuum treatment at 600 °C for 24 h (heating ramp from 500-600 °C: 1 °C/min).

Single Crystal X-Ray Diffraction: The crystals were placed on a microscopy slide inside an Ar-filled glovebox and covered with a drop of perfluoropolyalkylether oil. The microscopy slide was removed from the glovebox and a suitable crystal was picked under a microscope and transferred to the diffractometer (nitrogen stream at 100 K). Data for **1-Ni** and **1-Pd** was collected on a Rigaku XtaLAB Synergy S diffractometer with Dualflex HyPix-6000HE detector using Cu-K_α radiation. Data for **1-Pt** was collected on a Bruker Venture D8 diffractometer equipped with a Photon II detector using Mo-K_α radiation. After data collection, structures were solved by intrinsic phasing (SHELXT) and refined by full-matrix least-squares procedures using SHELXL in the Olex2 program suite.[5–8] Atoms (except H) were refined with anisotropic displacement parameters and H atoms were placed in positions of optimized geometry. For **1-Pd** one (of two) molecule in the unit cell exhibited strong correlated disorder, which was modeled using isotropic displacement parameters. X-Ray data is available at the CCDC database (CCDC deposition numbers: **1-Ni**: 2241137, **1-Pd**: 2241138, **1-Pt**: 2241139).

Solution NMR Spectroscopy: Solution ^{1}H-NMR and ^{13}C-NMR spectra of Li(DIA)(thf), analysis of grafting solutions and SiO_{2-700} titrations were recorded on a 300 MHz Bruker DRX spectrometer at room temperature. Solution ^{1}H- and ^{13}C-NMR, and ^{1}H-^{13}C-HSQC spectra of **1-Ni**, **1-Pd**, and **1-Pt** were measured on a 500 MHz Bruker Avance II HD. Spectra were processed, analyzed, and plotted using the MestreNova software package. Signal multiplicity is reported as s=singlet, d=doublet, t=triplet, hept=heptet, m=multiplet, or combinations thereof. Observed J-couplings (J) are reported in Hz. Spectra were referenced to the residual signals of the deuterated solvent (C_6D_6: 7.16 ppm for ^{1}H NMR spectra, 128.06 ppm for ^{13}C NMR spectra).[9] For quantification of graftings or -OH densities, ferrocene ($FeCp_2$) was used as internal standard, and extended recycle delay (58 s) was used to ensure accurate quantification.

Solid-state MAS NMR Spectroscopy: Solid-state NMR spectra were recorded on a Bruker 400 MHz spectrometer using double resonance 3.2 mm CP-MAS probe. Samples were packed in 3.2 mm

zirconia rotors inside an Ar-filled glovebox, and all spectra were recorded at 298 K. In all cases, the downfield ^{13}C resonance of adamantane (38.5 ppm) was used as an external secondary reference to calibrate chemical shifts.[10,11] In each case, the recycle delay was set at 1.3*(T_1(^{1}H)). T_1(^{1}H) was measured using a saturation recovery experiment. For all experiments, N_2 was used to provide both bearing and drive pressure. The MAS frequency was set to 16 kHz. Where spinning sidebands appear, they are denoted with an asterisk.

TPR: Temperature-programmed reduction (TPR) was performed on a BelCat-B catalyst analyzer from Bel Japan. Immobilized molecular precursors (M(η^3-allyl)(DIA)/$SiO_{2\text{-}700}$) were charged into a U-shaped quartz cell, which was attached to an adapter using ultra torr fittings equipped with a bypass loop (Swagelok® 3-way valve) to allow for air-free loading of samples, as well as a thermocouple. In a normal experiment, ca. 100 mg of sample were added to the cell and assembled inside an Ar-filled glovebox. The cell was mounted via Swagelok® quick-connects and the bypass was flushed with 50 sccm Ar for ca 10 min. Subsequently, the gas flow was directed to the sample cell, starting the TPR experiment. The samples were heated with a ramp of 5 °C/min from ambient temperature to 725 °C under 30 sccm of H_2/He (1:5). Changes in the gas composition during TPR experiments were tracked via a thermal conductivity detector (W-Re filament).

UV-Vis Spectroscopy: UV-Vis spectra were recorded on an Agilent Cary 4000 UV-Vis Spectrophotometer. Samples were prepared inside an Ar-filled glovebox as a ca. 0.1 mM solution in pentane and transferred to a custom-made quartz cuvette of 1 cm path length with a J-Young cap to prevent air exposure.

Elemental analysis: EA of C, H, N were carried out in the Mikrolabor of ETH Zürich on a LECO TruSpec® Micro spectrometer. EA of Ni, Pd, Pt, Ga was performed by Mikroanalytisches Labor Pascher (an der Pulvermühle 1, D-53424 Remagen, Germany) using ICP-AES.

Computational Details: Geometry optimizations were performed with the Gaussian09 package at the PBE0 level, using the GD3 Grimme dispersion correction.[12–14] Metal atoms were represented by the SDD basis sets with corresponding effective core potential.[15–17] Remaining atoms (C, H, N) were represented by the Def2SVP basis sets.[18,19]

IR Spectroscopy: IR spectra were recorded inside an Ar-filled glovebox on a Bruker FT-IR Alpha spectrometer. Molecular complexes were measured using ATR-IR, silica- and alumina-based materials were measured in transmission mode. Stacked spectra of silica-based materials (Figures S17–S20 and S24–S27) were normalized with respect to the Si-O-Si overtone band maximum at 1868 cm^{-1}. Alumina based materials spectra were adjusted in intensity by hand to allow for easier comparison.

For adsorption experiments (CO), spectra were recorded in transmission mode on a Nicolet 6700 FTIR spectrophotometer, equipped with an uncooled deuterated tryglycine sulfate (DTGS)KBr detector, using 32 scans at a resolution of 2 cm^{-1}. For adsorption experiments, a wafer of catalyst material (0.01- 0.02 g cm^{-1}) was pressed into an aluminum ring, which was placed into a glass IR cell with CaF_2 windows. For IR spectra with adsorbed CO, the evacuated cell was exposed to different pressures incrementally (typically ca. 2.5, 10, 50 mbar) at 298 K and a spectrum was recorded after each dosage. Finally, the cell was evacuated for ca. 10 minutes and another spectrum was recorded.

Electron Microscopy: Micrographs of the materials were acquired by high angle annular dark-field scanning transmission electron microscopy (HAADF-STEM). The instrument used was a FEI Talos F200X, which was operated at 200 KV. Pd and Pt containing samples were prepared by placing a powder on a Lacey-C 400 mesh Cu grid under air, Ni containing and PdGa/SiO_2 samples were prepared by placing a powder on a Lacey-C 400 mesh Cu grid in a glovebox under Ar atmosphere, then transferring the grid to the microscope using a vacuum transfer tomography holder from

Fischione Instruments (model #2560). This ensured that the samples were transferred into the microscope without exposure to air. To evaluate particle size, particles were measured manually from the micrographs. Approximately 200 particles were measured for each sample.

Catalytic tests (CO_2 Hydrogenation): CO_2 hydrogenation reaction were conducted in a fixed-bed tubular reactor with 9.1 mm inner diameter. (PID Eng&Tech) For a catalytic test, 170 mg of powdered catalyst was mixed with 5 g of SiC and loaded into the reactor under ambient atmosphere. The loaded catalyst was pretreated under flow of hydrogen (50 ml min^{-1}, atmospheric pressure) at 300 °C for 2h. Afterwards, the reactor was cooled to 230 °C and pressurized to 25 bar with the reaction gas mixture H_2:CO_2:N_2 (3:1:1, 50 ml min^{-1}) for 30 min. The effluent gas phase was analyzed by GC-FID/TCD (Agilent 7890B), injecting every 30 minutes (1st injection after 30 minutes) using FID for CH_3OH and TCD for CO_2, CO, CH_4 and N_2. Flowrates were varied between 6 – 100 ml min^{-1} (STP) to probe the effect of different contact times. Every third flowrate restored initial conditions (50 mL min^{-1}) to track and account for potential deactivation of the catalyst. Each flowrate was sampled 6 times by GC (total of 3 hours), of which only the data of the last three injections (stabilization to a steady state) were considered for the analysis. Intrinsic formation rates are obtained via exponential fit (rate(1/GHSV) = $a \cdot e^{-b\cdot\left(\frac{1}{GHSV}\right)} + c$, fit parameters a, b, c) on the experimental data and extrapolating to zero contact time (1/GHSV = 0, rate(0) = $a + c$).

In Situ XANES: *In situ* X-ray absorption spectra (XAS) at the Pd K edge were measured at the SuperXAS beamline (X10DA) at the Swiss Light Source (SLS, PSI, Villigen, Switzerland), operating in top-up mode at a 2.4-GeV electron energy and a current of 400 mA. Calibration of the Pd K edge, a Pd foil (24350 eV) was used as inline reference, placed between the second and third ion chambers. The incident photon beam was selected by a liquid nitrogen cooled Si (111) quick-EXAFS monochromator and the rejection of higher harmonics and focusing were achieved by a rhodium-coated collimating mirror. The beam size on the sample was approximately 2000 x 500 μm. During measurement, the quick XAS monochromator was rotating with 1 Hz frequency in 2.5 deg angular range and X-ray absorption spectra were collected in transmission mode using ionization chambers specially developed for quick data collection.

During XAS-TPR, approximately 20 mg of the powder sample (250-420 μm) was packed into a 3 mm quartz capillary (i.d. 2.8 mm, bed length ca. 1 cm) which was integrated to a pressurizable gas flow system consisting of 2 parallel arrays, each consisting of 3 mass flow controllers (Bronkhorst), while the total pressure was maintained by a back-pressure regulator (Bronkhorst EL-Press). Switching between the two systems (i.e. switching the MFC array that was feeding the capillary) was performed using a remote-controlled 6-port 2-way switching valve (VICI, Valco) that could be operated from outside the experimental hutch. While one gas mixture was flowing to the cell, the other was directed *via* a bypass to the exhaust. Samples were heated using a custom-built infrared heater (Elstein-Werk M. Steinmetz GmbH & Co. KG (Germany), 30 mm length, with two heating elements – one above and one below sample capillary), and temperature was monitored/maintained using a 0.3-mm K-type thermocouple placed in direct contact with the material. Ar and H_2 were purified by passing through a trap containing molecular sieves and Q5 catalyst prior to introduction to the XAS cell. In the *in situ* experiment, Ar (10 sccm, 1 bar) was flowed over the grafted material while the spectra were recorded for 15 minutes. The gas flow was then changed to Ar/H_2 (4:1, 10 sccm, 1 bar), and the sample was kept at room temperature for 45 minutes while recording continuously. Subsequently, the temperature was increased to 500 °C, while maintaining the same flow and gas composition, (ramp 5 °C min^{-1}), while spectra were recorded continuously.

Ex Situ XAS: XAS spectra of the Ni K-edge were collected at the SuperXAS beamline at PSI, which operated in top-up mode at 2.4 GeV and a ring current of 400 mA. A silicon coated mirror (which also served to reduce higher order harmonics) was used to collimate the polychromatic X-rays from a 2.9 T superbend magnet, which were subsequently monochromatized by a Si(111) channel-cut

crystal. The monochromator was rocked at a frequency of 1 Hz resulting in two spectra per second. The beam was focused by a Rh-coated toroidal mirror. The Ni K-edge absorption spectra were collected in transmission mode using 15 cm long ionization chambers filled with 2 bar of N_2 and by measuring a Ni foil simultaneously for absolute energy calibration. Standard background subtraction, interpolation and averaging were done with the python-based software ProQEXAFS.[20] XANES analysis and fitting of the EXAFS region was performed with Larch.[21] The supported Ni nanoparticles were measured in closed capillaries without air exposure. The NiO reference was measured in pellet form. The amplitude reduction factor (S_0^2) was derived from a foil fit with fcc Nickel structure by fixing the coordination number.[22] All fits were performed with a K-range from 3.0 to 10.3 $Å^{-1}$. The fits for the nanoparticle samples were performed with a fixed amplitude reduction factor of 0.74 in the range from 1 to 3.6 Å in R-space with two paths (starting values: 2.49 Å and 3.52 Å). In the fitting strategy an equal change in R and σ^2 was assumed and the coordination numbers were constrained to be positive. Particle size determination was performed based on Calvin et al.[23] The formula and graph are shown in Figure S48.

Thermogravimetric Analysis: TGA Measurements were performed on a STA 449 F5 Jupiter from Netzsch using an Al_2O_3 crucible and lid (6.8 mm diameter, 85 µL). About 5 mg of sample was weighed in a crucible inside a glovebox and transported to the instrument in a teflon-lined screw-cap vial. The crucible was then transferred using the autosampler into a flow of Ar (20 mL/min). A temperature program of 40–500 °C with a ramp of 10 °C/min was applied during the measurement.

H_2 Chemisorption: Chemisorption experiments were carried out on a BELSORP-max apparatus (Bel-Japan). Approximately 100 mg of reduced catalyst was loaded in an airtight cell inside a glovebox which was then mounted on the apparatus. The sample was pre-treated at 300 °C for 1 h at 10^{-5} mbar. The chemisorption measurements were performed at 25 °C, the pressures at equilibrium were recorded when the pressure variation was below 0.6% for 1200 seconds. The molar uptake was calculated from measured volumetric uptake assuming ideal gas law at standard temperature and pressure. The adsorption data is plotted showing the uptake of H_2 per mass of material (Q_{H_2} in µmol/g) as a function of H_2 pressure $\left({}^{P_{H_2}}\!/_{P_0}\right)$. The uptake was fitted using a dissociative Langmuir model

$$Q_{H_2} = \frac{\sqrt{K_{H_2}\left({}^{P_{H_2}}\!/_{P_0}\right)}}{1+\sqrt{K_{H_2}\left({}^{P_{H_2}}\!/_{P_0}\right)}} \cdot Q_{H_2,max},$$

where K_{H_2} is the fit parameter corresponding to the equilibrium constant of adsorbed hydrides and gaseous dihydrogen, and $Q_{H_2,max}$ is the fit parameter corresponding to the maximum H_2 uptake (µmol/g). $Q_{H_2,max}$ can be converted to the H/M (hydrogen atoms per metal) ratio, taking into account the metal loading obtained from elemental analysis. The H/M stoichiometry was then used to estimate the dispersion and average particle sizes.[24,25]

2. Experimental Section

2.1 Synthesis of Molecular Precursors

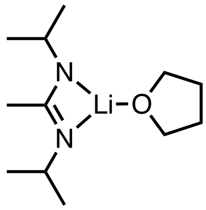

Li(DIA)(THF) (Synthesis adapted from published procedures)[2,3]: Diisopropyl-carbodiimid (3.95 g, 31.3 mmol, 1.05 equiv) was added to a flame dried Schlenk flask under Ar, which was then purged with Ar for about 1 minute. Et_2O (50 mL) was added via canula, and the solution was cooled to -40 °C using an acetonitrile/dry ice bath. A 1.73 M solution of MeLi in Et_2O (17.2 mL, 29.8 mmol, 1.00 equiv) was added dropwise while stirring. The mixture was then allowed to reach room temperature and stirred for 2 hours. The volatiles were removed in vacuo to yield Li(DIA) as a white powder. The crude product was recrystallized by redissolving it in THF (ca. 10 mL),

layering this solution with pentane (ca. 5 mL) and cooling to -40 °C for several hours. Successive crystallization from the mother liquor yielded the THF adduct of Li(DIA) as large, colorless crystalline blocks. Combined yield of three crops: 4.8 g (73%). ^{1}H NMR (300 MHz, C_6D_6): δ 3.58 (m, J = 6.2 Hz, 2H), 3.51 (t, J = 6.4 Hz, 4H), 1.86 (s, 3H), 1.37 (m, J = 3.2 Hz, 4H), 1.27 (d, J = 6.2 Hz, 12H). ^{13}C NMR (75.6 MHz, C_6D_6): δ 170.0, 67.7, 47.7, 26.6, 25.6, 11.9.

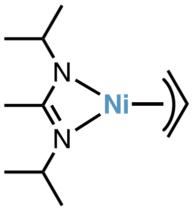

Ni(η^3-allyl)(DIA) (**1-Ni**): Ni(COD)$_2$ (940 mg, 3.42 mmol, 1.0 equiv) was weighed in a Schlenk flask, suspended in pentane (80 mL) and the mixture was cooled to -40°C. Allylchloride (0.65 mL, 8.0 mmol, 2.3 equiv) was added dropwise via syringe. The mixture was then allowed to warm to room temperature, resulting in the gradual dissolution of starting material and a change in color of the mixture to deep red over the course of ca. 1.5 h. The volatiles were removed which afforded a red solid. LiDIA(thf) (755 mg, 3.43 mmol, 1.0 equiv) was added as a solid followed by pentane (60 mL). The reaction mixture was stirred at r.t. for 20 h. The reaction mixture was concentrated to dryness, and the residue was extracted with pentane (ca. 15 mL), filtered over celite and the filtrate concentrated affording an orange-red solid. Further purification was achieved by sublimation (40 °C, 10^{-3} mbar, onto a cooling finger at 0 °C) to afford **1-Ni** as orange-red crystalline solid (752.2 mg, 91% yield over all steps). Single crystals suitable for X-Ray diffraction were obtained by cooling a concentrated pentane solution to -40 °C for several hours (CCDC deposition number: 2241137).
^{1}H NMR (500 MHz, C_6D_6) δ 4.84 (tt, J = 13.1, 7.1 Hz, 1H), 3.12 (hept, J = 6.2 Hz, 2H), 2.88 (d, J = 7.0 Hz, 2H), 1.66 (d, J = 13.1 Hz, 3H), 1.39 (s, 4H), 1.04 (d, J = 6.3 Hz, 7H), 0.84 (d, J = 6.2 Hz, 8H). ^{13}C NMR (126 MHz, C_6D_6) δ 174.5, 104.0, 45.7, 45.0, 25.8, 25.5, 10.1.
EA calcd. for $C_{11}H_{22}N_2Ni$: C 54.82%, H 9.20%, N 11.62%; found: C 54.54%, H 9.13%, N 11.47%.

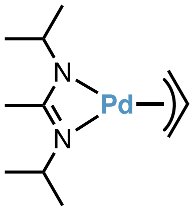

Pd(η^3-allyl)(DIA) (**1-Pd**): In an Ar filled glovebox, allylpalladium chloride dimer (706 mg, 1.93 mmol, 1.0 equiv) and LiDIA(thf) (850 mg, 3.86 mmol, 2.0 equiv) were weighed in Schlenk flask. Pentane (60 mL) was added, and the reaction mixture was stirred for 16 hours at room temperature. The precipitated LiCl and traces of metallic Pd were removed from the yellow solution by filtration over celite, and the filtrate was concentrated to dryness yielding a yellow solid. Further purification was achieved by sublimation of the crude material (45 °C, 10^{-3} mbar, onto a cooling finger at 0 °C) to give **1-Pd** as yellow crystalline solid (825 mg, 74% yield). Single crystals suitable for X-Ray diffraction were obtained by cooling a concentrated pentane solution to -40 °C for several hours (CCDC deposition number: 2241138).
^{1}H NMR (500 MHz, C_6D_6) δ 4.52 (tt, J = 12.3, 6.9 Hz, 1H), 3.54 (d, J = 6.9 Hz, 3H), 3.35 (hept, J = 6.1 Hz, 2H), 2.28 (d, J = 12.3 Hz, 3H), 1.58 (s, 4H), 1.10 (d, J = 6.2 Hz, 8H), 0.97 (d, J = 6.1 Hz, 9H). ^{13}C NMR (126 MHz, C_6D_6) δ 175.7, 106.9, 50.3, 45.8, 26.4, 26.2, 11.1.
EA calcd. For $C_{11}H_{22}N_2Pd$: C 45.76%, H 7.68%, N 9.70%; found: C 45.81%, H 7.62%, N 9.64%.

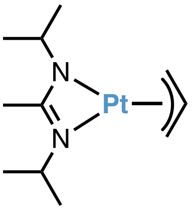

Pt(η^3-allyl)(DIA) (**1-Pt**): Karstedt's catalyst in vinyl-terminated polysiloxane 3.25 wt% Pt (22.3 g, 3.7 mmol Pt, 1.0 equiv) was transferred to a Schlenk flask and diluted with pentane (80 mL). While stirring at r.t., allylchloride (0.6 mL, 7.4 mmol, 2 equiv) was added dropwise via syringe. Within 1 minute, a yellow precipitate formed from the colorless solution. The reaction mixture was stirred for an additional 45 minutes; the stirring was stopped and the colorless solution was decanted from the yellow precipitate. The residue was washed with pentane (35 mL) and dried under vacuum (10^{-3} mbar) affording a yellow paste (likely {Pt(η^3-allyl)Cl}$_4$ with some residual polysiloxane). To this reaction mixture was added LiDIA(thf) (819 mg, 3.72 mmol, 1.0 equiv) as a solid, followed by pentane (100 mL) and toluene (25 mL). The resulting suspension was stirred for 4 days which was accompanied by a gradual change in color to orange-brown and formation of a greyish-white precipitate. The mixture was filtered over a plug of celite and the filtrate was concentrated to dryness affording a caramel-colored slightly oily residue (at this stage, thorough

removal of pentane is important to avoid splashing during sublimation). The crude product was then purified by sublimation (60 °C, 10^{-3} mbar, onto a cooling finger at 0 °C) affording **1-Pt** as a snow-white crystalline solid (1.11 g, 79% yield over all steps). Single crystals suitable for X-Ray diffraction were grown by slow evaporation of a pentane solution at -40 °C over several days (CCDC deposition number: 2241139).
^{1}H NMR (500 MHz, C_6D_6) δ 3.69 – 3.57 (m, 1H), 3.36 (hept, J = 6.2 Hz, and heptd, $^3J_{H-H}$ = 6.2 Hz, $^3J_{Pt-H}$ = 42 Hz, ca. 2:1 ratio, 2H), 1.94 – 1.72 (m, 2H), 1.40 (s, 3H), 1.10 (d, J = 6.2 Hz, 6H), 0.98 (d, J = 6.2 Hz, 6H). ^{13}C NMR (126 MHz, C_6D_6) δ 176.9 ($^2J_{Pt-C}$ = 109 Hz), 89.4 ($^1J_{Pt-C}$ = 72 Hz), 45.5 ($^2J_{Pt-C}$ = 56 Hz), 35.2 ($^1J_{Pt-C}$ = 233 Hz), 25.6 ($^3J_{Pt-C}$ = 18 Hz), 25.5 ($^3J_{Pt-C}$ = 18 Hz), 12.9 ($^3J_{Pt-C}$ = 45 Hz). ^{195}Pt NMR (107 MHz, C_6D_6) δ -4435.2. We observed strong secondary J-coupling of ^{195}Pt (natural abundance 33.8%, I = ½) to the protons on the allyl ligand; the coupling was resolved only for the C–H of the *iso*-propyl group. In the ^{13}C NMR spectrum, all couplings were resolved and observed as weaker doublets (Figure S8).
EA calcd. for $C_{11}H_{22}N_2Pt$: C 35.01%, H 5.88%, N 7.42%; found: C 35.13%, H 5.96%, N 7.46%.

2.2 Grafting on SiO_{2-700} and Al_2O_{3-600}

Grafting of 1-Ni on SiO_{2-700}: SiO_{2-700} (400 mg) was transferred to a Schlenk flask and suspended in C_6H_6 (ca. 5 mL). **1-Ni** (24.1 mg, 0.10 mmol, ca. 0.85 equiv with respect to isolated OH groups) was weighed in a separate container and dissolved in C_6H_6 (ca. 5 mL). The solution of **1-Ni** was then added dropwise to the suspension of SiO_{2-700} while stirring at 100 rpm. A fast discoloration of the solution was observed while the material became orange. Stirring was continued for 1 h, after which the liquid phase was decanted and the material was washed with benzene (3 x 5 mL) and *n*-pentane (2 x 5 mL). The combined benzene washings were added ferrocene (15 mg, internal standard) and 1 mL of C_6D_6 was added to the solution. An aliquot of ca. 1 mL was then analyzed using ^{1}H NMR spectroscopy which showed that no precursor or protonated ligand was remaining in solution. The material was dried (10^{-3} mbar for 30 min, then 10^{-5} mbar for 1 hour) affording an orange powder.
EA: C 3.24%, H 0.58%, N 0.92%, Ni 1.32%.

Grafting of 1-Pd on SiO_{2-700}: Following the same procedure as outlined above with SiO_{2-700} (403 mg) and **1-Pd** (29.0 mg, 0.101 mmol, ca. 0.85 equiv with respect to isolated OH groups) for ca. 2h at r.t. Analysis of the washings by ^{1}H NMR spectroscopy did not show residual **1-Pd** or any ligand being released. A beige to very light-yellow powder was obtained after drying (10^{-3} mbar for 30 min, then 10^{-5} mbar for 1 h).
EA: C 3.23%, H 0.62%, N 0.79%, Pd 2.39%.

Grafting of 1-Pt on SiO_{2-700}: Following the same procedure as outlined above with SiO_{2-700} (435 mg) and **1-Pt** (41 mg, 0.11 mmol, ca. 0.85 equiv with respect to isolated OH groups) for 3 h at 10 °C. Washings were done with precooled (10 °C) solvents. Analysis of the washings by ^{1}H NMR spectroscopy showed ca. 10% residual **1-Pt** and no ligand being released. (Performing the grafting step at room temperature decreased the grafting efficiency to ca. 70%). A beige powder was obtained after drying (10^{-3} mbar for 30 min, then 10^{-5} mbar for 1 h).
EA: C 2.75%, H 0.55%, N 0.72%, Pt 3.58%.

Grafting of 1-Pd on Ga@SiO_2: Following the same procedure as outlined above using Ga@SiO_2 (400 mg) in place SiO_{2-700} and **1-Pd** (19.6 mg, 0.068 mmol) with a grafting time of ca. 2h at r.t. Analysis of the washings by ^{1}H NMR spectroscopy did not show residual **1-Pd** or any ligand being released. A pale-yellow powder was obtained after drying (10^{-3} mbar for 30 min, then 10^{-5} mbar for 1 h).

Grafting of 1-Ni on Al_2O_{3-600}: Following the same procedure as outlined above using Al_2O_{3-600} (401 mg) instead of SiO_{2-700} and **1-Ni** (24.4 mg, 0.101 mmol) with a grafting time of ca. 1 h at r.t.

Analysis of the washings by ^{1}H NMR spectroscopy did not show residual **1-Ni** or any ligand being released. An orange powder was obtained after drying (10^{-3} mbar for 30 min, then 10^{-5} mbar for 1 h).
EA: C 1.81%, H 0.33%, N 0.45%, Ni 0.75%.

Grafting of 1-Pd on Al_2O_{3-600}: Following the same procedure as outlined above using Al_2O_{3-600} (401 mg) instead SiO_{2-700} and **1-Pd** (28.9 mg, 0.10 mmol) with a grafting time of ca. 1 h at r.t. Analysis of the washings by ^{1}H NMR spectroscopy did not show residual **1-Pd** or any ligand being released. A pale-yellow powder was obtained after drying (10^{-3} mbar for 30 min, then 10^{-5} mbar for 1 h).
EA: C 3.32%, H 0.62%, N 0.73%, Pd 2.39%.

Grafting of 1-Pt on Al_2O_{3-600}: Following the same procedure as outlined above using Al_2O_{3-600} (408 mg) instead SiO_{2-700} and **1-Pt** (38.4 mg, 0.102 mmol) with a grafting time of ca. 3 h at 10 °C. Analysis of the washings by ^{1}H NMR spectroscopy did not show residual **1-Pt** or any ligand being released. A beige to off-white powder was obtained after drying (10^{-3} mbar for 30 min, then 10^{-5} mbar for 1 h).
EA: C 3.12%, H 0.58%, N 0.70%, Pt 4.14%.

Grafting of 1-Ni on SiO_{2-700} via sublimation: **1-Ni** (35.8 mg 0.148 mmol, ca. 1.4 equiv with respect to silanol groups on SiO_{2-700}) was vacuum transferred (25 °C, 10^{-5} mbar) to a Schlenk flask that was cooled using liquid N_2 containing SiO_{2-700} (350 mg). The flask was kept under static vacuum (10^{-5} mbar), for 5 h while stirring, during which **1-Ni** – initially deposited on the walls of the flask – migrated to the surface of the material (as evidenced by increased coloration). To speed up the deposition, the lower part of the flask was cooled with liquid nitrogen, and the upper part of the flask was gently warmed with a heat gun (not above 50 °C), in time intervals of about 1 h. After all **1-Ni** was deposited on the surface (grafted + physisorbed excess), the excess 1-Ni was removed under vacuum (10^{-5} mbar, 12 h). The IR spectrum exhibited the same features as observed for the material obtained from grafting in solution.

Grafting of 1-Pd on SiO_{2-700} via sublimation: SiO_{2-700} (497 mg) and **1-Pd** (43.0 mg, 0.149 mmol, ca. 1.0 equiv with respect to silanol groups) were transferred to a Schlenk flask equipped with a stir bar. The flask was cooled with liquid nitrogen, and evacuated for 5 min (10^{-6} mbar). The flask was isolated and allowed to warm to room temperature, then kept at room temperature for 3 h while stirring at 100 rpm. Then, the flask was evacuated at room temperature for 12 h to remove excess **1-Pd**. The IR spectrum exhibited the same features as observed for the material obtained from grafting in solution.

Grafting of 1-Pt on SiO_{2-700} via sublimation: SiO_{2-700} (489 mg) and **1-Pt** (56.1 mg, 0.149 mmol, ca. 1.0 equiv with respect to silanol groups) were transferred to a Schlenk flask equipped with a stir bar. The flask was cooled with liquid nitrogen, and evacuated for 5 min (10^{-6} mbar). The flask was isolated and allowed to warm to room temperature, then kept at room temperature for 3 h while stirring at 100 rpm. Then, the flask was evacuated at room temperature for 12 h to remove excess **1-Pt**. The IR spectrum exhibited the same features as observed for the material obtained from grafting in solution.

2.3 Formation of Nanoparticles

Hydrogen treatment – general procedure: The grafted material was transferred to a glass flow reactor containing a medium porosity frit in a glovebox under Ar. The reactor was evacuated (10^{-5} mbar) and filled with H_2. While maintaining a flow of ca. 10 mL/min of H_2, the reactor was heated (rate=R) to final temperature T, which was held for t hours. While still hot, the reactor was evacuated (10^{-5} mbar), and cooled down under vacuum for 1 hour. The reactor was then transferred to a glovebox under Ar, and the material (black powder) was recovered.

Ni/SiO_2: According to the procedure outlined above from 1-Ni/SiO_{2-700} using a heating ramp R = 1 °C/min, final treatment temperature T = 500 °C, and holding time of t = 12 hours.
Elemental analysis: Ni 1.36%, C 0.5%, N<0.2%.

Pd/SiO_2: According to the procedure outlined above from 1-Pd/SiO_{2-700} using a heating ramp R = 1 °C/min, final treatment temperature T = 400 °C, and holding time of t = 6 hours.
Elemental analysis: Pd 2.59%, C 0.37%, N <0.2%.

Pt/SiO_2: According to the procedure outlined above from 1-Pt/SiO_{2-700} using a heating ramp R = 5 °C/min, final treatment temperature T = 300 °C, and holding time of t = 12 hours.
Elemental analysis: Pt 3.98%, C 0.42%, N <0.2%.

$PdGa/SiO_2$: According to the procedure outlined above from 1-Pd/Ga@SiO_2 using a heating ramp R = 1 °C/min, final treatment temperature T = 500 °C, and holding time of t = 5 hours.
Elemental analysis: Pd 1.76%, Ga 1.29%.

Ni/Al_2O_3: According to the procedure outlined above from 1-Ni/Al_2O_{3-600} using a heating ramp R = 1 °C/min, final treatment temperature T = 400 °C, and holding time of t = 6 hours.
Elemental analysis: Ni 1.31%, C 0.27%, N <0.2%.

Pd/Al_2O_3: According to the procedure outlined above from 1-Pd/Al_2O_{3-600} using a heating ramp R = 1 °C/min, final treatment temperature T = 400 °C, and holding time of t = 6 hours.
Elemental analysis: Pd 2.30%, C 0.35%, N <0.2%.

Pt/Al_2O_3: According to the procedure outlined above from 1-Pt/Al_2O_{3-600} using a heating ramp R = 1 °C/min, final treatment temperature T = 400 °C, and holding time of t = 6 hours.
Elemental analysis: Pt 4.27%, C 0.30%, N <0.2%.

3 NMR Spectra

3.1 Solution NMR Spectra of Molecular Precursors

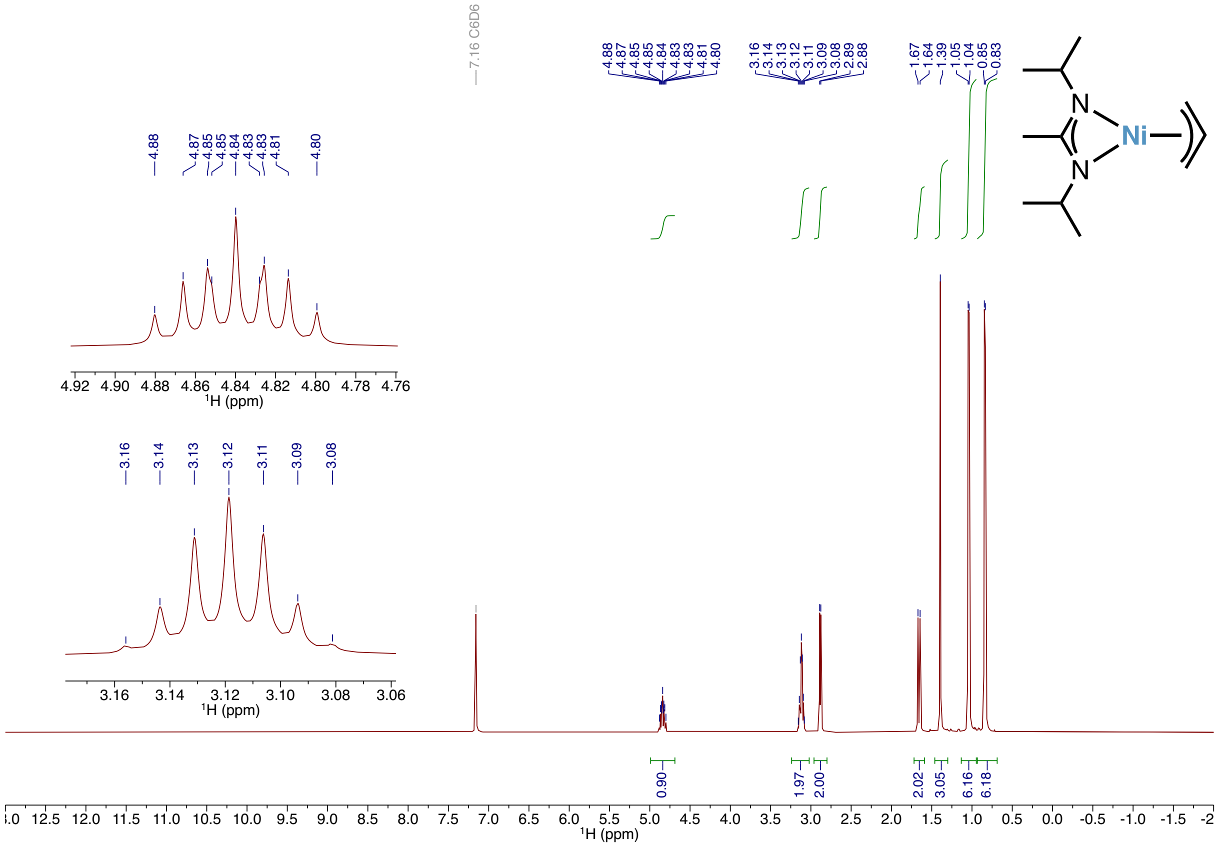

Figure S1. ^{1}H NMR spectrum of **1-Ni** in C_6D_6 at room temperature.

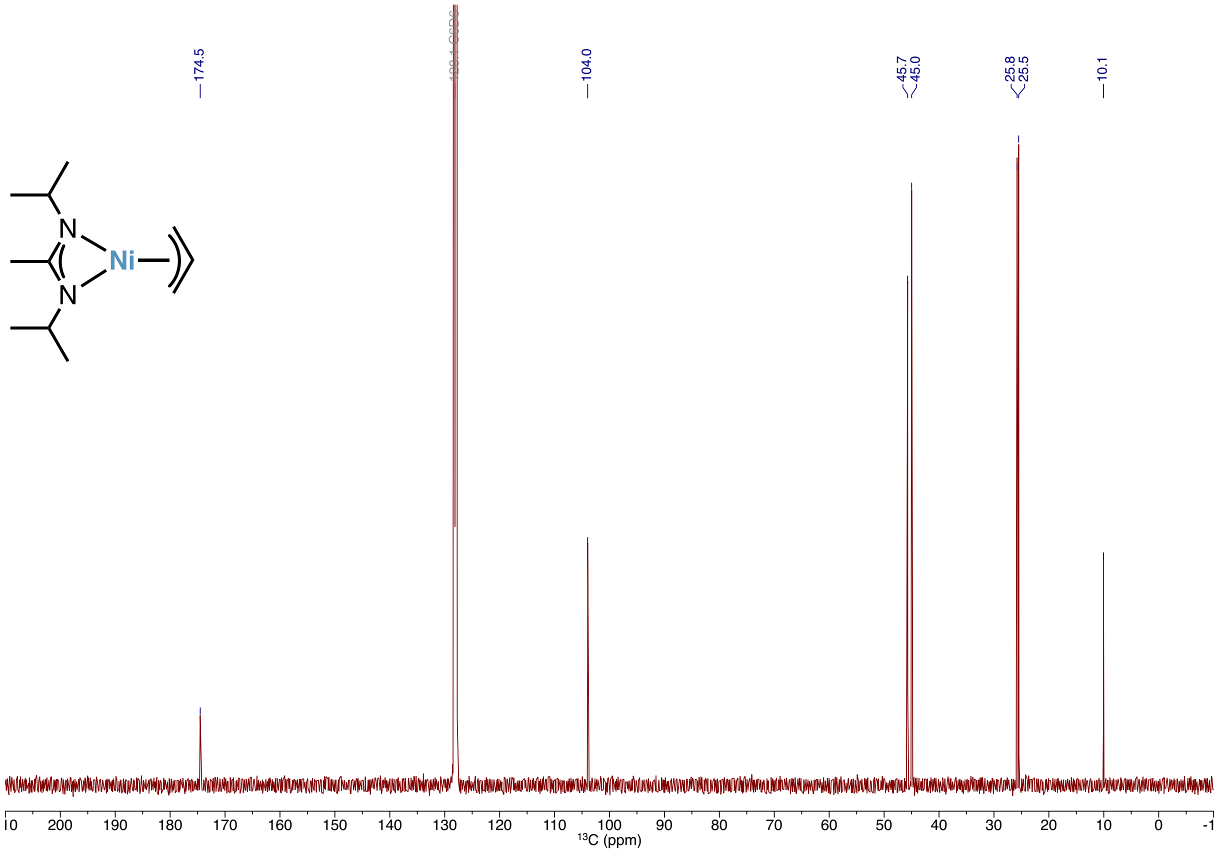

Figure S2. ^{13}C NMR spectrum of **1-Ni** in C_6D_6 at room temperature.

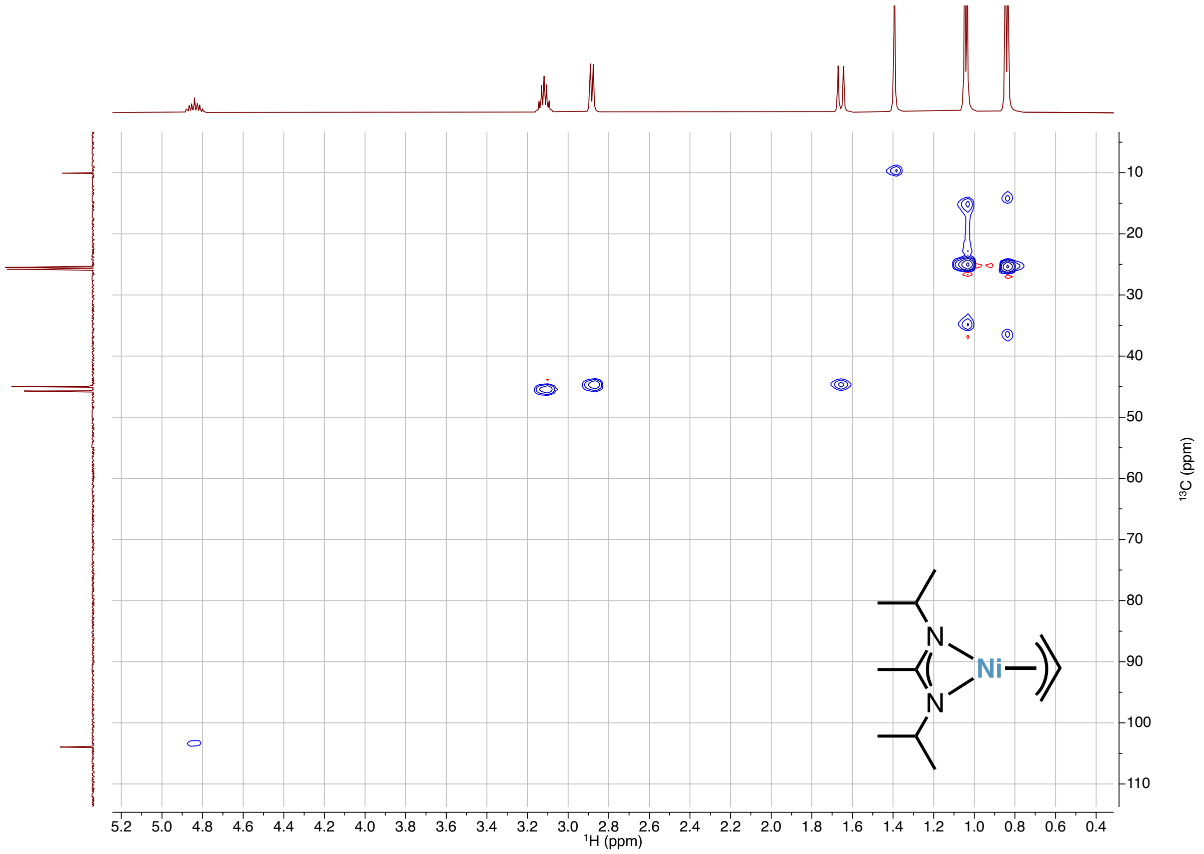

Figure S3. ^{1}H-^{13}C HSQC 2D NMR spectrum of **1-Ni** in C_6D_6 at room temperature.

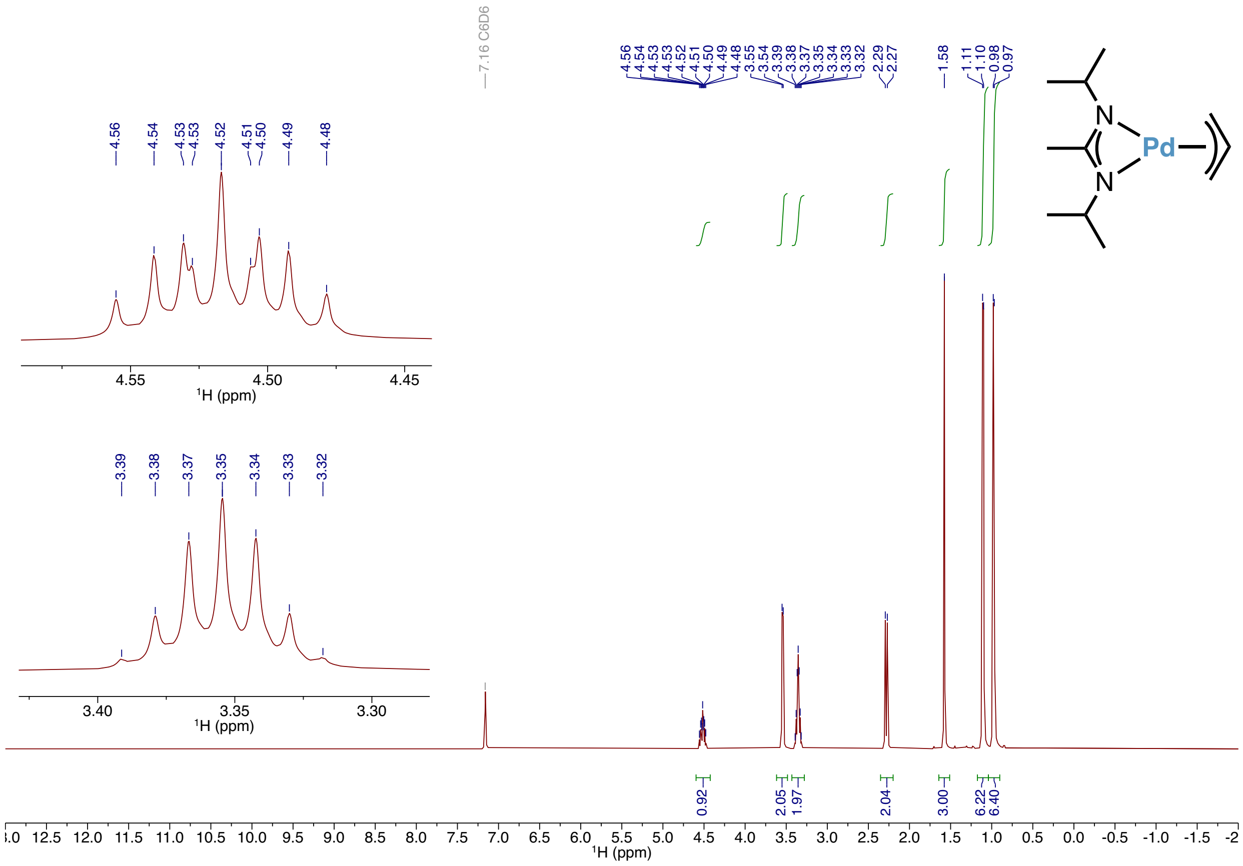

Figure S4. ^{1}H NMR spectrum of **1-Pd** in C_6D_6 at room temperature.

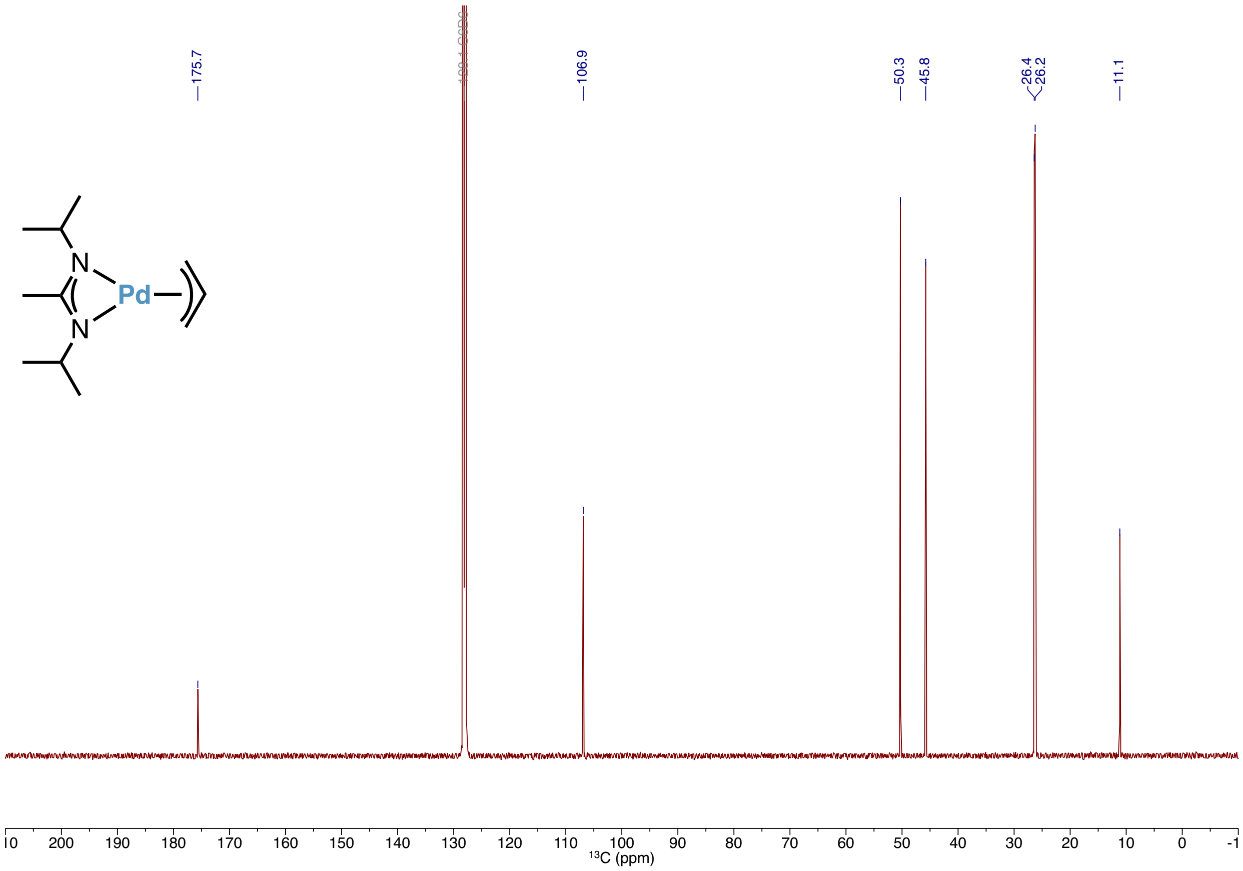

Figure S5. ^{13}C NMR spectrum of **1-Pd** in C_6D_6 at room temperature.

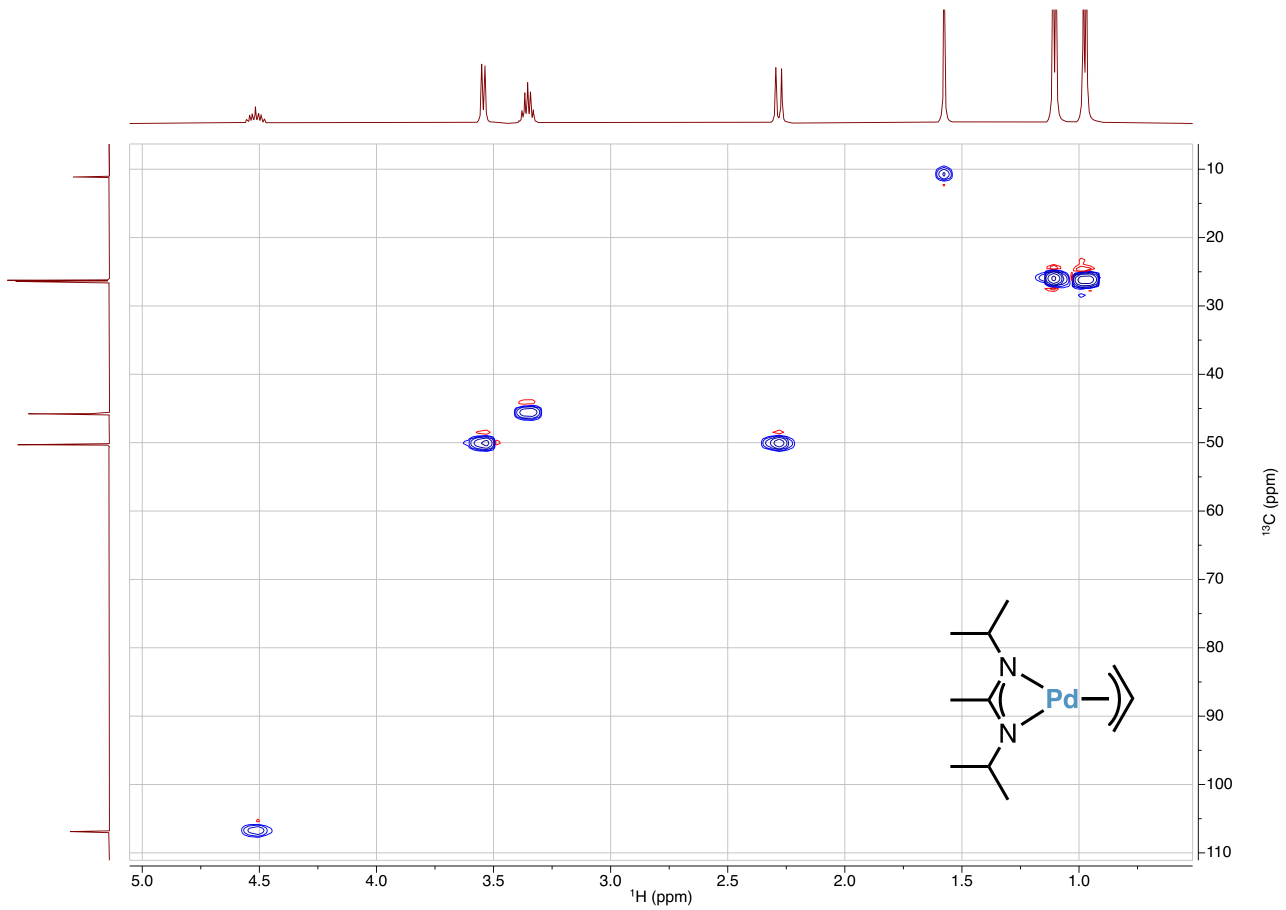

Figure S6. ^{1}H-^{13}C HSQC 2D NMR spectrum of **1-Pd** in C_6D_6 at room temperature.

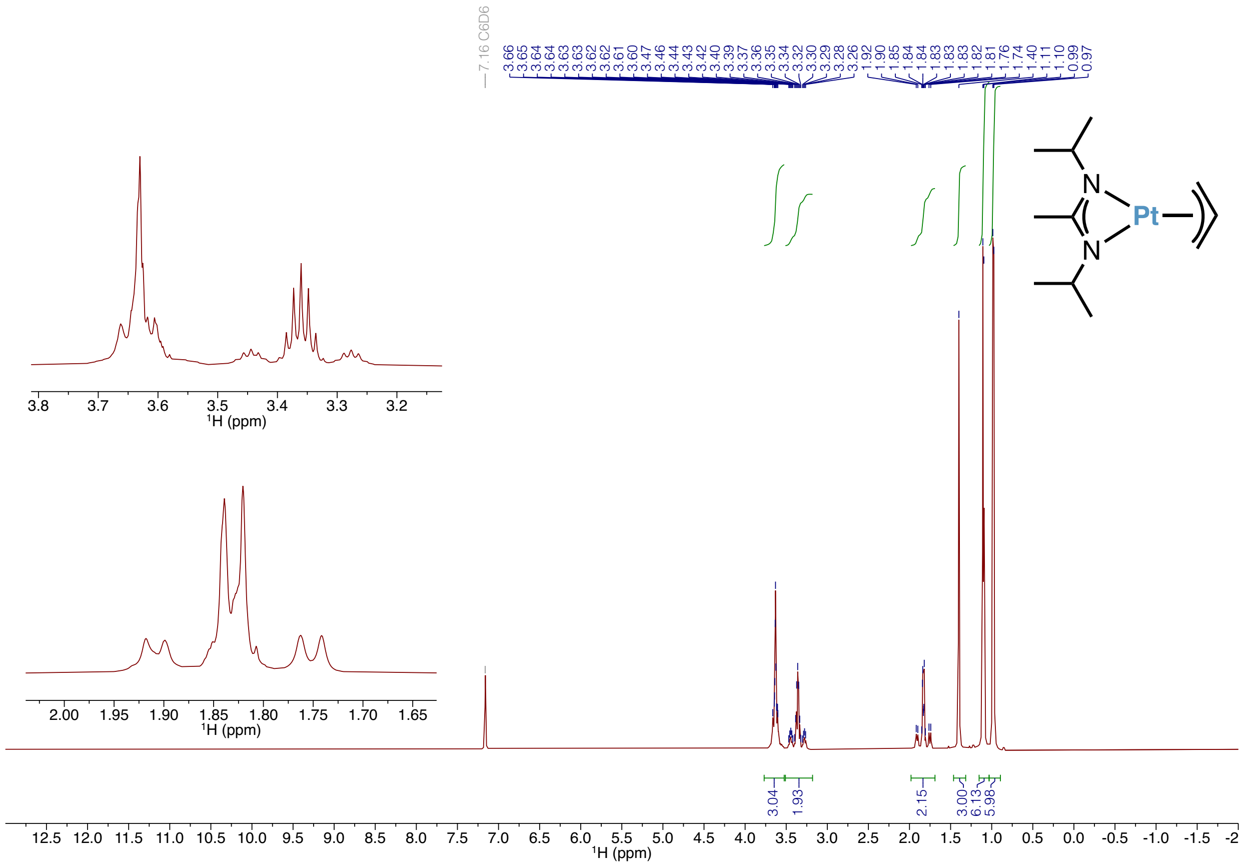

Figure S7. ^{1}H NMR spectrum of **1-Pt** in C_6D_6 at room temperature.

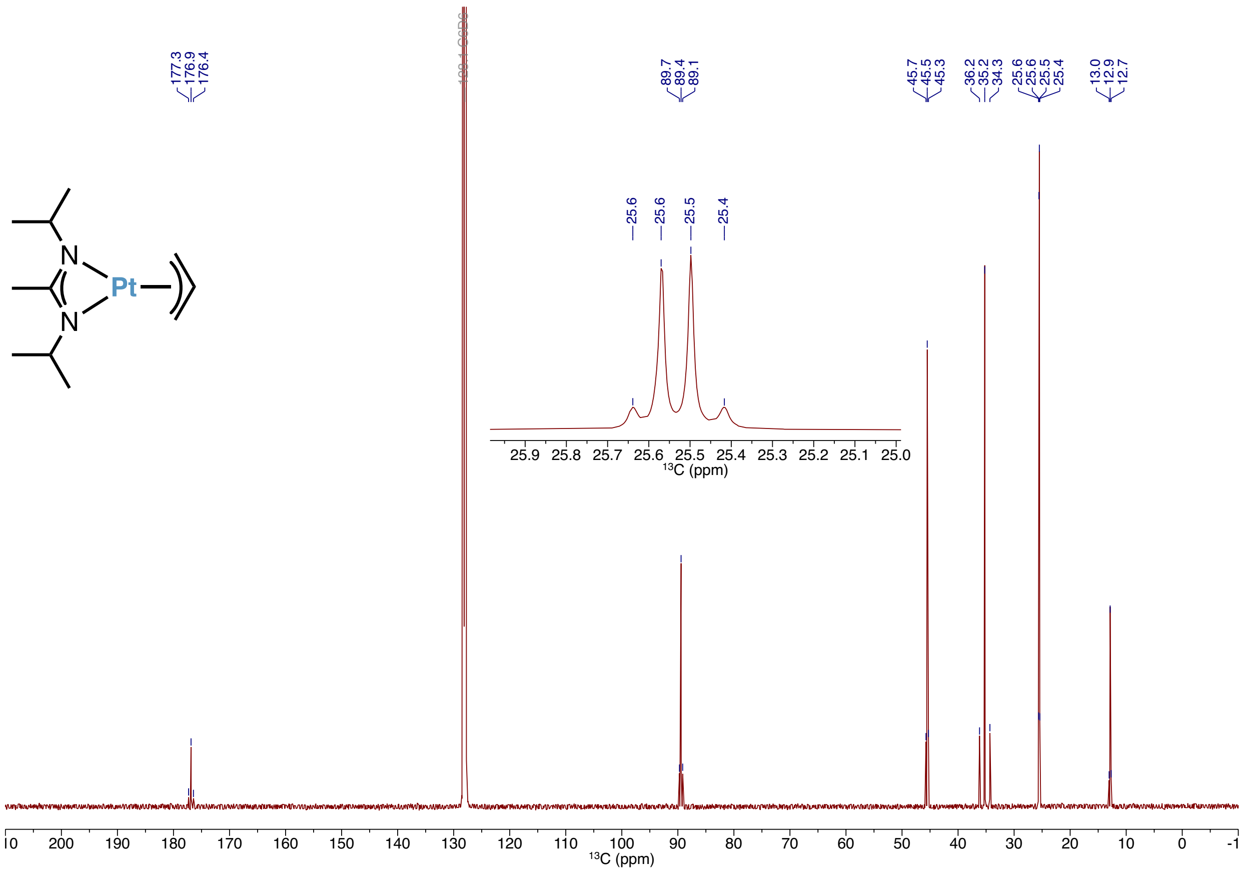

Figure S8. ^{13}C NMR spectrum of **1-Pd** in C_6D_6 at room temperature.

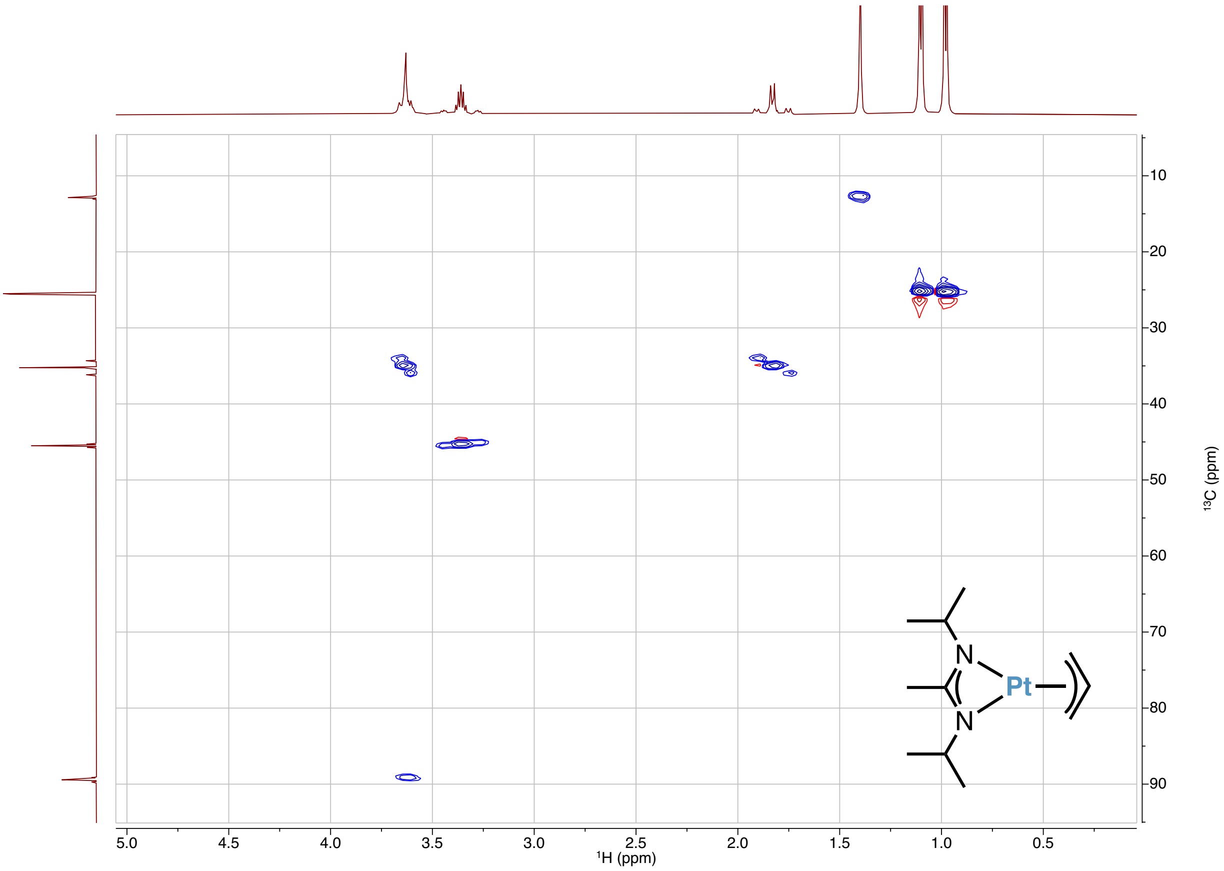

Figure S9. ^{1}H-^{13}C HSQC 2D NMR spectrum of **1-Pt** in C_6D_6 at room temperature.

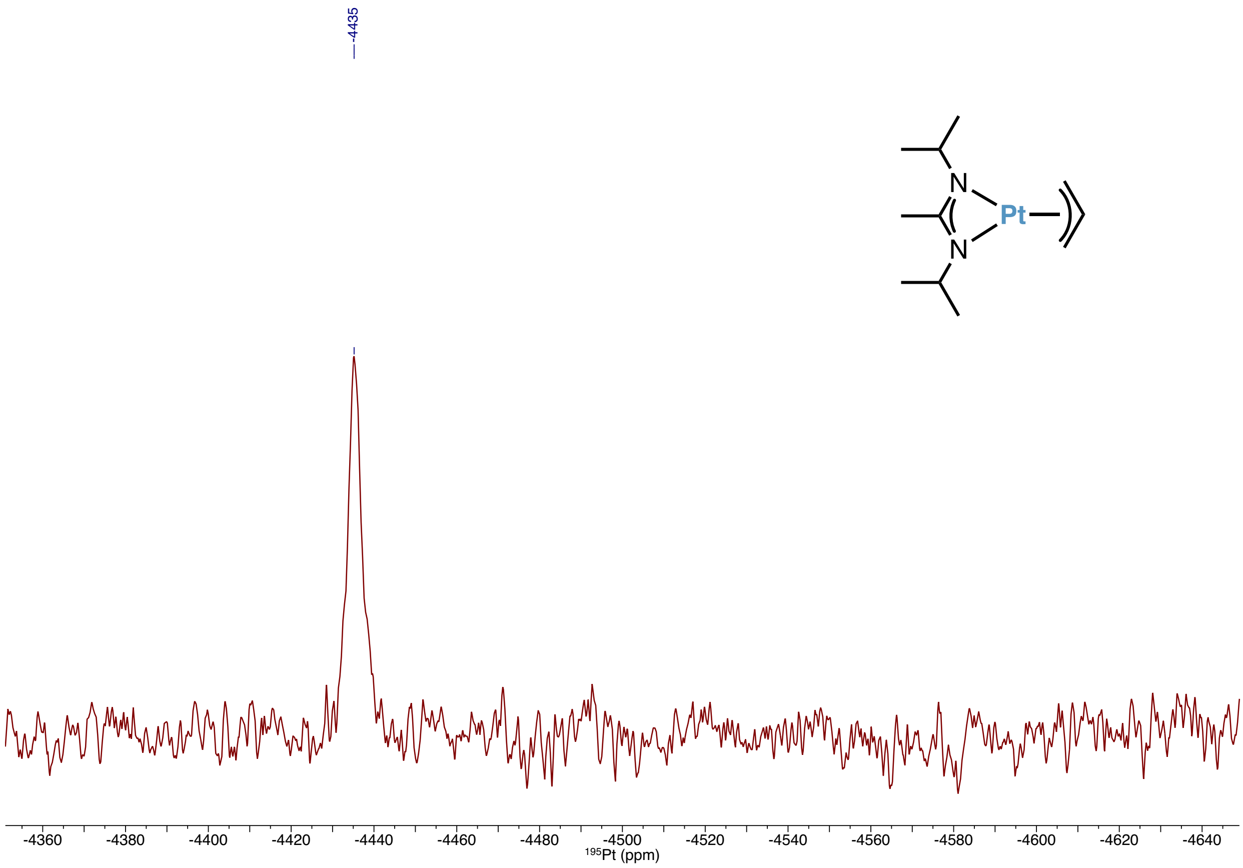

Figure S10. ^{195}Pt NMR spectrum of **1-Pt** in C_6D_6 at room temperature.

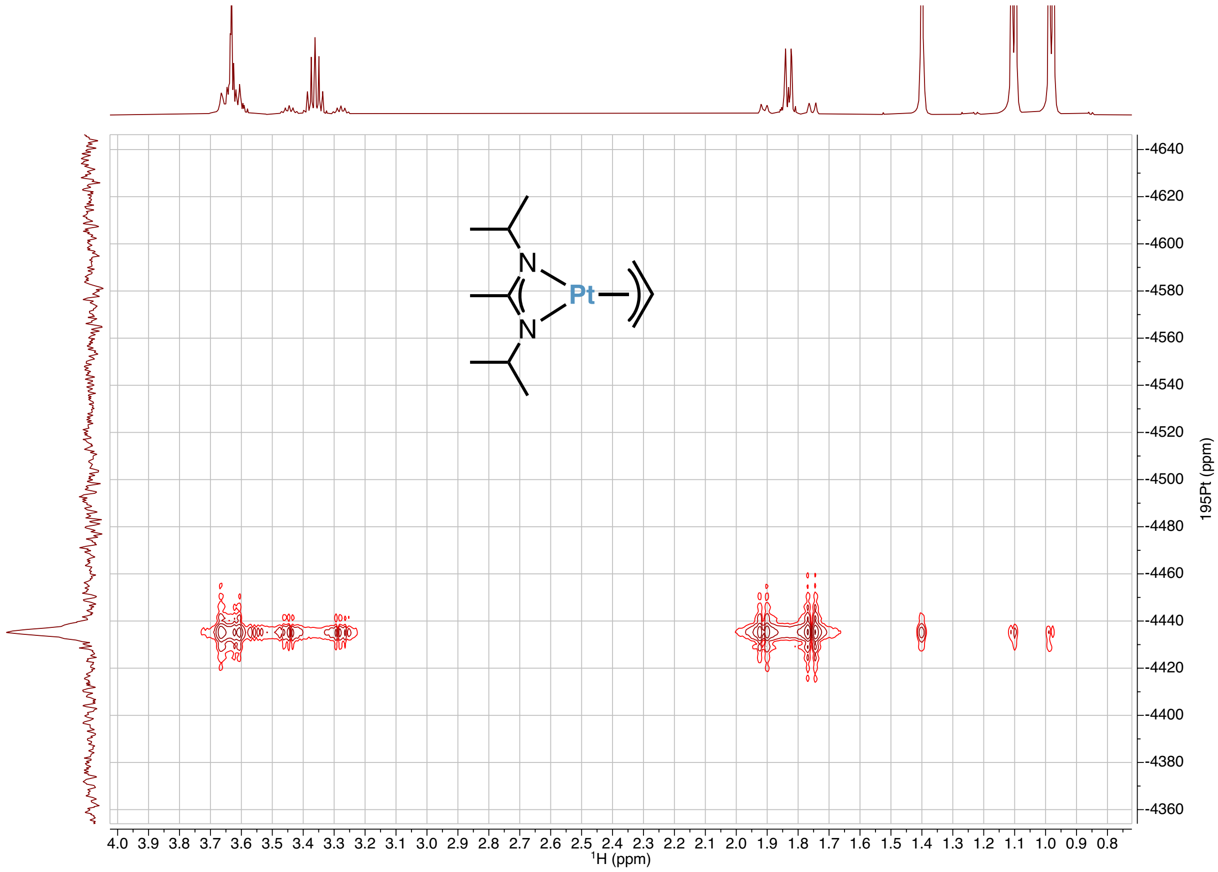

Figure S11. ^{1}H-^{195}Pt HMBC 2D NMR spectrum of **1-Pt** in C_6D_6 at room temperature.

3.2 MAS Solid State NMR Spectra

1-Ni/SiO$_2$

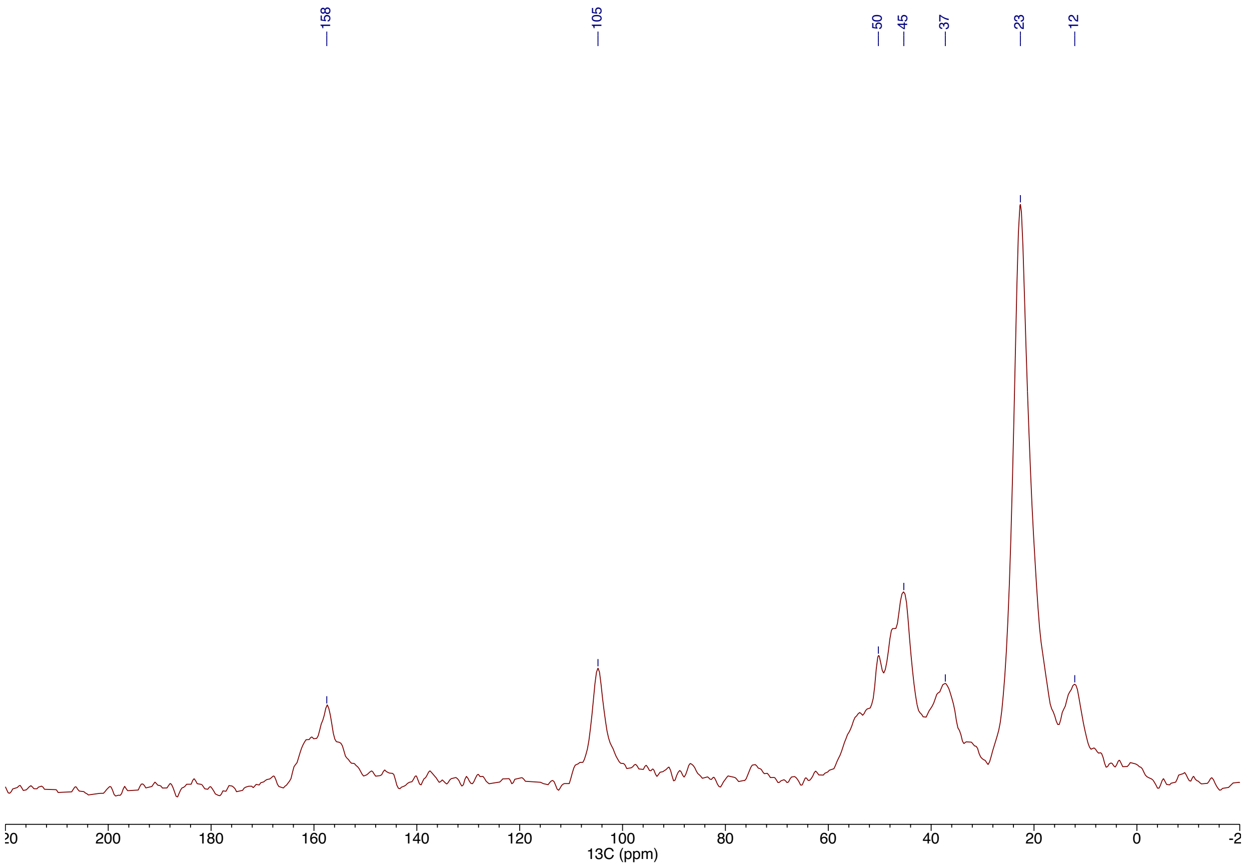

Figure S12. ^{13}C MAS SSNMR spectrum of **1-Ni**/SiO_{2-700}. *NS* = 81920 scans

1-Pd/SiO2

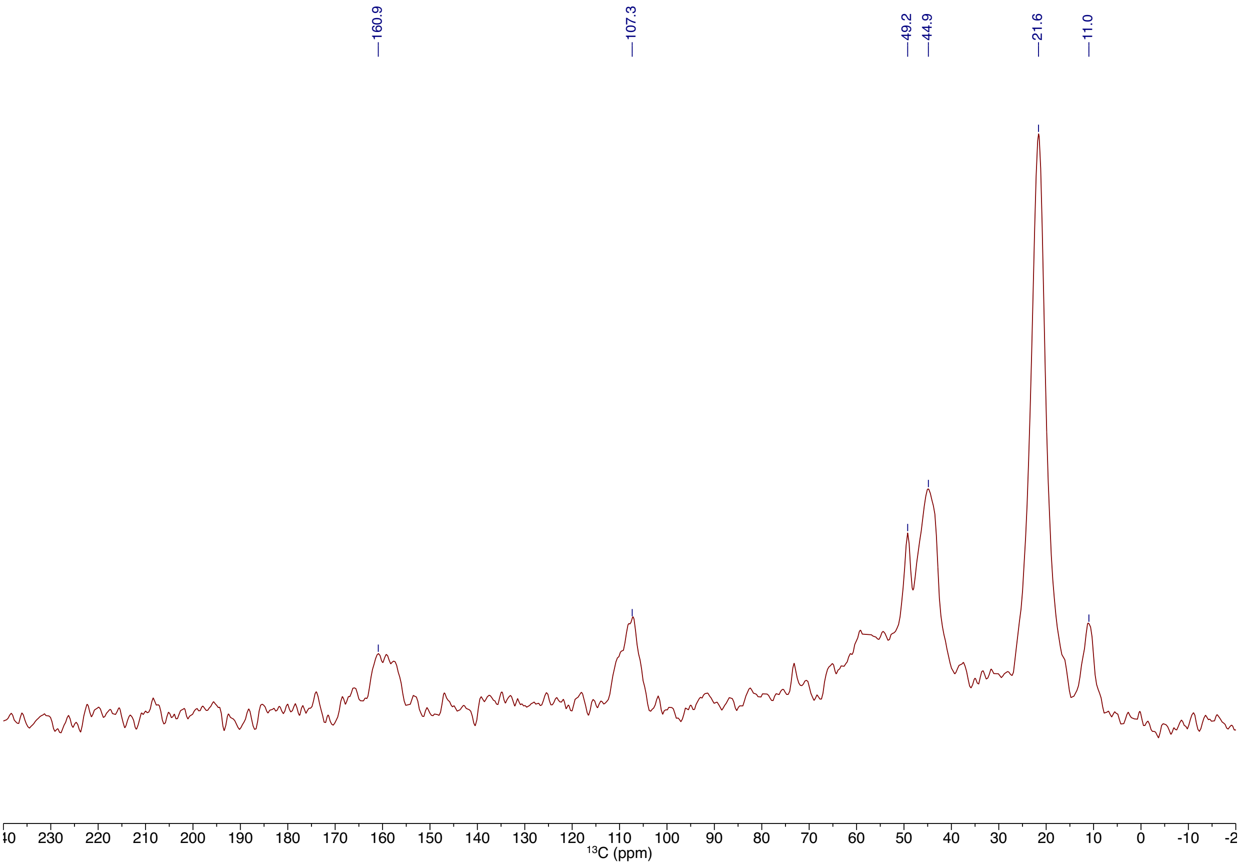

Figure S13. ^{13}C MAS SSNMR spectrum of **1-Pd**/SiO_{2-700}. *NS* = 61440

1-Ni/Al2O3

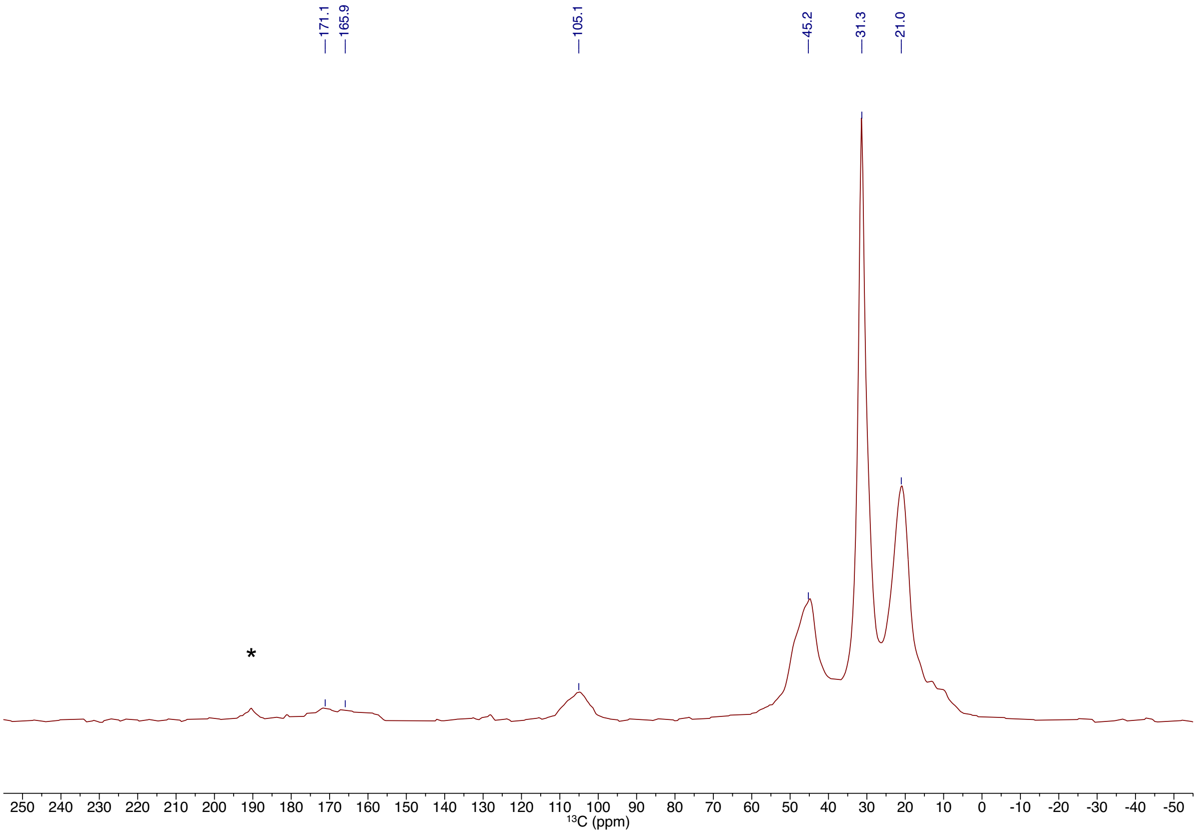

Figure S14. ^{13}C MAS SSNMR spectrum of **1-Ni**/Al_2O_{3-600}. *NS* = 88520. The signal at 191 ppm is a side band.

1-Pd/Al_2O_3

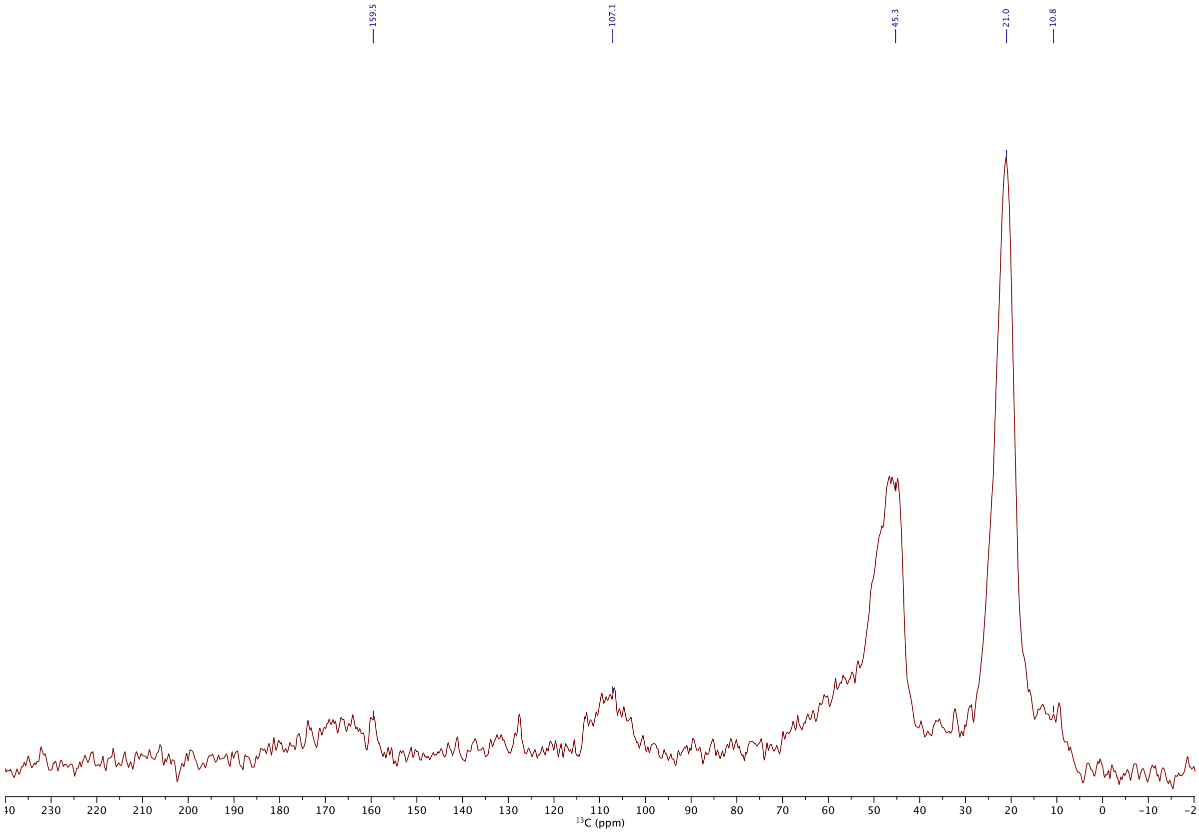

Figure S15. ^{13}C MAS SSNMR spectrum of **1-Pd**/Al_2O_{3-600}. *NS* = 46544.

4 IR Spectra

4.1 IR Spectra of Molecular Precursors and Materials

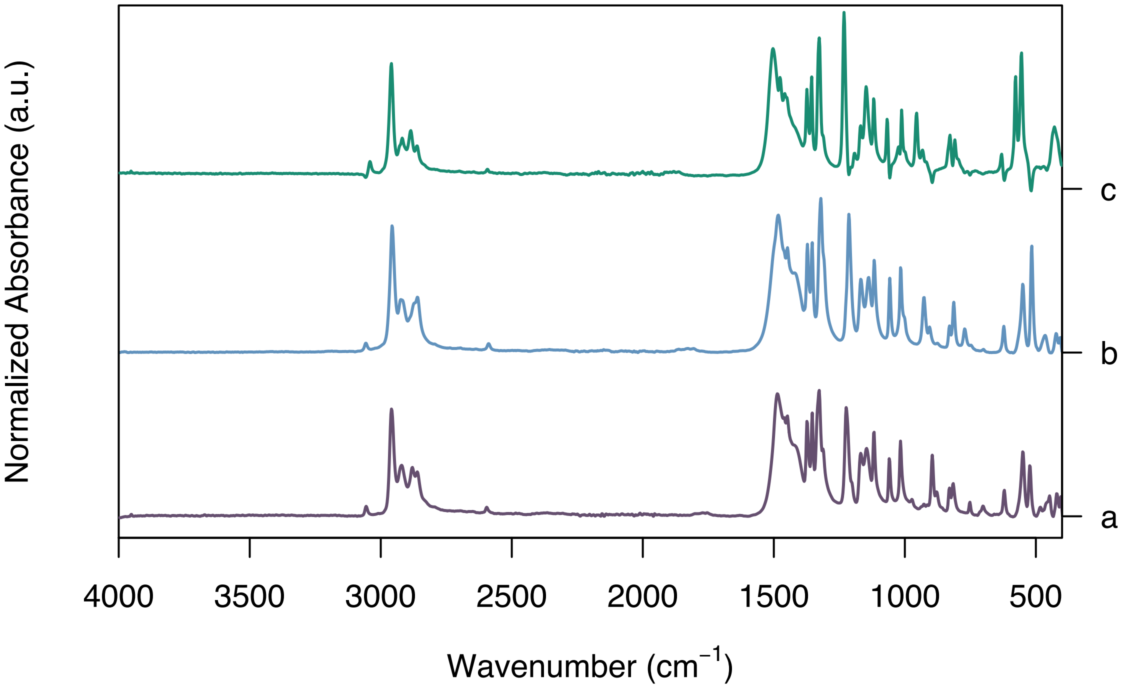

Figure S16. IR Spectra of a) **1-Ni**, b) **1-Pd**, c) **1-Pt**.

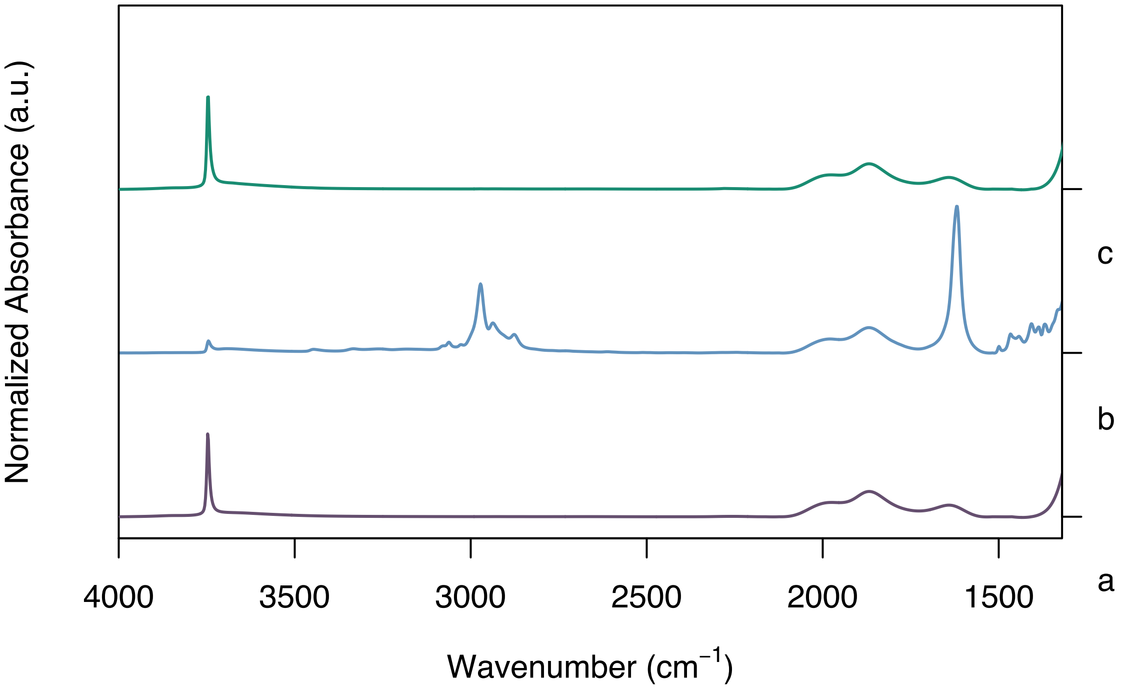

Figure S17. IR Spectra of a) $SiO_{2\text{-}700}$, b) **1-Ni**/$SiO_{2\text{-}700}$, c) Ni/SiO_2.

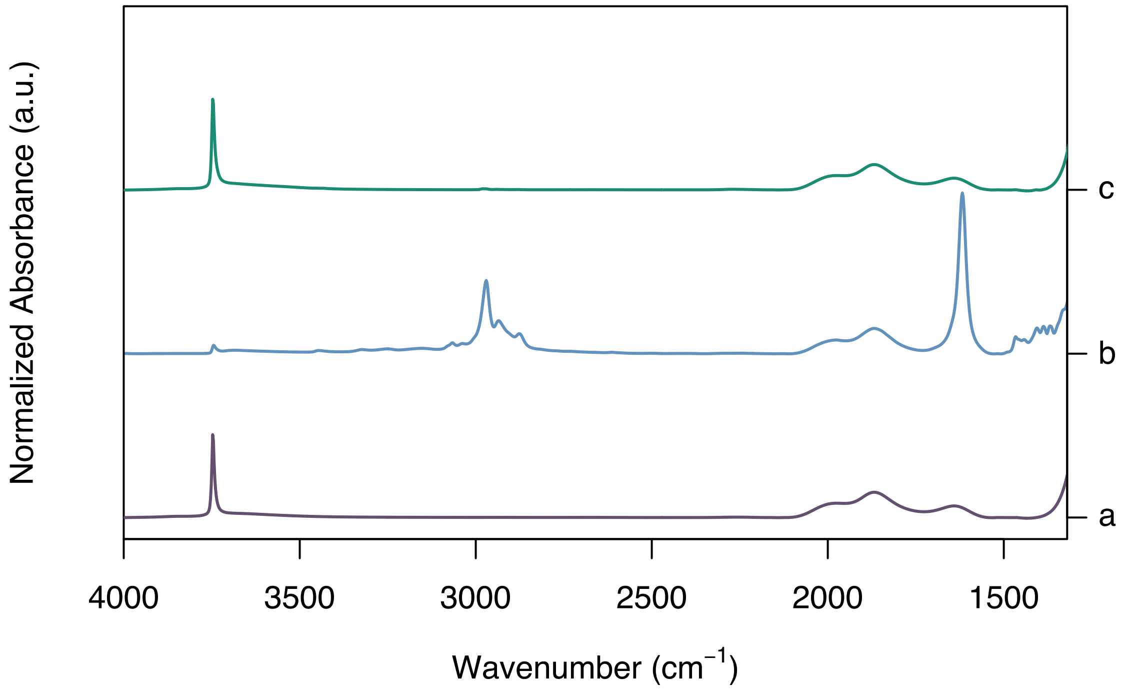

Figure S18. IR Spectra of a) $SiO_{2\text{-}700}$, b) **1-Pd**/$SiO_{2\text{-}700}$, c) Pd/SiO_2.

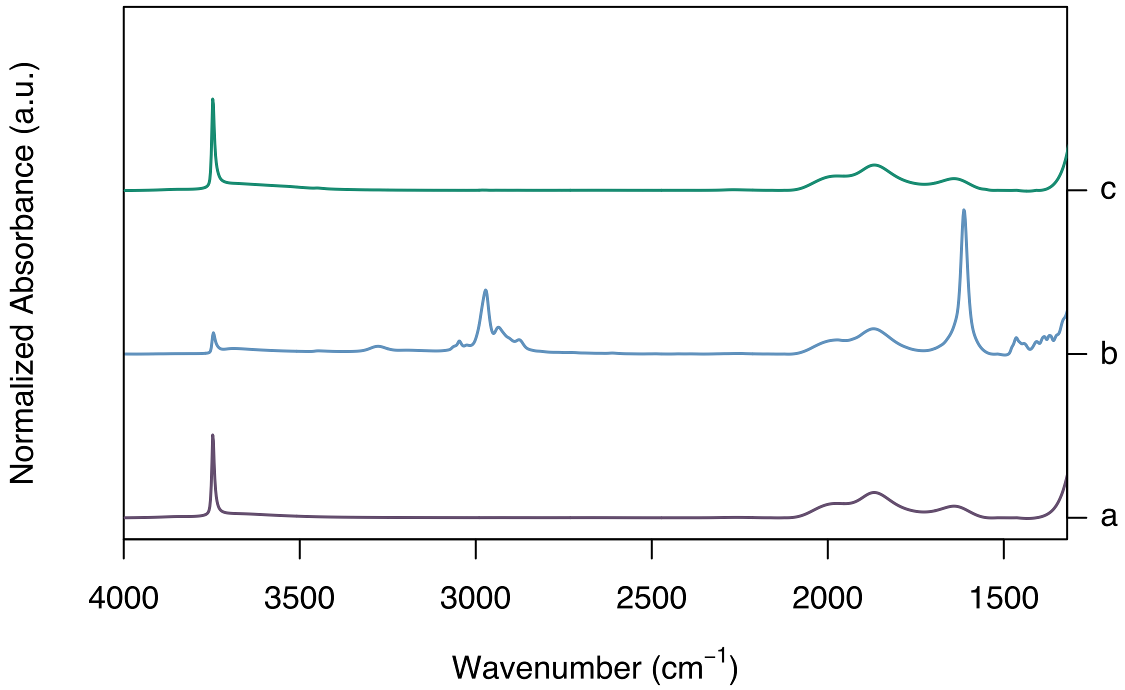

Figure S19. IR Spectra of a) $SiO_{2\text{-}700}$, b) **1-Pt**/$SiO_{2\text{-}700}$, c) Pt/SiO_2.

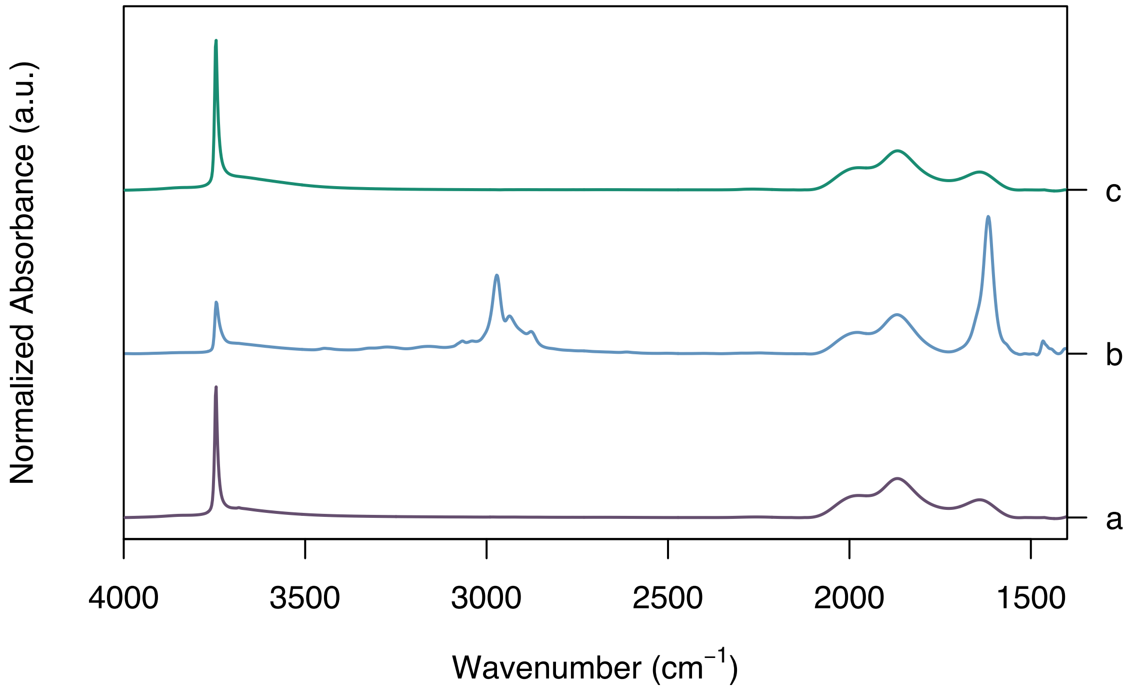

Figure S20. IR Spectra of a) Ga@SiO_2, b) **1-Pd**/Ga@SiO_2, c) PdGa/SiO_2.

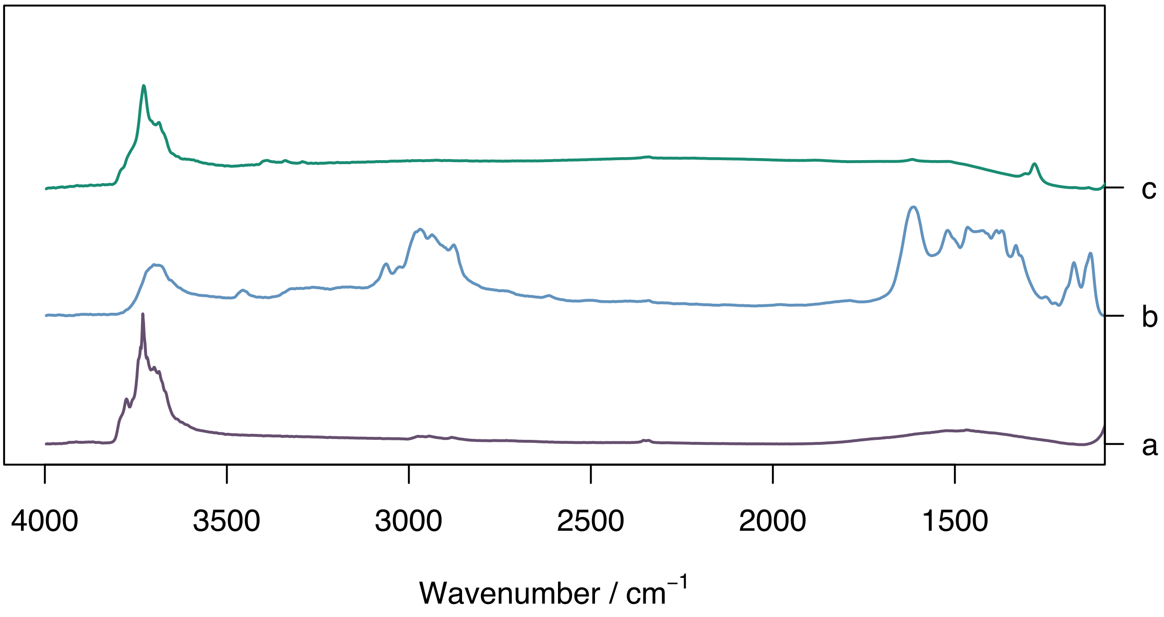

Figure S21. IR Spectra of a) Al_2O_{3-600}, b) **1-Ni**/Al_2O_{3-600}, c) Ni/Al_2O_{3-600}.

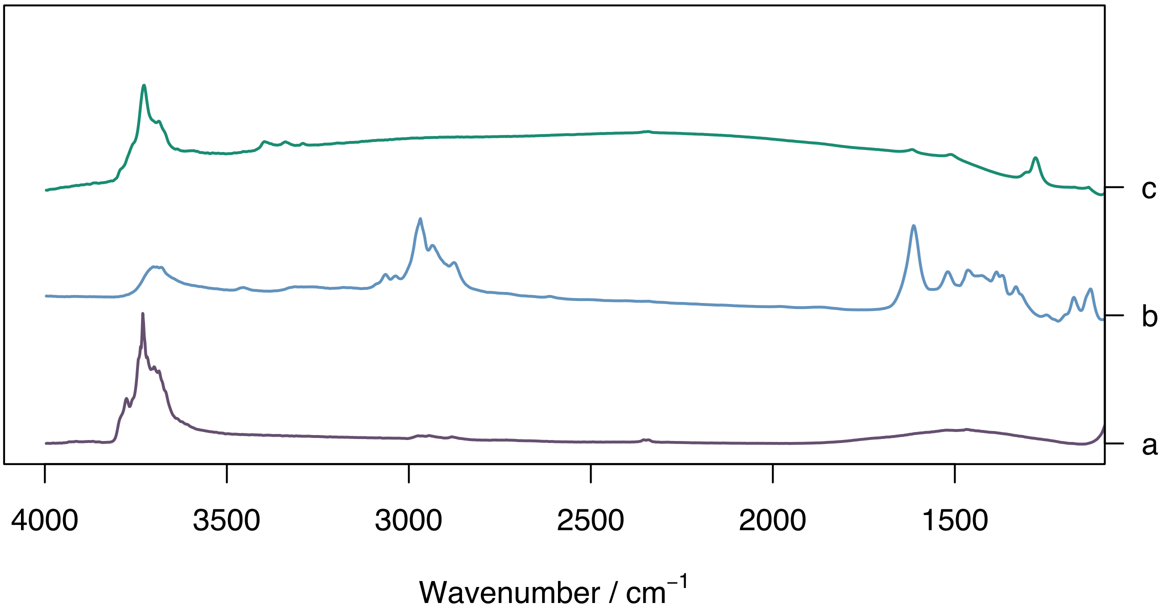

Figure S22. IR Spectra of a) Al_2O_{3-600}, b) **1-Pd**/Al_2O_{3-600}, c) **Pd**/Al_2O_{3-600}.

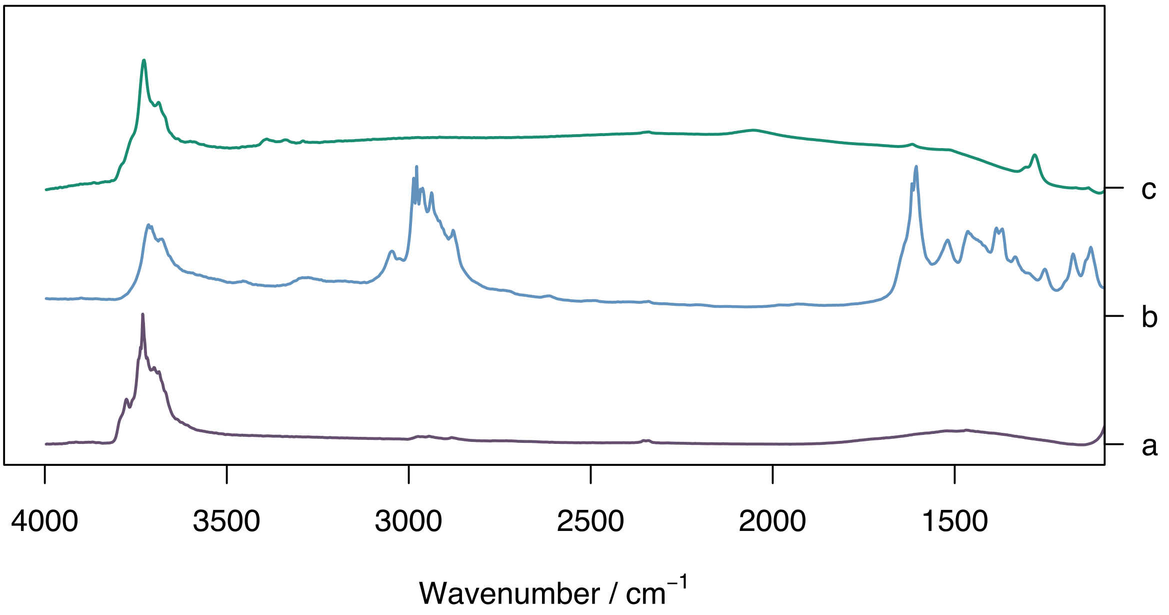

Figure S23. IR Spectra of a) Al_2O_{3-600}, b) **1-Pt**/Al_2O_{3-600}, c) **Pt**/Al_2O_{3-600}.

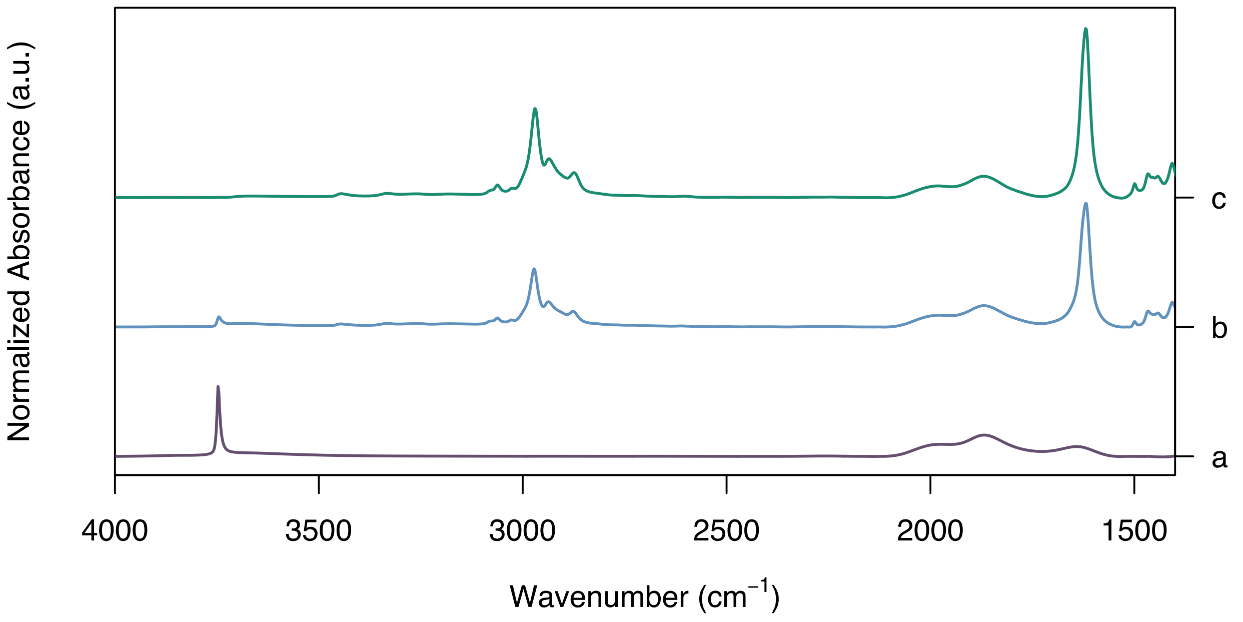

Figure S24. IR Spectra of a) SiO_{2-700}, b) **1-Ni**/SiO_{2-700} obtained from solution grafting c) **1-Ni**/SiO_{2-700} obtained from grafting via sublimation.

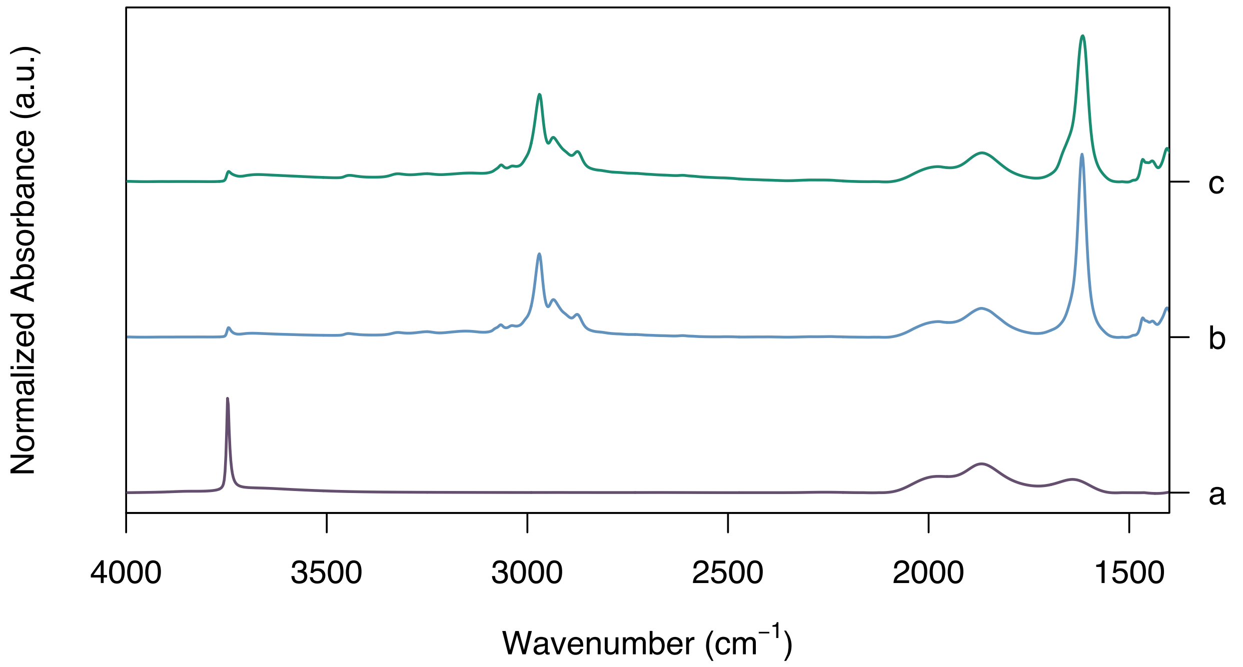

Figure S25. IR Spectra of a) SiO_{2-700}, b) **1-Pd**/SiO_{2-700} obtained from solution grafting c) **1-Pd**/SiO_{2-700} obtained from grafting via sublimation.

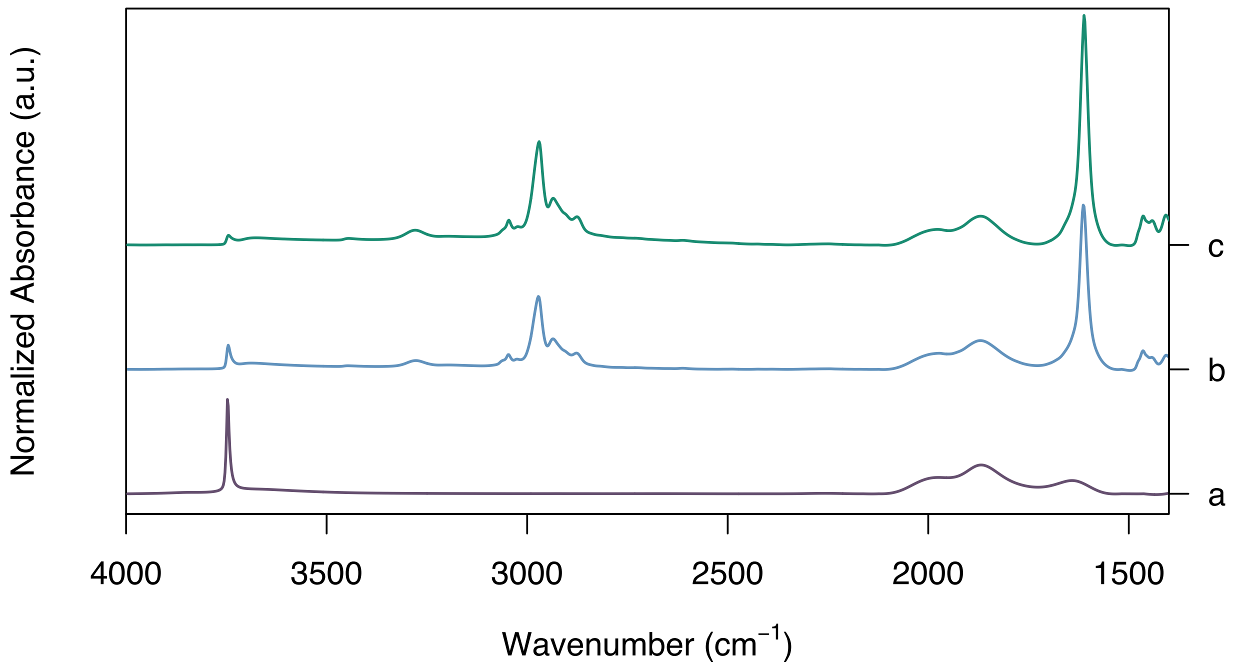

Figure S26. IR Spectra of a) SiO_{2-700}, b) **1-Pt**/SiO_{2-700} obtained from solution grafting c) **1-Pt**/SiO_{2-700} obtained from grafting via sublimation.

4.2 CO-Adsorption IR Spectra

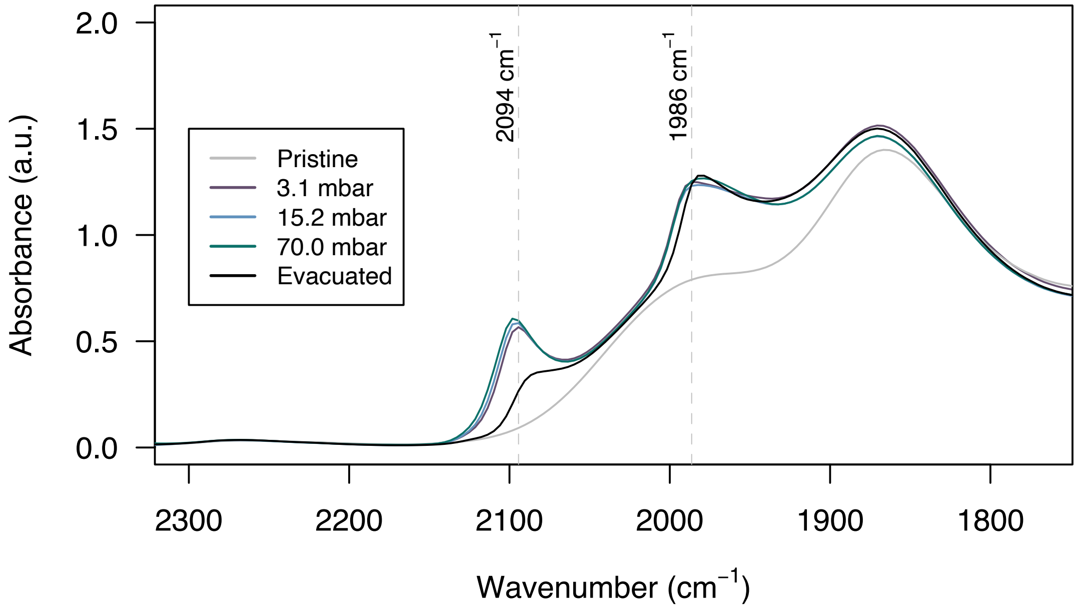

Figure S27. CO-IR spectra of CO adsorbed on **Pd**/SiO_2 at various pressures.

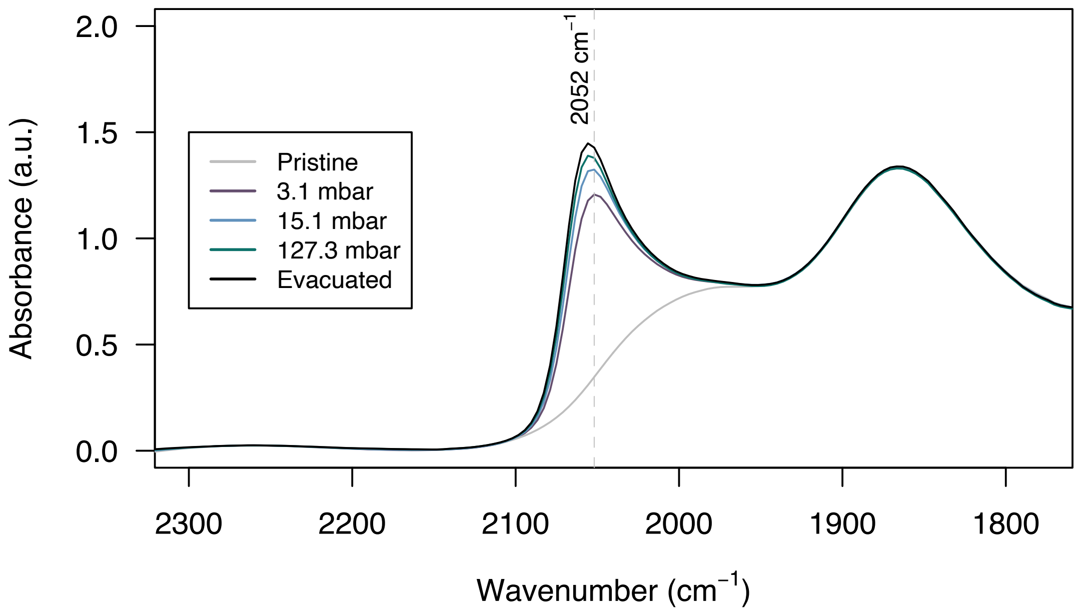

Figure S28. CO-IR spectra of CO adsorbed on **PdGa**/SiO_2 at various pressures.

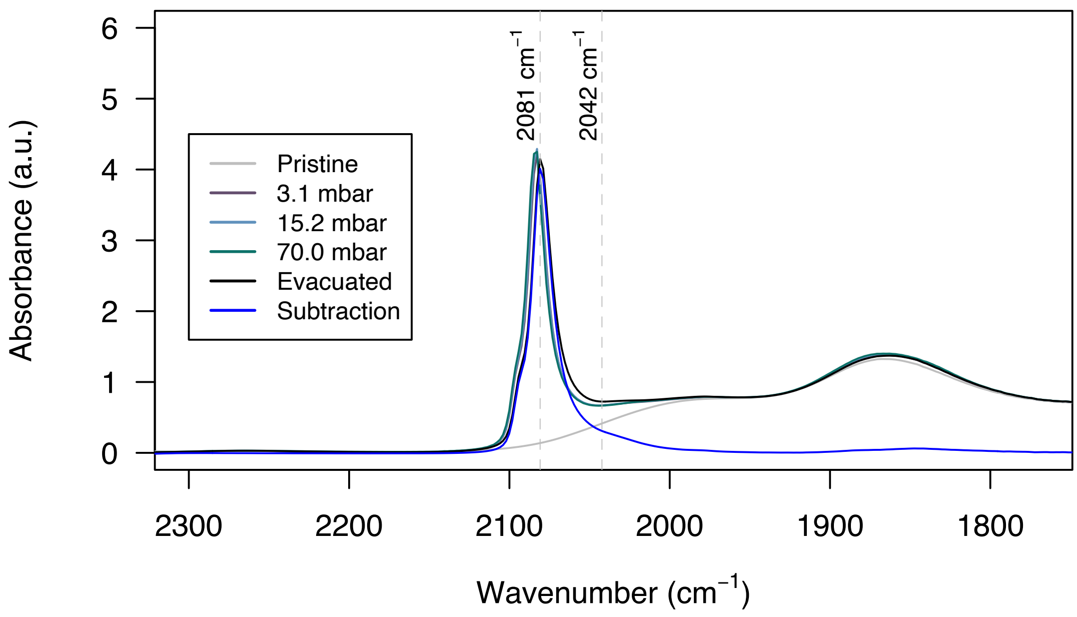

Figure S29. CO-IR spectra of CO adsorbed on **Pt**/SiO_2 at various pressures. The blue spectrum was obtained by subtraction of the pristine spectrum (gray) from the spectrum after evacuation (black).

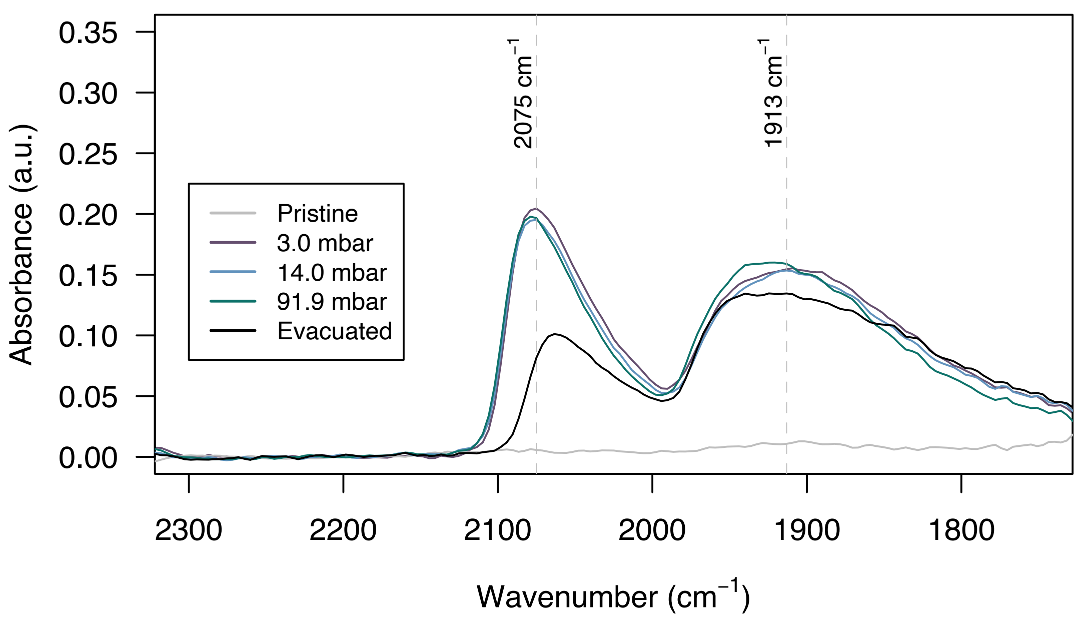

Figure S30. CO-IR spectra of CO adsorbed on **Pd**/Al_2O_3 at various pressures.

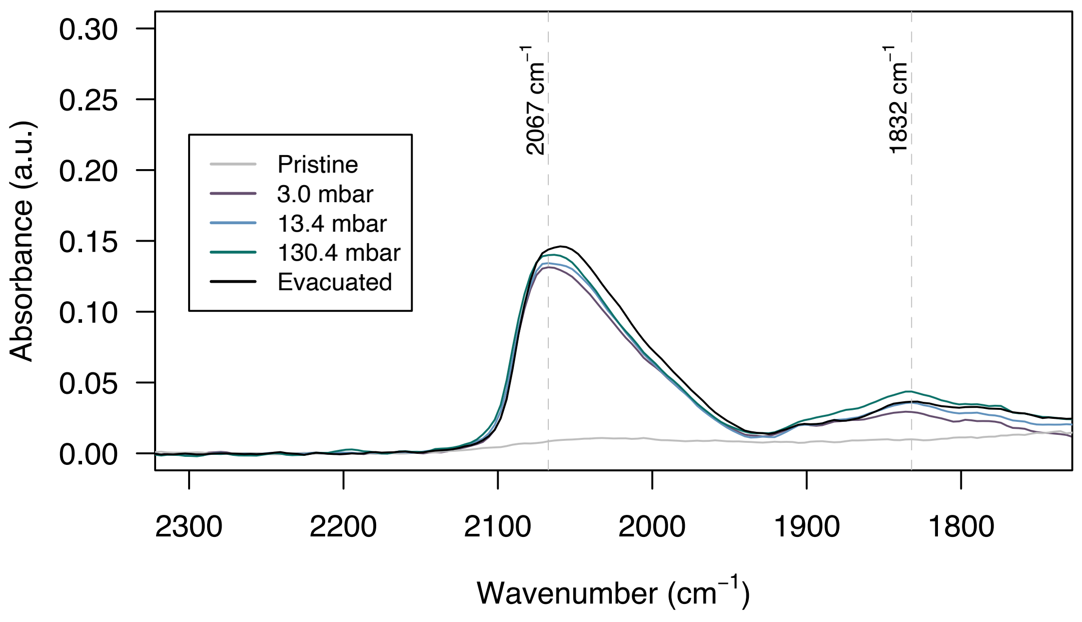

Figure S31. CO-IR spectra of CO adsorbed on **Pt**/Al_2O_3 at various pressures.

5 Electron Microscopy

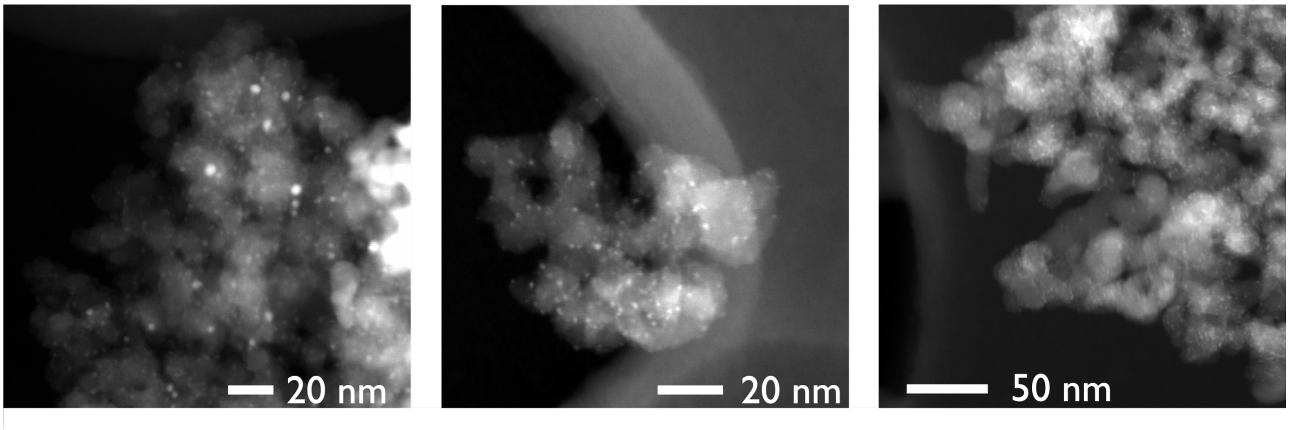

Figure S32. HAADF-STEM Images of Ni/SiO_2.

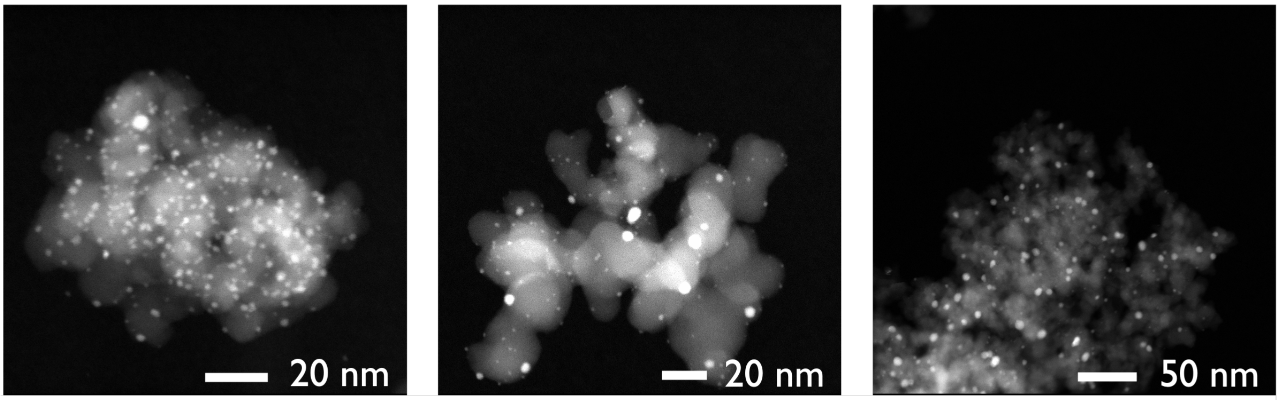

Figure S33. HAADF-STEM Images of Pd/SiO_2.

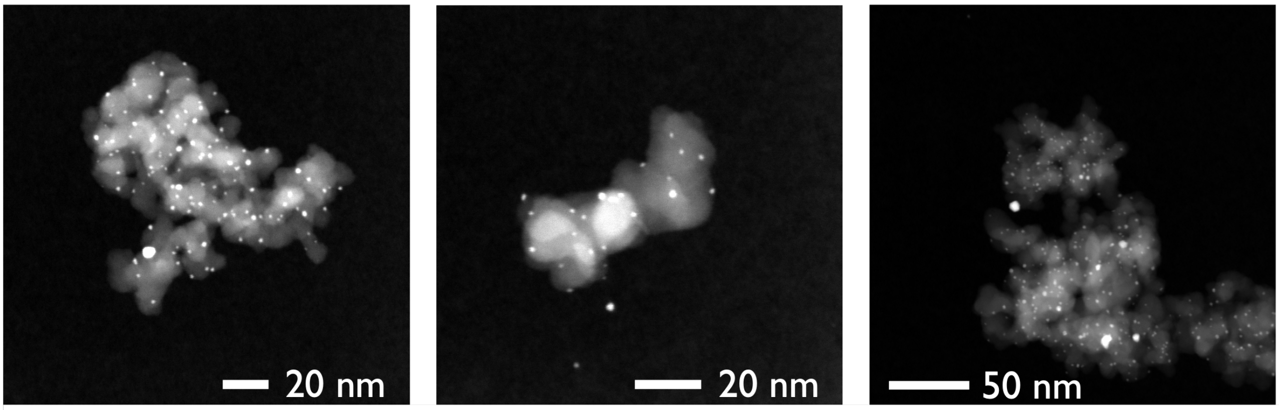

Figure S34. HAADF-STEM Images of Pt/SiO_2.

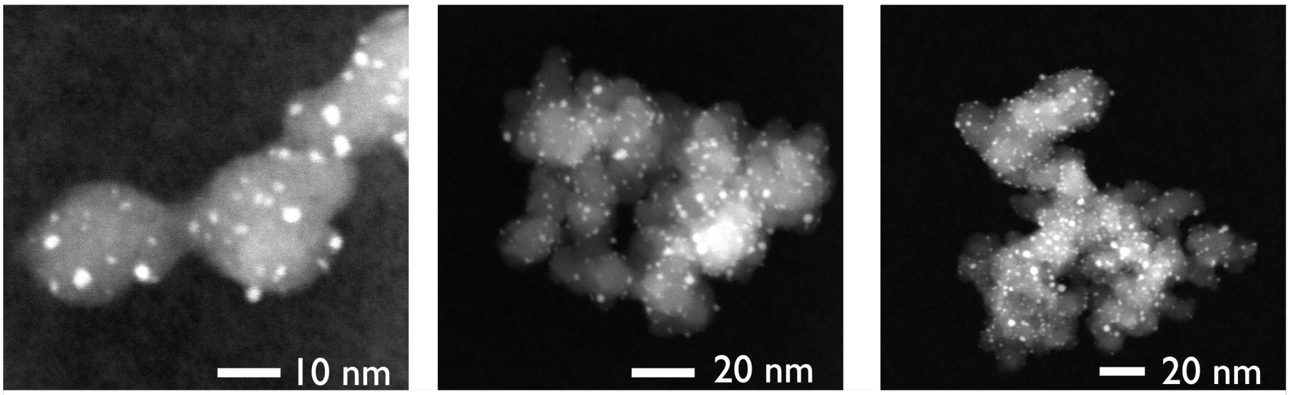

Figure S35. HAADF-STEM Images of $PdGa/SiO_2$.

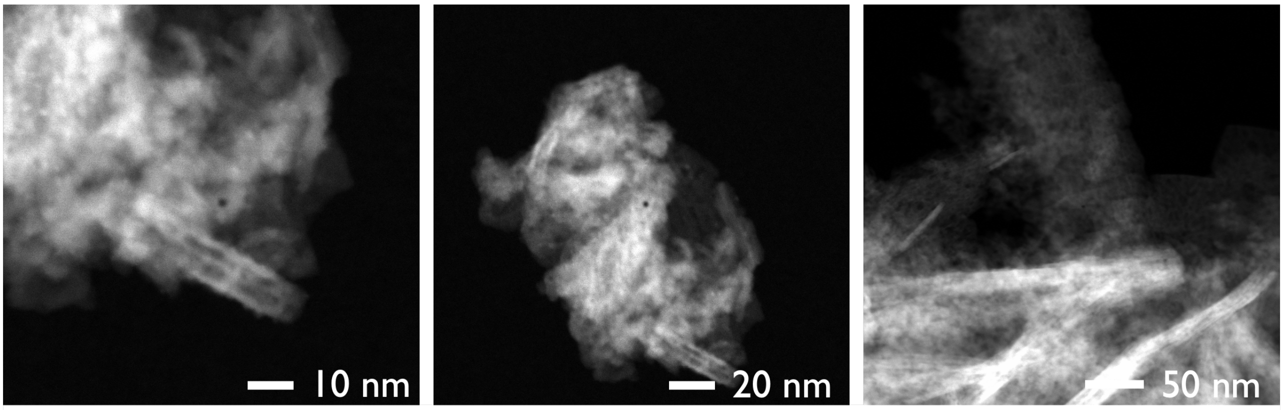

Figure S36. HAADF-STEM Images of Ni/Al_2O_3.

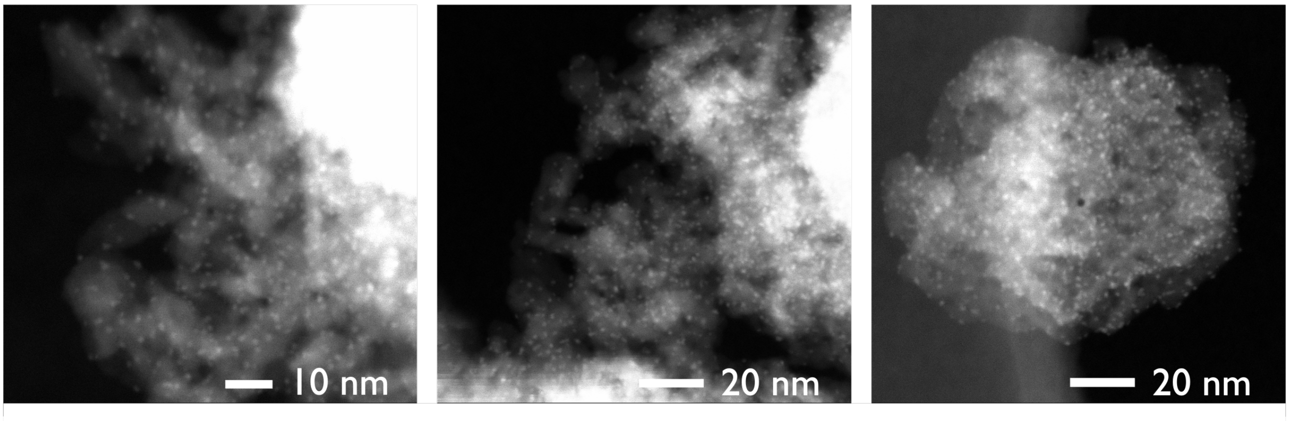

Figure S37. HAADF-STEM Images of Pd/Al_2O_3.

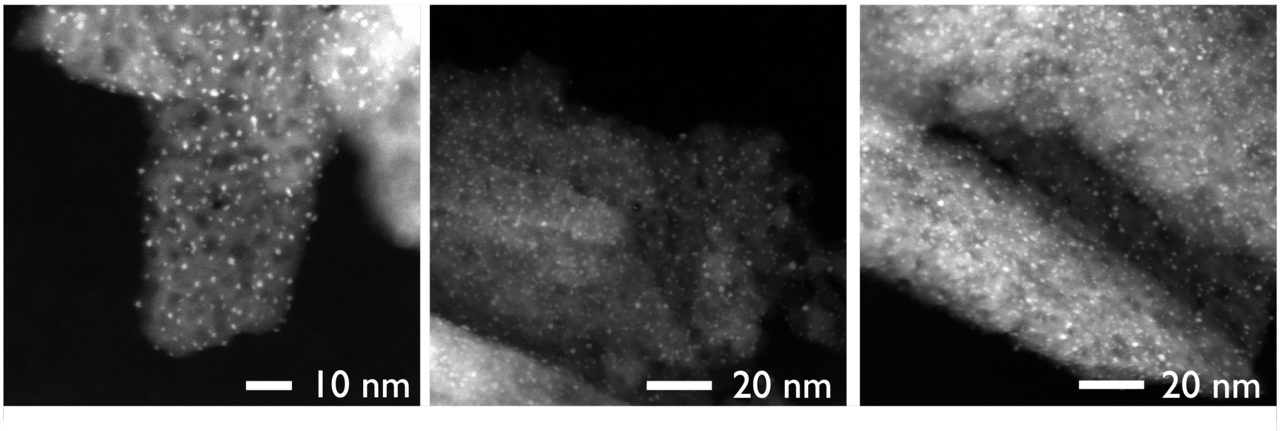

Figure S38. HAADF-STEM Images of Pt/Al_2O_3.

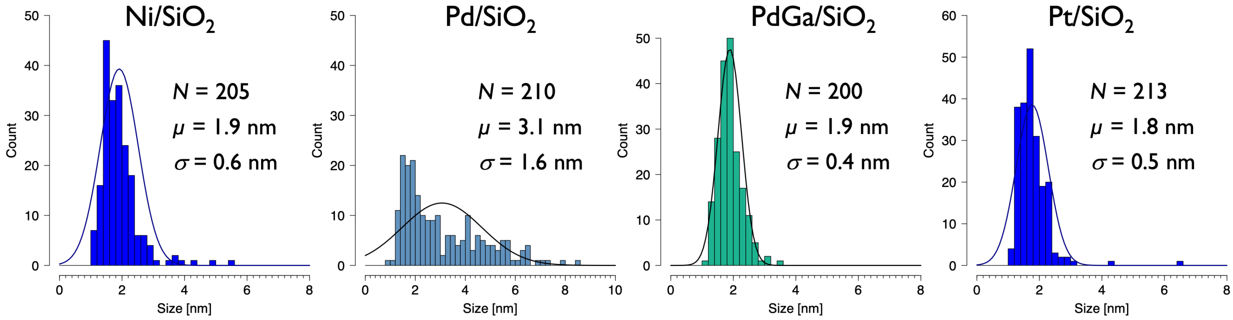

Figure S39. Particle size distributions of SiO_2-supported materials from measuring the size of particles. The drawn lines indicate the normal distribution (parameters shown on plot) fitted on the data.

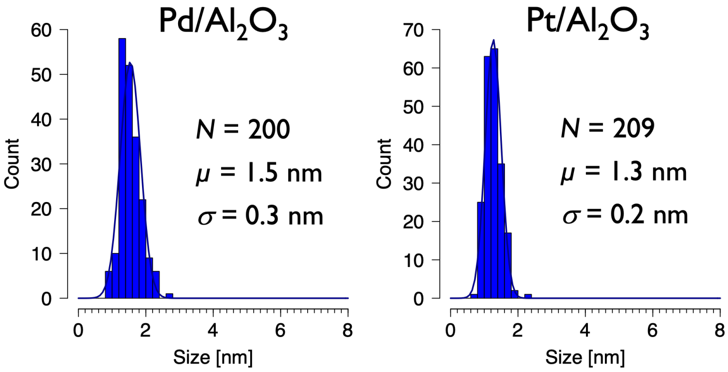

Figure S40. Particle size distribution of Al_2O_3-supported materials from measuring the size of particles. The drawn lines indicate the normal distribution (parameters shown on plot) fitted on the data. Note that for Ni/Al_2O_3, the combination of very small particles and low z-contrast did not allow for the determination of particle size in the same way.

6 H_2 Chemisorption

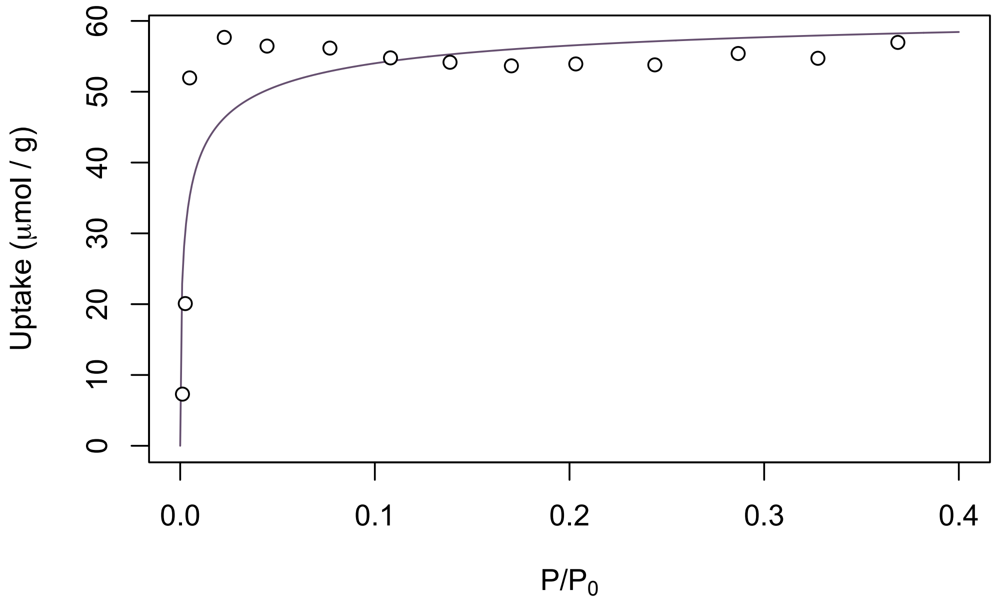

Figure S41. Hydrogen chemisorption of Pd/SiO_2. Fit parameters of dissociative Langmuir model (solid line): K_{H2} = 315 (standard deviation = 253), $Q_{H2,\ max}$ = 63.6 µmol/g (std. dev. = 5.0 µmol/g)

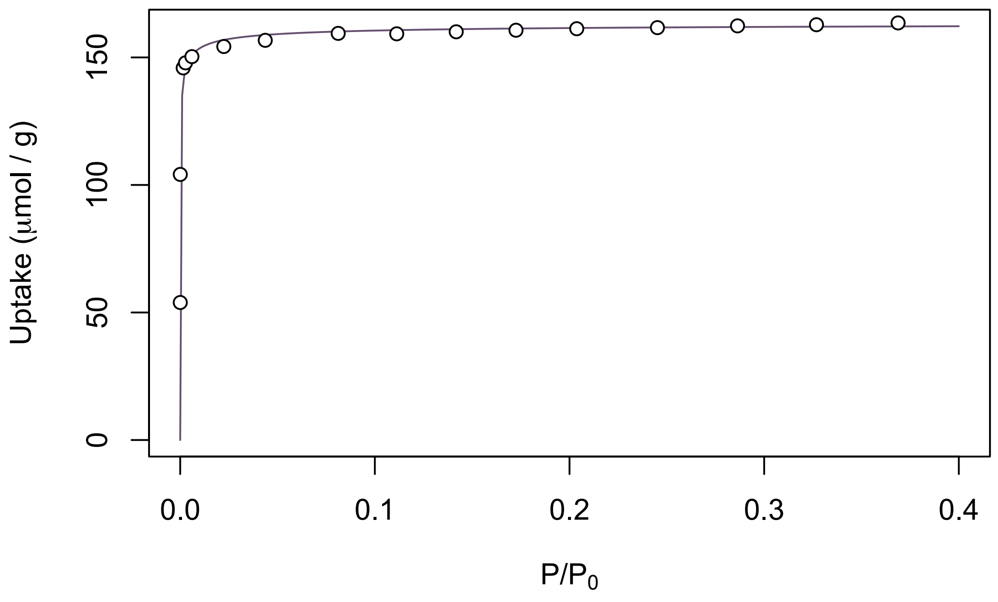

Figure S42. Hydrogen chemisorption (adsorption data as white circles) of Pd/Al_2O_3. Fit parameters of dissociative Langmuir model (solid line): K_{H2} = 21800 (standard deviation = 6900), $Q_{H2,\ max}$ = 163.9 µmol/g (std. dev. = 2.8 µmol/g)

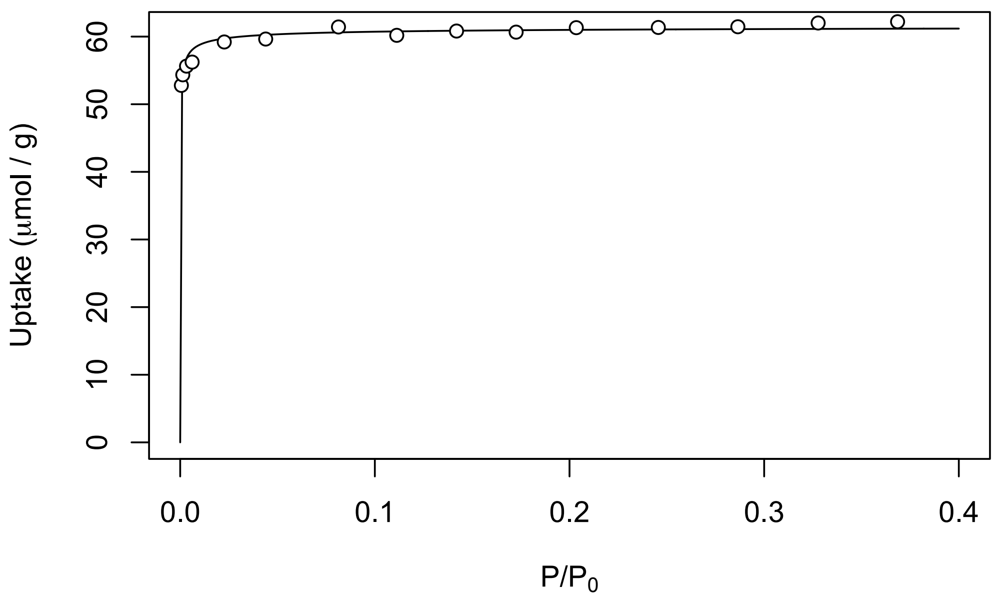

Figure S43. Hydrogen chemisorption (adsorption data as white circles) of Pt/SiO_2. Fit parameters of dissociative Langmuir model (solid line): K_{H2} = 42000 (standard deviation = 7700), $Q_{H2, max}$ = 61.7 μmol/g (std. dev. = 0.3 μmol/g)

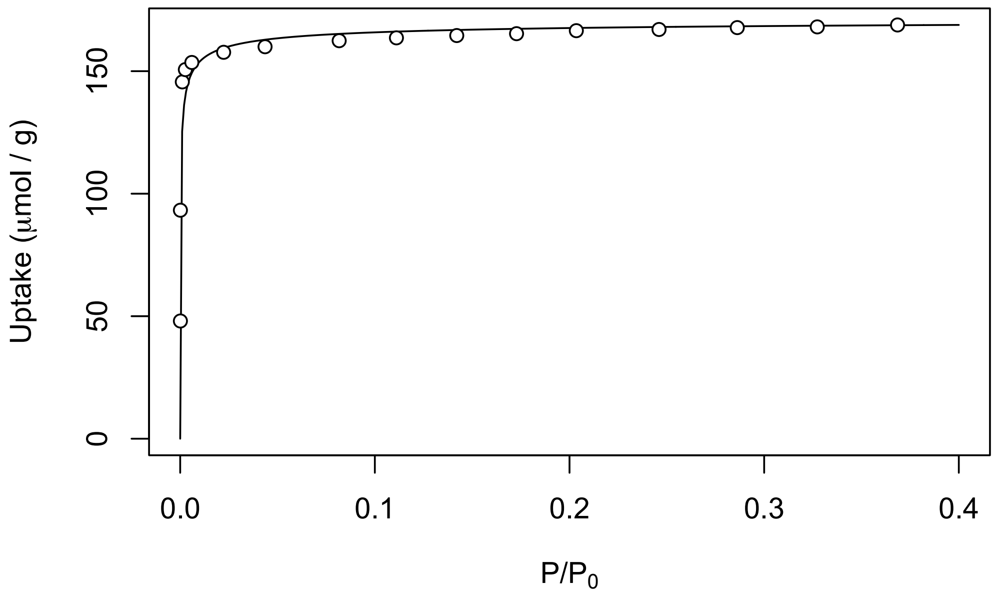

Figure S44. Hydrogen chemisorption (adsorption data as white circles) of Pt/Al_2O_3. Fit parameters of dissociative Langmuir model (solid line): K_{H2} = 7200 (standard deviation = 2500), $Q_{H2, max}$ = 172.0 μmol/g (std. dev. = 3.8 μmol/g)

Table S1. Chemisorption data of Pd and Pt materials.

Material	Ratio (H/M)	Dispersion (%)	Particle Size (nm)
Pd/SiO_2	0.53	51	2.1
Pd/Al_2O_3	1.51	N.A.	N.A.
Pt/SiO_2	0.60	56	1.9
Pt/Al_2O_3	1.57	88	0.9

7 Thermogravimetric Analysis (TGA)

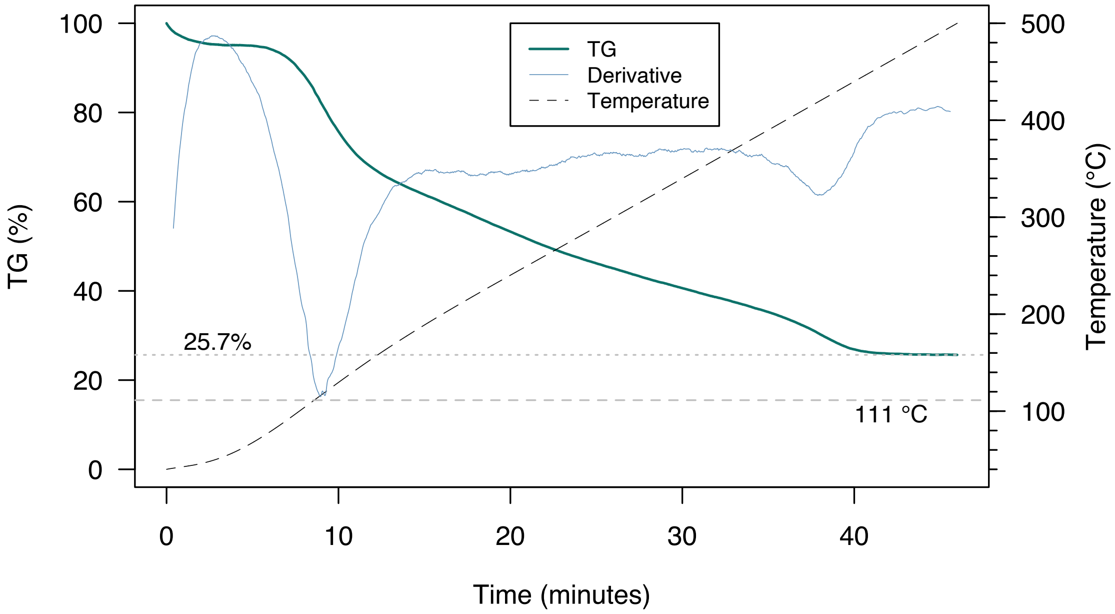

Figure S45. TGA curve of **1-Ni** and derivative indicating the maximum rate of weight loss occurs around 111 °C.

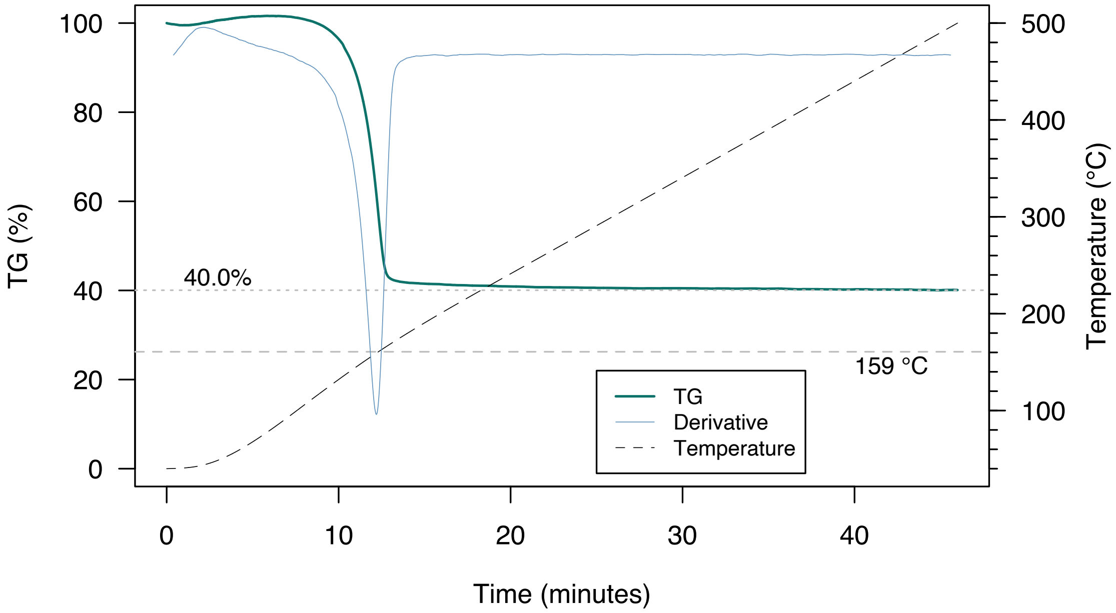

Figure S46. TGA curve of **1-Pd** and derivative indicating the maximum rate of weight loss occurs around 159 °C.

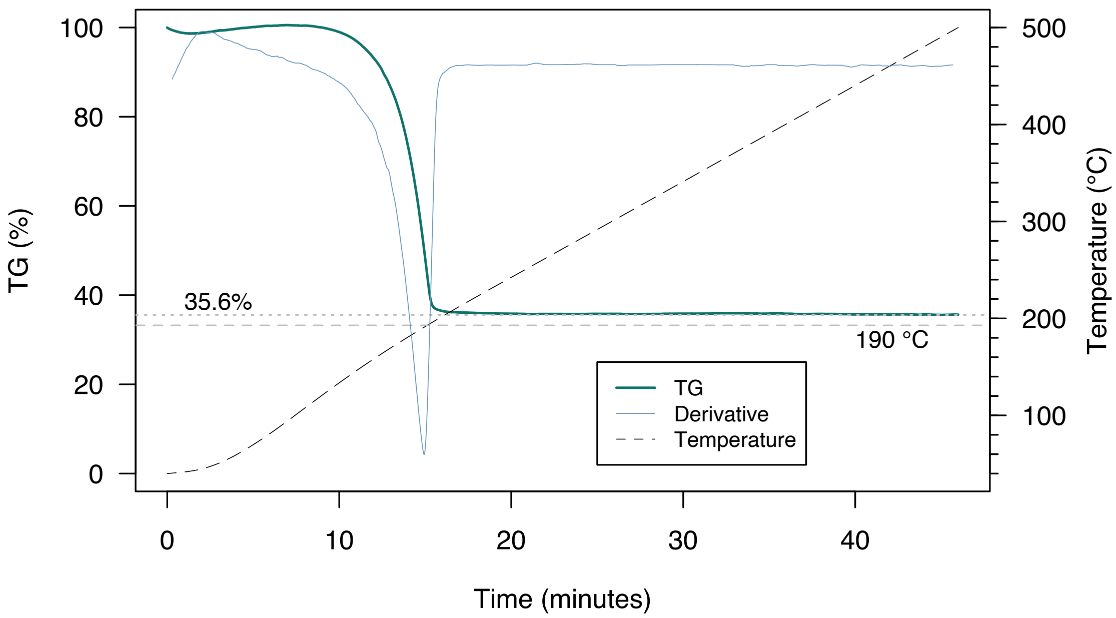

Figure S47. TGA curve of **1-Pt** and derivative indicating the maximum rate of weight loss occurs around 190 °C.

8 UV-Vis Spectra

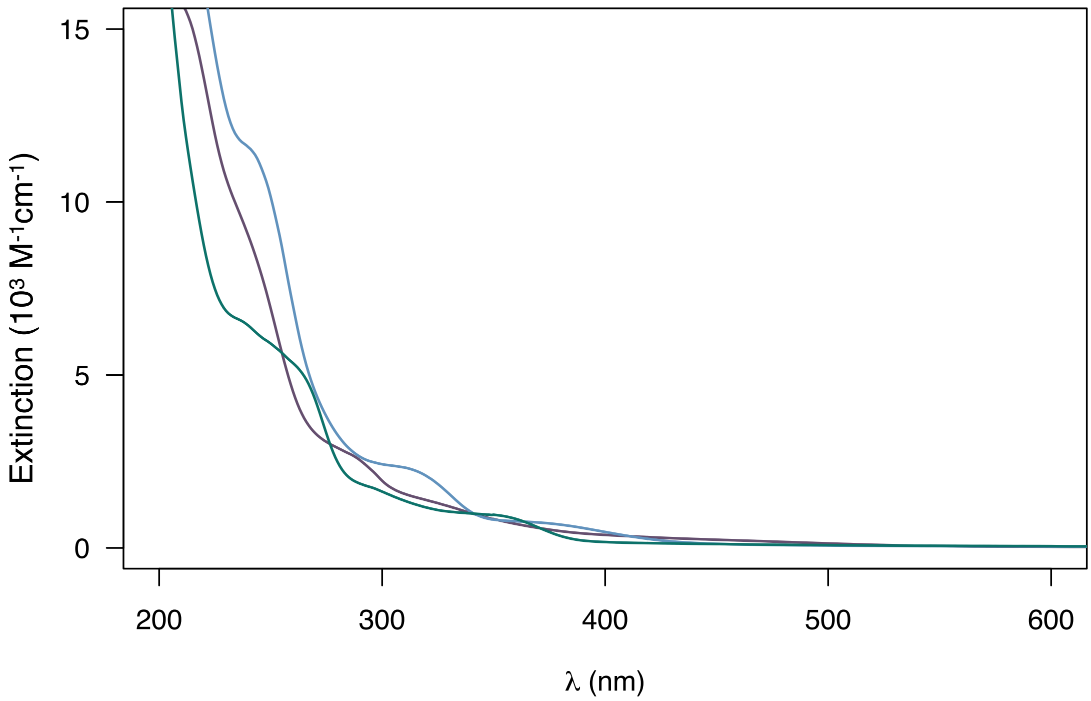

Figure S48. UV-Vis spectra of **1-Ni** (purple), **1-Pd** (blue), and **1-Pt** (green).

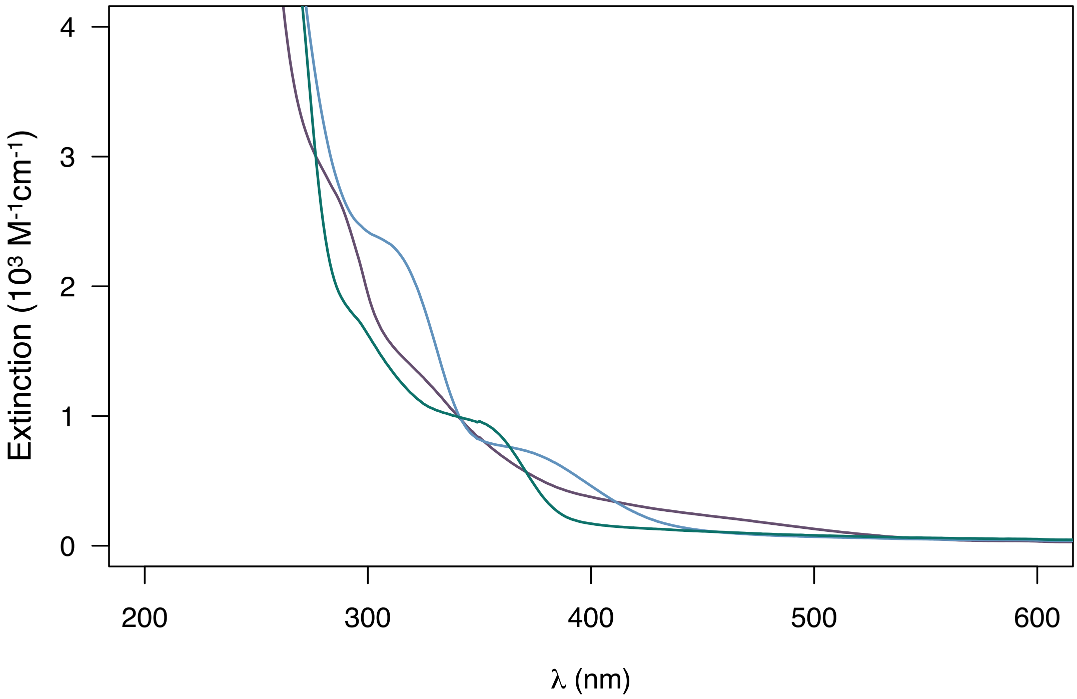

Figure S49. UV-Vis spectra of **1-Ni** (purple), **1-Pd** (blue), and **1-Pt** (green), zoomed for better visibility of the range around 300–500 nm.

9 Temperature-Programmed Reduction (TPR)

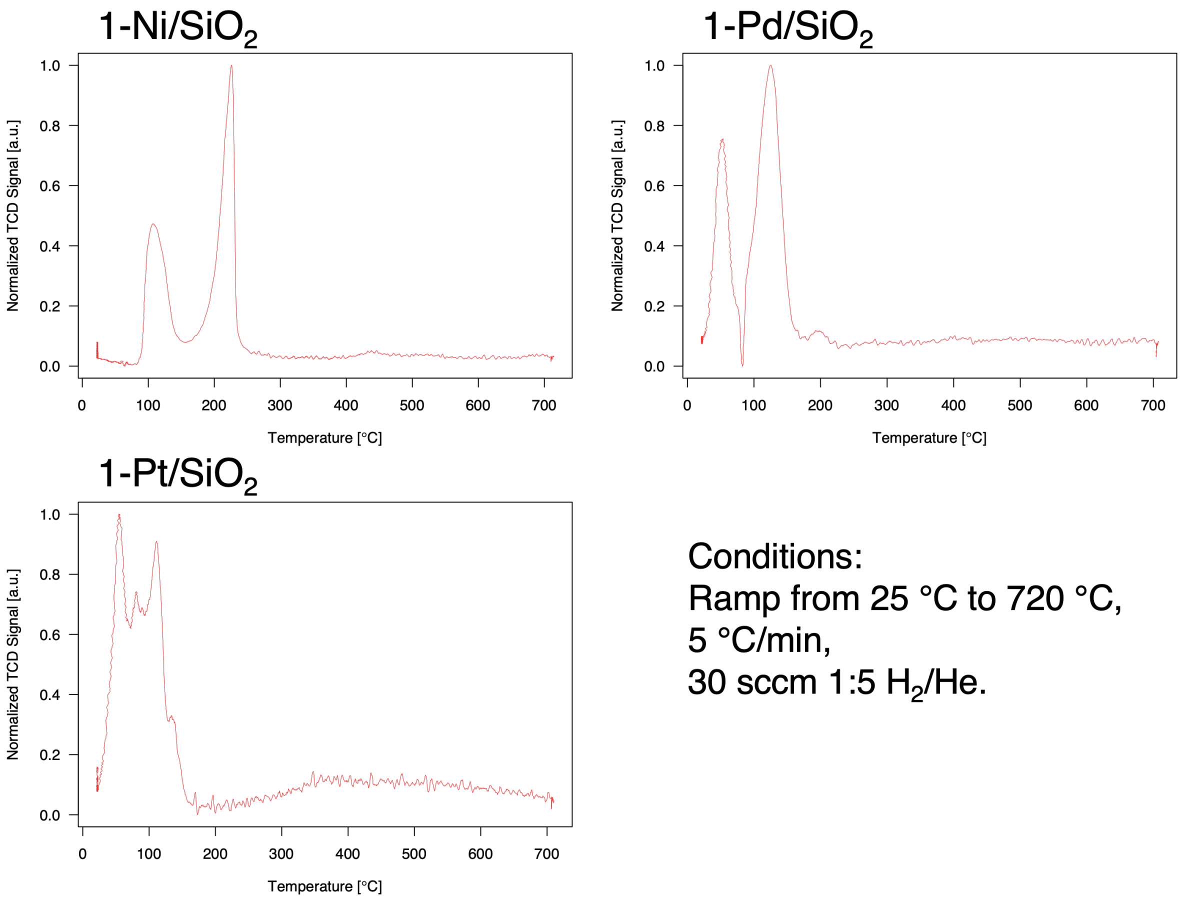

Figure S50. TPR-TCD of **1-Ni**/SiO_{2-700}, **1-Pd**/SiO_{2-700}, and **1-Pt**/SiO_{2-700}.

10. X-Ray Absorption Spectroscopy

10.1 In Situ XANES Pd K-Edge

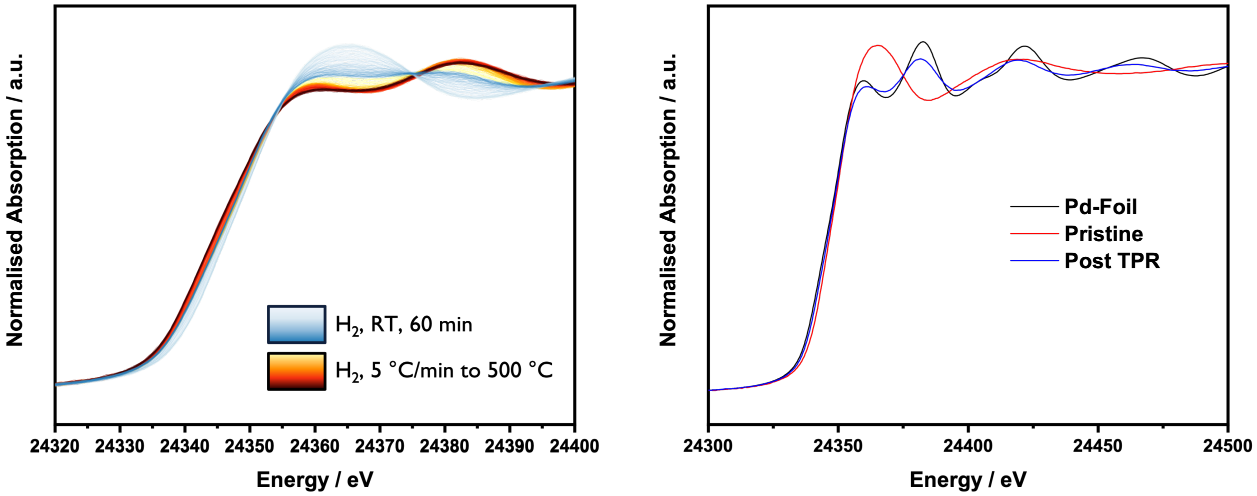

Figure S51. Left: XANES spectra of TPR of **1-Pd**/$SiO_{2\text{–}700}$ at the Pd K-edge. Right: overlay of XANES spectra of **1-Pd**/$SiO_{2\text{–}700}$ (red), after *in situ* reduction (blue), and Pd reference foil (black) at Pd K-edge.

10.2 *Ex Situ* XAS Ni K-Edge

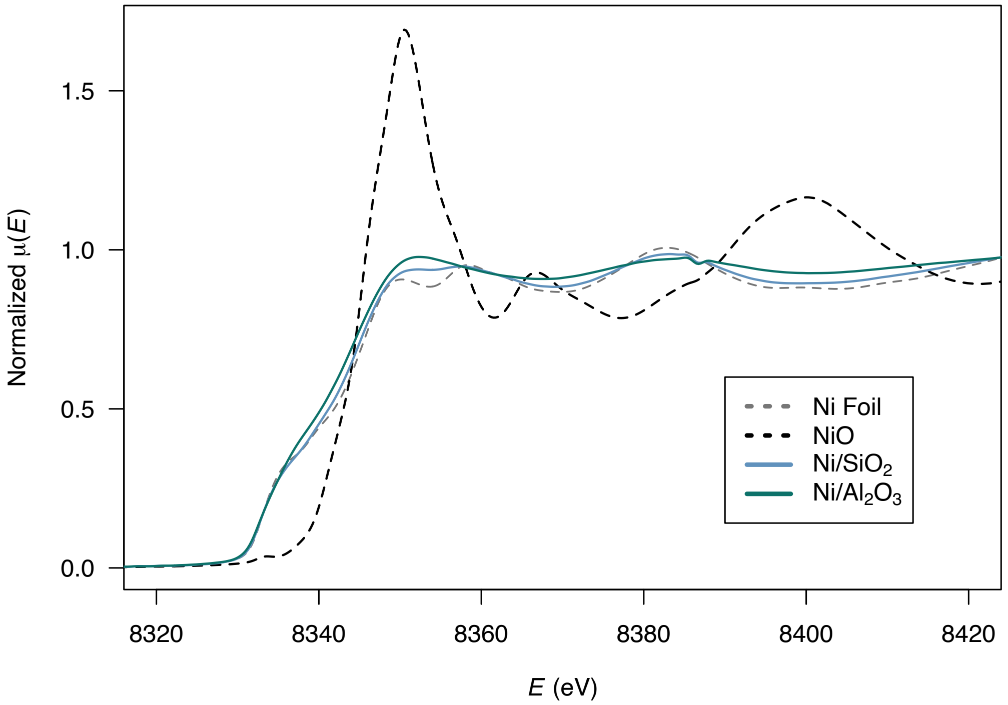

Figure S52. Left: XANES spectra of Ni/SiO_2 (blue) at and Ni/Al_2O_3 (green) at the Ni K-edge, with reference spectra of NiO (black, dashed) and Ni foil (gray, dashed).

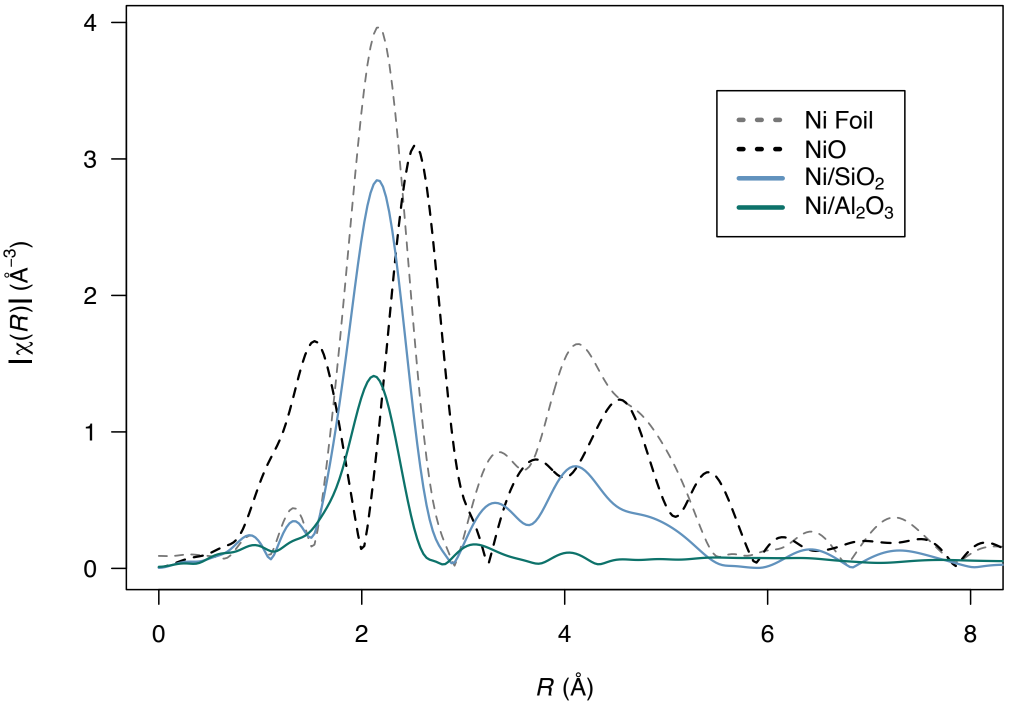

Figure S53. EXAFS data of Ni/SiO_2 (blue) at and Ni/Al_2O_3 (green) at the Ni K-edge, with reference data of NiO (black, dashed) and Ni foil (gray, dashed).

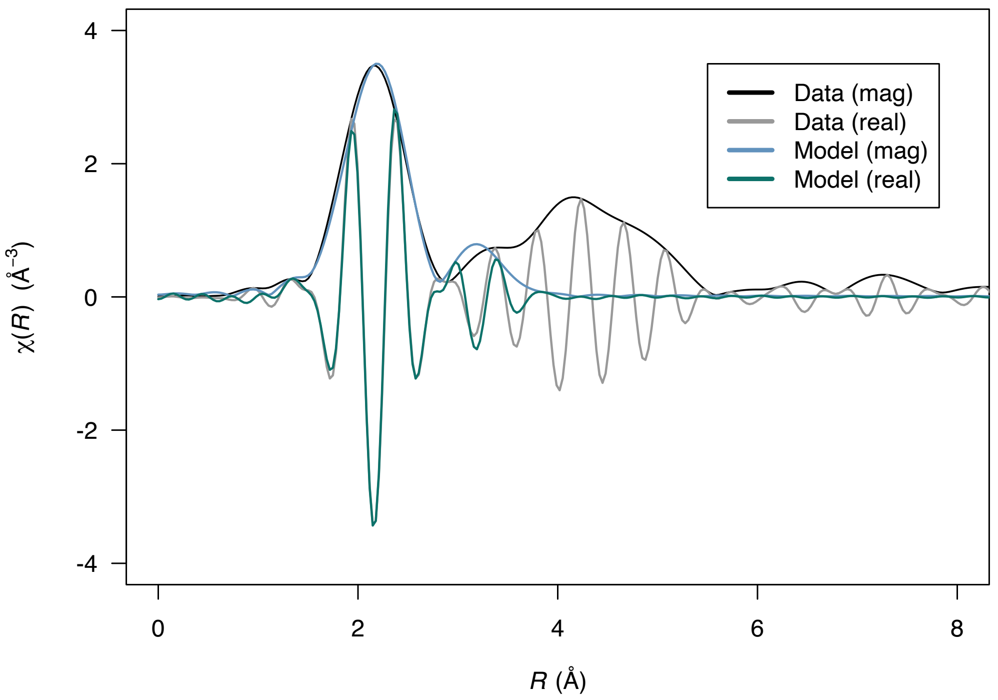

Figure S54. EXAFS fit of Ni reference foil. Parameters in Table S2.

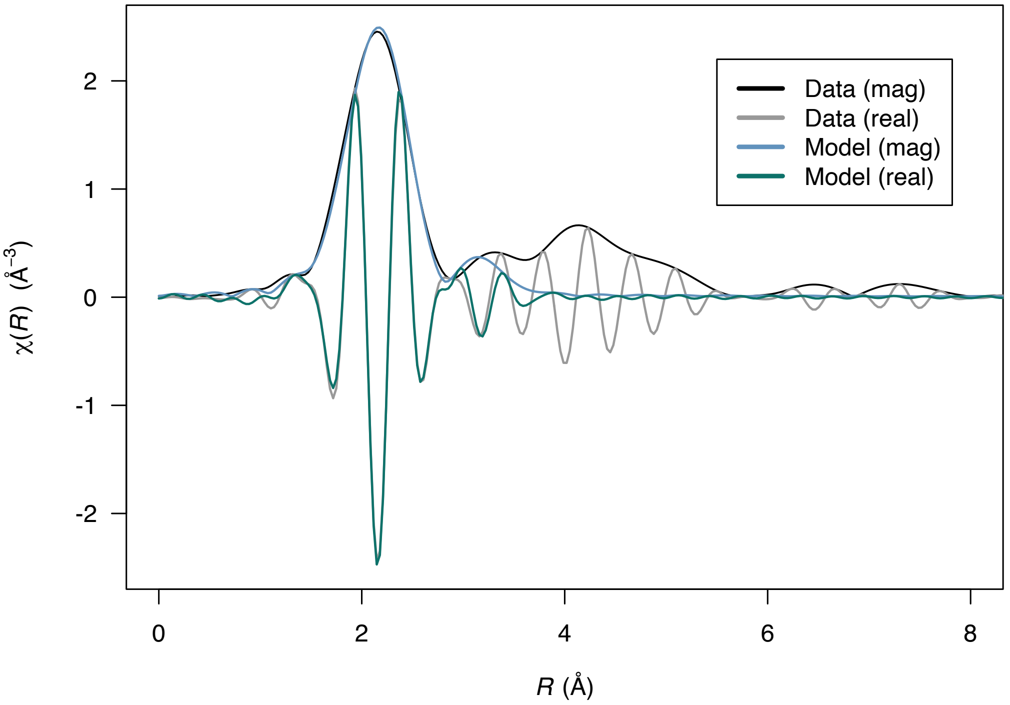

Figure S55. EXAFS fit of Ni/SiO_2. Parameters in Table S2.

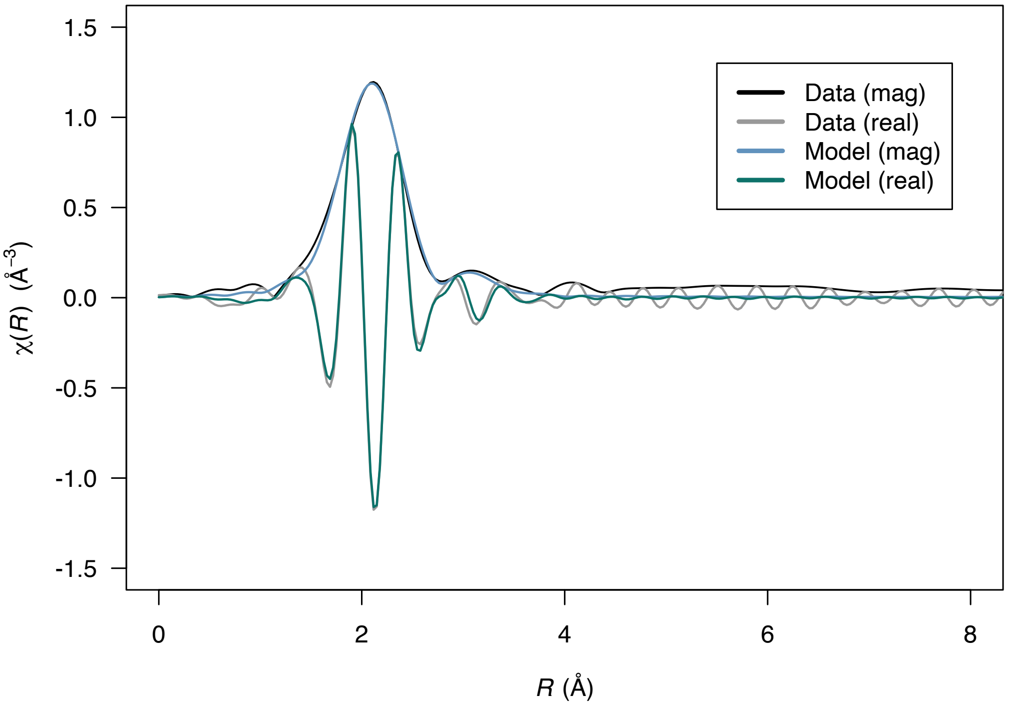

Figure S56. EXAFS fit of Ni/Al_2O_3. Parameters in Table S2.

Table S2. EXAFS fit parameters of Ni reference foil, Ni/SiO_2, and Ni/Al_2O_3.

Path Ni-Ni	*Ni Foil*		*Ni/SiO₂*		*Ni/Al₂O₃*	
s_0^2	0.74	+/- 0.1	0.74	Fixed	0.74	Fixed
Δe_0	9.9	+/- 1.4	8.4	+/- 1.2	5.5	+/- 1.00
R (Ni-Ni)	2.48	+/- 0.01	2.48	+/- 0.01	2.44	+/- 0.01
R2 (Ni-Ni)	3.52	+/- 0.0	3.51	+/- 0.01	3.47	+/- 0.01
CN1	12	Fixed	9.7	+/- 1.1	5.3	+/- 0.5
CN2	6	Fixed	3.0	+/- 1.0	1.3	+/- 0.4
σ^2	0.006	+/- 0.001	0.007	+/- 0.001	0.009	+/- 0.001

10.3 Ni Particle Size Estimation from Coordination Number

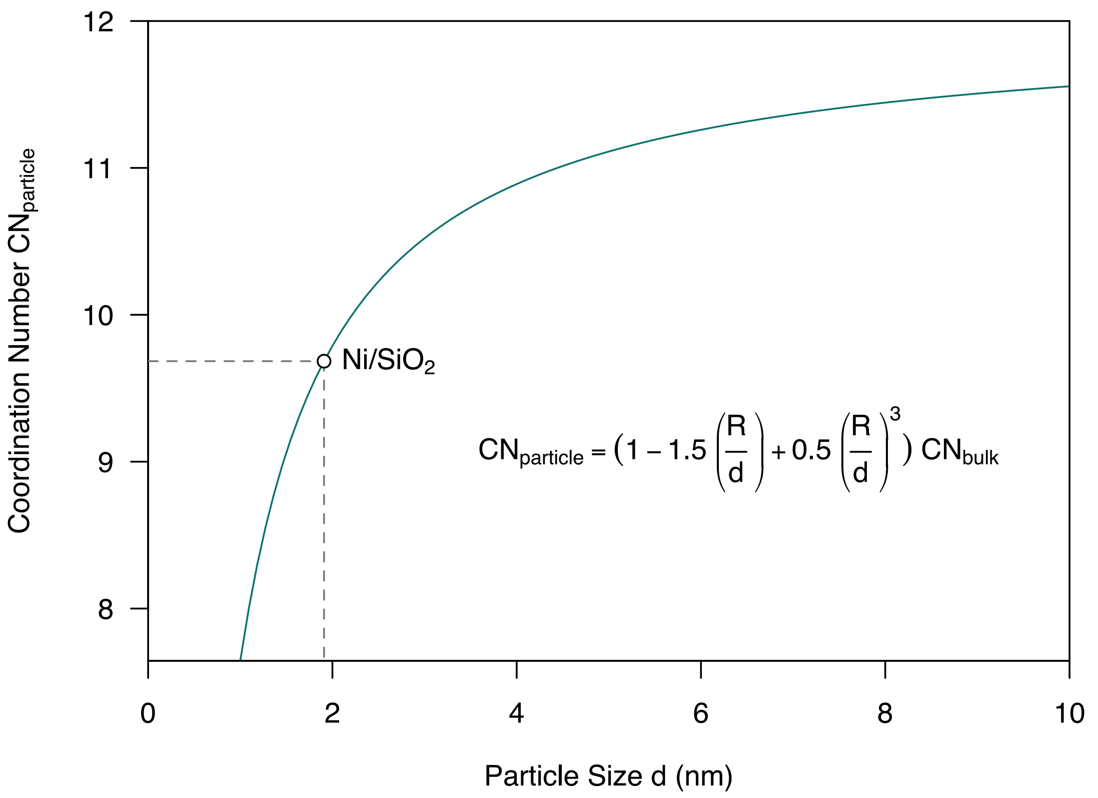

Figure S57. Estimation of particle size using formula indicated in plot where R is the atom distance (2.47 Å), d is the particle size, CN_{bulk} is the fcc coordination number of Ni (12). For Ni/SiO_2 an average particle size of ca. 1.9 nm is estimated, for Ni/Al_2O_3 the particle size is estimated to be below 1 nm, which are both in agreement with HAADF-STEM.

11 CO_2 Hydrogenation

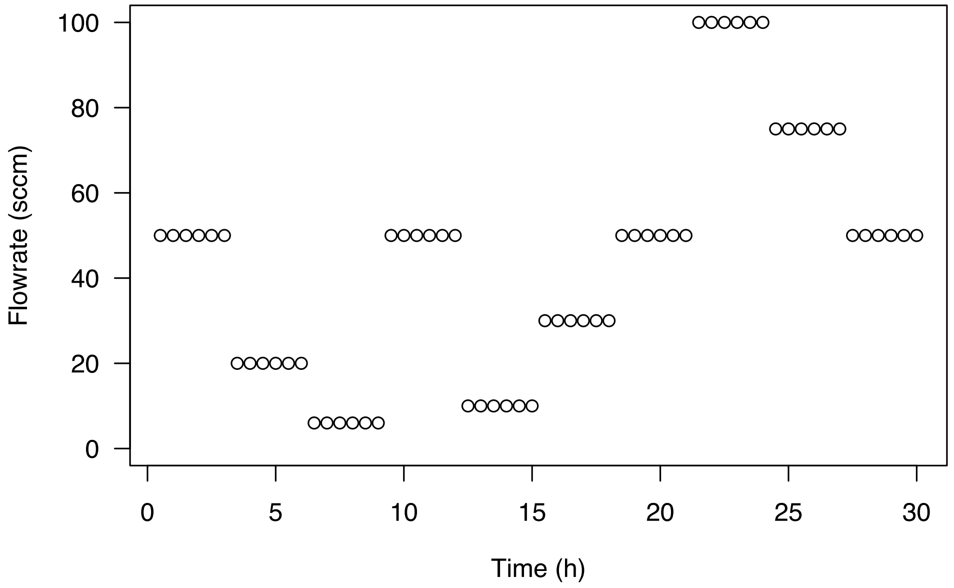

Figure S58. Flowrates of gas mixture during the course of the reaction. For analysis, only the last three points of each flowrate were considered.

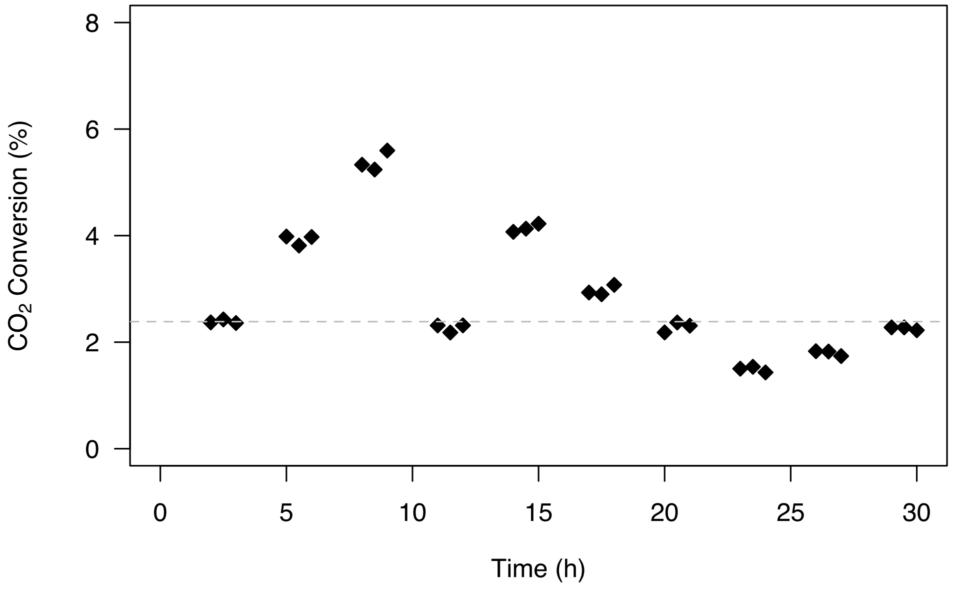

Figure S59. Conversion of CO_2 at different times (see Figure S49 for flowrates). The horizontal dashed line indicates the initial conversion at the first flowrate (50 sccm) which only marginally decreases after 30 h, indicating only minor deactivation.

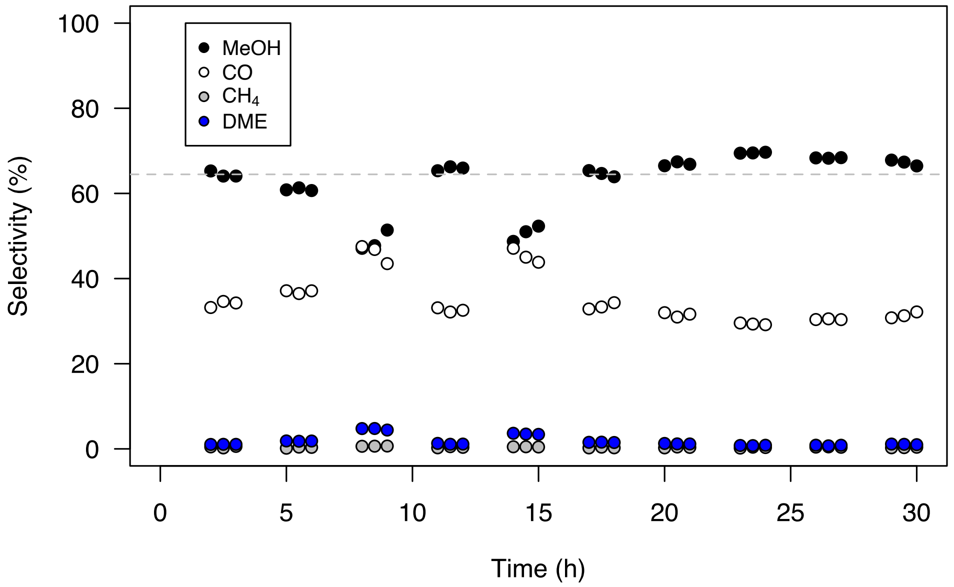

Figure S60. Selectivity for observed products over time, the dashed horizontal line indicates the methanol selectivity at the start of the catalytic run (50 sccm), which slightly increases after 30 h.

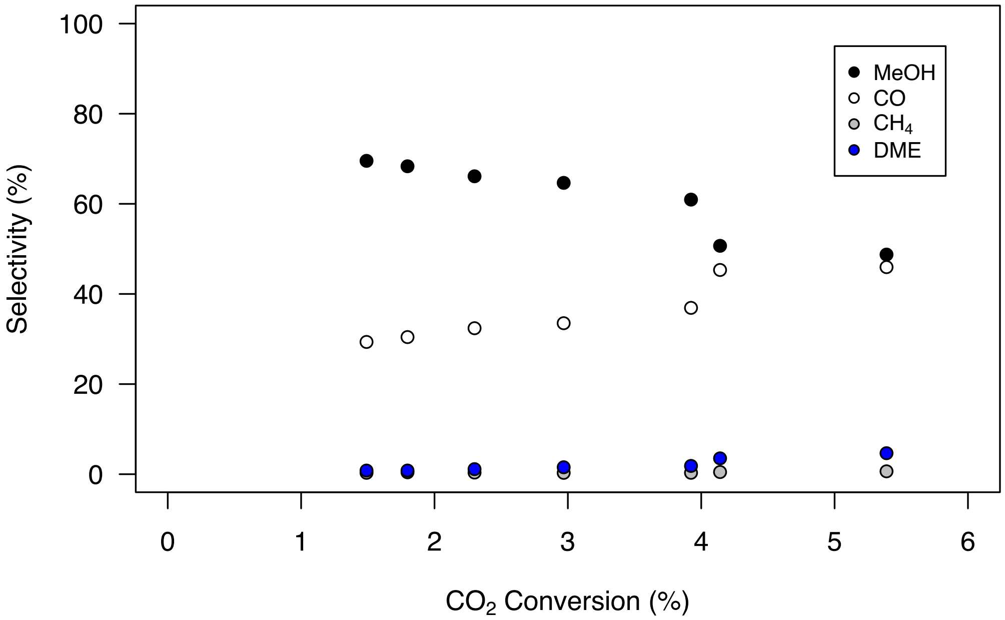

Figure S61. Selectivity for observed products as a function of observed CO_2 conversion.

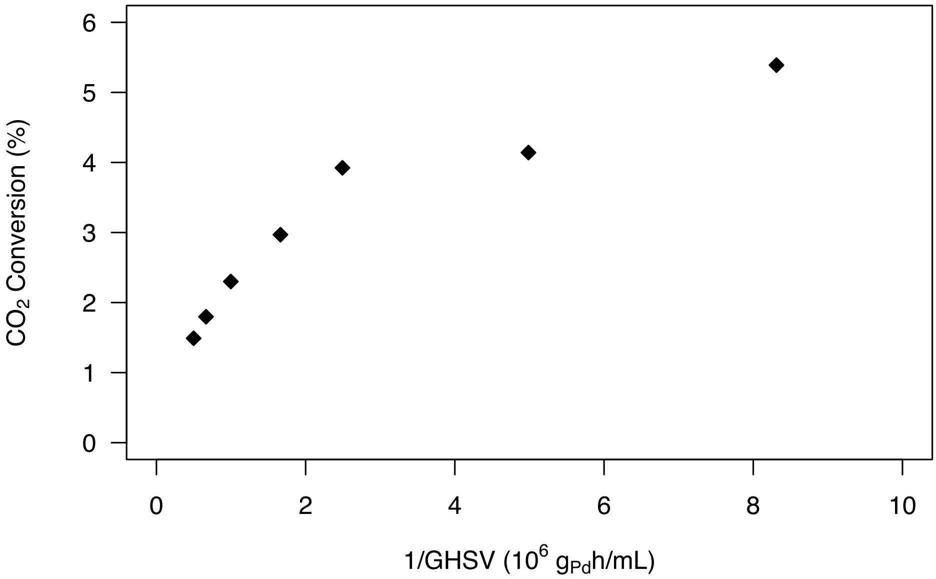

Figure S62. CO_2 conversion as a function of contact time (1/GHSV).

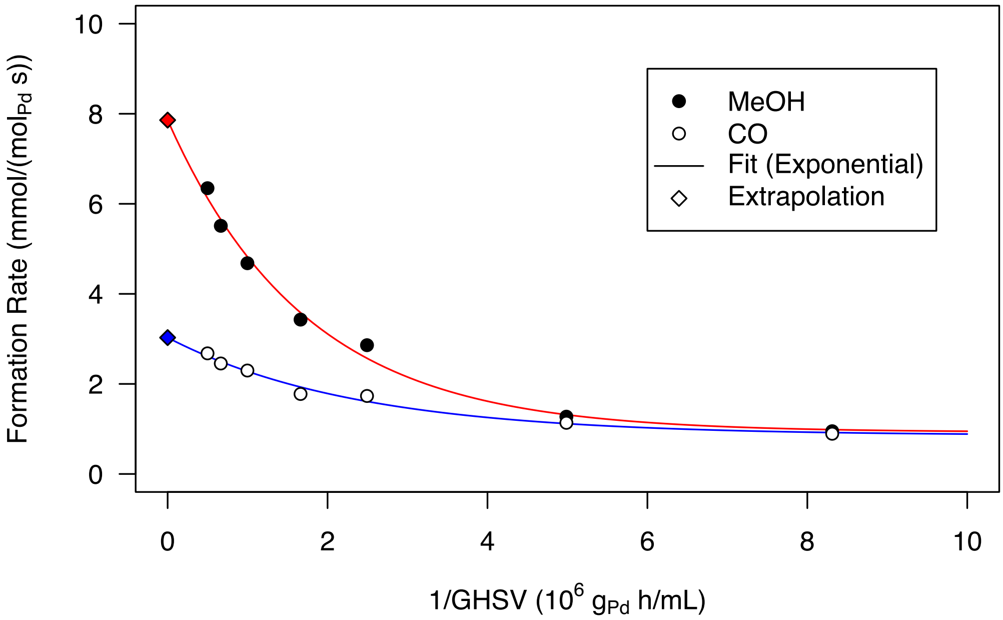

Figure S63. Formation rates of Methanol (black points) and CO (white points), fit using an exponential function (solid lines), and extrapolated values at 1/GHSV = 0 (diamonds) as a function of contact time.

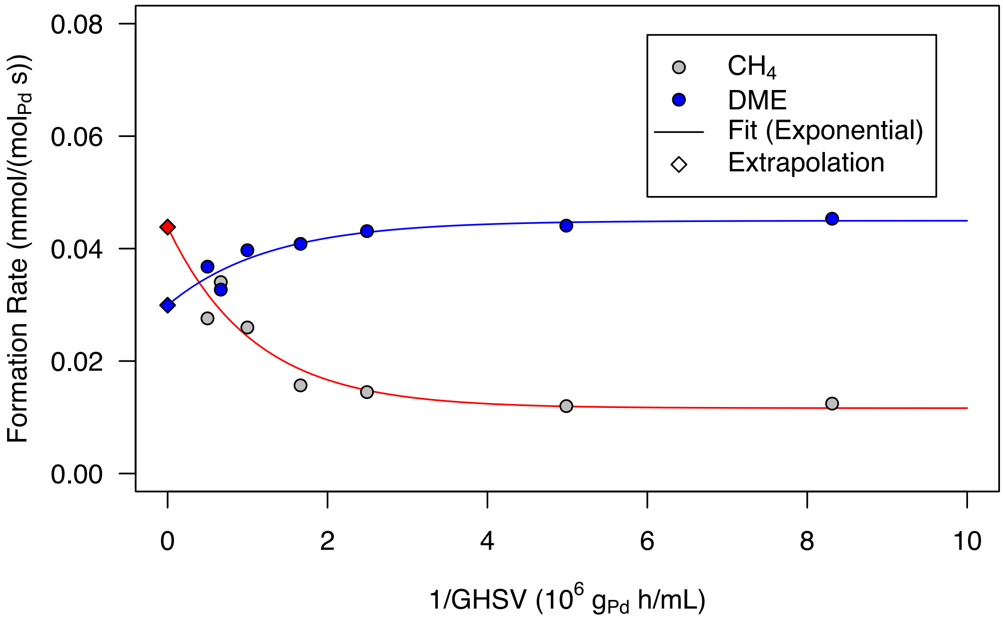

Figure S64. Formation rates of Methane (grey points) and dimethylether (blue points), fit using an exponential function (solid lines), and extrapolated values at 1/GHSV = 0 (diamonds) as a function of contact time.

12 XRD Refinement and Crystal Summary

Table S3. Crystal data and structure refinement for 1-Ni (CCDC: 2241137).

Identification code	exp_1218_auto
Empirical formula	$C_{11}H_{22}N_2Ni$
Formula weight	241.01
Temperature/K	100.00(10)
Crystal system	monoclinic
Space group	P2/m
a/Å	8.6355(4)
b/Å	17.3714(10)
c/Å	8.7132(4)
α/°	90
β/°	96.858(4)
γ/°	90
Volume/Å^3	1297.72(11)
Z	4
ρ_{calc}g/cm^3	1.234
μ/mm^{-1}	1.883
F(000)	520.0
Crystal size/mm^3	0.35 × 0.32 × 0.3
Radiation	Cu Kα (λ = 1.54184)
2Θ range for data collection/°	5.088 to 159.812
Index ranges	$-6 \leq h \leq 10$, $-21 \leq k \leq 20$, $-11 \leq l \leq 11$
Reflections collected	9944
Independent reflections	2836 [R_{int} = 0.0260, R_{sigma} = 0.0216]
Data/restraints/parameters	2836/0/161
Goodness-of-fit on F^2	1.097
Final R indexes [I>=2σ (I)]	R_1 = 0.0374, wR_2 = 0.1015
Final R indexes [all data]	R_1 = 0.0387, wR_2 = 0.1024
Largest diff. peak/hole / e Å^{-3}	1.00/-0.44

Table S4. Crystal data and structure refinement for 1-Pd (CCDC: 2241138).

Identification code	exp_1403_auto
Empirical formula	$C_{11}H_{22}N_2Pd$
Formula weight	288.70
Temperature/K	100.01(10)
Crystal system	monoclinic
Space group	P2/c
a/Å	8.9958(3)
b/Å	17.0021(5)
c/Å	17.7323(6)
α/°	90
β/°	95.316(3)
γ/°	90
Volume/Å^3	2700.45(15)

Z	8
ρ_{calc}g/cm^3	1.420
μ/mm^{-1}	10.828
F(000)	1184.0
Crystal size/mm^3	0.1 × 0.08 × 0.05
Radiation	Cu Kα (λ = 1.54184)
2Θ range for data collection/°	5.198 to 161.162
Index ranges	-11 ≤ h ≤ 10, -21 ≤ k ≤ 21, -22 ≤ l ≤ 22
Reflections collected	39897
Independent reflections	5887 [R_{int} = 0.0542, R_{sigma} = 0.0253]
Data/restraints/parameters	5887/0/259
Goodness-of-fit on F^2	1.054
Final R indexes [I>=2σ (I)]	R_1 = 0.0648, wR_2 = 0.1690
Final R indexes [all data]	R_1 = 0.0691, wR_2 = 0.1718
Largest diff. peak/hole / e Å^{-3}	1.49/-1.33

Table S5. Crystal data and structure refinement for 1-Pt (CCDC: 2241139).

Identification code	CE208_P21_c_FMA
Empirical formula	$C_{11}H_{22}N_2Pt$
Formula weight	377.39
Temperature/K	100.0
Crystal system	monoclinic
Space group	P2$_1$/c
a/Å	11.7804(6)
b/Å	17.5116(8)
c/Å	13.0746(7)
α/°	90
β/°	103.098(2)
γ/°	90
Volume/Å^3	2627.0(2)
Z	8
ρ_{calc}g/cm^3	1.908
μ/mm^{-1}	10.652
F(000)	1440.0
Crystal size/mm^3	0.2 × 0.2 × 0.18
Radiation	MoKα (λ = 0.71073)
2Θ range for data collection/°	4.244 to 54.262
Index ranges	-15 ≤ h ≤ 15, -22 ≤ k ≤ 22, -16 ≤ l ≤ 16
Reflections collected	94565
Independent reflections	5801 [R_{int} = 0.0376, R_{sigma} = 0.0133]
Data/restraints/parameters	5801/281/302
Goodness-of-fit on F^2	1.142
Final R indexes [I>=2σ (I)]	R_1 = 0.0147, wR_2 = 0.0310
Final R indexes [all data]	R_1 = 0.0162, wR_2 = 0.0316
Largest diff. peak/hole / e Å^{-3}	1.03/-0.66

13 References

(1) Searles, K.; Siddiqi, G.; Safonova, O. v.; Copéret, C. Silica-Supported Isolated Gallium Sites as Highly Active, Selective and Stable Propane Dehydrogenation Catalysts. *Chem. Sci.* **2017**, *8* (4), 2661–2666.

(2) Shoukang, H.; Gambarotta, S.; Bensimon, C.; Edema, J. J. H. Ligand Steric Bulk: A Neglected Factor in the Formation of Cr□Cr Supershort Contacts. *Inorg. Chim. Acta* **1993**, *213* (1–2), 65–74.

(3) Zhou, W.; Docherty, S. R.; Ehinger, C.; Zhou, X.; Copéret, C. The Promotional Role of Mn in CO_2 Hydrogenation over Rh-Based Catalysts from a Surface Organometallic Chemistry Approach. *Chem. Sci.* **2023**, *14* (20), 5379–5385.

(4) Schrock, R. R. Preparation and Characterization of $M(CH_3)_5$ (M = Nb or Ta) and $Ta(CH_2C_6H_5)_5$ and Evidence for Decomposition by α-Hydrogen Atom Abstraction. *J. Organomet. Chem.* **1976**, *122* (2), 209–225.

(5) Sheldrick, G. M. A Short History of *SHELX*. *Acta Crystallogr. A* **2008**, *64* (1), 112–122.

(6) Sheldrick, G. M. *SHELXT* – Integrated Space-Group and Crystal-Structure Determination. *Acta Crystallogr. A Found. Adv.* **2015**, *71* (1), 3–8.

(7) Sheldrick, G. M. Crystal Structure Refinement with *SHELXL*. *Acta Crystallogr. C Struct. Chem.* **2015**, *71* (1), 3–8.

(8) Dolomanov, O. v.; Bourhis, L. J.; Gildea, R. J.; Howard, J. A. K.; Puschmann, H. *OLEX2* : A Complete Structure Solution, Refinement and Analysis Program. *J. Appl. Crystallogr.* **2009**, *42* (2), 339–341.

(9) Fulmer, G. R.; Miller, A. J. M.; Sherden, N. H.; Gottlieb, H. E.; Nudelman, A.; Stoltz, B. M.; Bercaw, J. E.; Goldberg, K. I. NMR Chemical Shifts of Trace Impurities: Common Laboratory Solvents, Organics, and Gases in Deuterated Solvents Relevant to the Organometallic Chemist. *Organometallics* **2010**, *29* (9), 2176–2179.

(10) Earl, W. L.; Vanderhart, D. L. Measurement of ^{13}C Chemical Shifts in Solids. *J. Magn. Reson. (1969)* **1982**, *48* (1), 35–54.

(11) Morcombe, C. R.; Zilm, K. W. Chemical Shift Referencing in MAS Solid State NMR. *J. Magn. Reson.* **2003**, *162* (2), 479–486.

(12) Frisch, M. J.; Trucks, G. W.; Schlegel, H. B.; Scuseria, G. E.; Robb, M. A.; Cheeseman, J. R.; Scalmani, G.; Barone, V.; Mennucci, B.; Petersson, G. A.; Nakatsuji, H.; Caricato, M.; Li, X.; Hratchian, H. P.; Izmaylov, A. F.; Bloino, J.; Zheng, G.; Sonnenberg, J. L.; Hada, M.; Ehara, M.; Toyota, K.; Fukuda, R.; Hasegawa, J.; Ishida, M.; Nakajima, T.; Honda, Y.; Kitao, O.; Nakai, H.; Vreven, T.; Montgomery, J. A.; Peralta, Jr. J. E.; Ogliaro, F.; Bearpark, M.; Heyd, J. J.; Brothers, E.; Kudin, K. N.; Staroverov, V. N.; Kobayashi, R.; Normand, J.; Raghavachari, K.; Rendell, A.; Burant, J. C.; Iyengar, S. S.; Tomasi, J.; Cossi, M.; Rega, N.; Millam, J. M.; Klene, M.; Knox, J. E.; Cross, J. B.; Bakken, V.; Adamo, C.; Jaramillo, J.; Gomperts, R.; Stratmann, R. E.; Yazyev, O.; Austin, A. J.; Cammi, R.; Pomelli, C.; Ochterski, J. W.; Martin, R. L.; Morokuma, K.; Zakrzewski, V. G.; Voth, G. A.; Salvador, P.; Dannenberg, J. J.; Dapprich, S.; Daniels, A. D.; Farkas, O.; Foresman, J. B.; Ortiz, J. v.; Cioslowski, J.; Fox, D. J. Gaussian 09.

(13) Adamo, C.; Barone, V. Toward Reliable Density Functional Methods without Adjustable Parameters: The PBE0 Model. *J. Chem. Phys.* **1999**, *110* (13), 6158–6170.

(14) Grimme, S.; Antony, J.; Ehrlich, S.; Krieg, H. A Consistent and Accurate *Ab Initio* Parametrization of Density Functional Dispersion Correction (DFT-D) for the 94 Elements H-Pu. *J. Chem. Phys.* **2010**, *132* (15), 154104.

(15) Dolg, M.; Wedig, U.; Stoll, H.; Preuss, H. Energy-adjusted *ab initio* Pseudopotentials for the First Row Transition Elements. *J. Chem. Phys.* **1987**, *86* (2), 866–872.

(16) Andrae, D.; Häussermann, U.; Dolg, M.; Stoll, H.; Preuss, H. Energy-Adjusted ab Initio Pseudopotentials for the Second and Third Row Transition Elements. *Theor. Chim. Acta* **1990**, *77* (2), 123–141.

(17) Martin, J. M. L.; Sundermann, A. Correlation Consistent Valence Basis Sets for Use with the Stuttgart–Dresden–Bonn Relativistic Effective Core Potentials: The Atoms Ga–Kr and In–Xe. *J. Chem. Phys.* **2001**, *114* (8), 3408–3420.
(18) Weigend, F.; Ahlrichs, R. Balanced Basis Sets of Split Valence, Triple Zeta Valence and Quadruple Zeta Valence Quality for H to Rn: Design and Assessment of Accuracy. *Phys. Chem. Chem. Phys.* **2005**, *7* (18), 3297.
(19) Weigend, F. Accurate Coulomb-Fitting Basis Sets for H to Rn. *Phys. Chem. Chem. Phys.* **2006**, *8* (9), 1057.
(20) Clark, A. H.; Imbao, J.; Frahm, R.; Nachtegaal, M. *ProQEXAFS* : A Highly Optimized Parallelized Rapid Processing Software for QEXAFS Data. *J. Synchrotron Radiat.* **2020**, *27* (2), 551–557.
(21) Newville, M. Larch: An Analysis Package for XAFS and Related Spectroscopies. *J. Phys. Conf. Ser.* **2013**, *430*, 012007.
(22) Wyckoff, R. W. G. *Crystal Structures*; 1963; Vol. 1.
(23) Calvin, S.; Miller, M. M.; Goswami, R.; Cheng, S.-F.; Mulvaney, S. P.; Whitman, L. J.; Harris, V. G. Determination of Crystallite Size in a Magnetic Nanocomposite Using Extended X-Ray Absorption Fine Structure. *J. Appl. Phys.* **2003**, *94* (1), 778–783.
(24) Le Valant, A.; Comminges, C.; Can, F.; Thomas, K.; Houalla, M.; Epron, F. Platinum Supported Catalysts: Predictive CO and H_2 Chemisorption by a Statistical Cuboctahedron Cluster Model. *J. Phys. Chem. C* **2016**, *120* (46), 26374–26385.
(25) Drault, F.; Comminges, C.; Can, F.; Pirault-Roy, L.; Epron, F.; Le Valant, A. Palladium, Iridium, and Rhodium Supported Catalysts: Predictive H_2 Chemisorption by Statistical Cuboctahedron Clusters Model. *Materials* **2018**, *11* (5), 819.
